# Validation and analysis of 12,000 AI-driven CAR-T designs in the *Bits to Binders* competitions

**DOI:** 10.64898/2026.03.03.709355

**Published:** 2026-03-04

**Authors:** Clayton W. Kosonocky, Alex M. Abel, Aaron L. Feller, Amanda E. Cifuentes Rieffer, Phillip R. Woolley, Jakub Lála, Daryl R. Barth, Tynan Gardner, Bits to Binders Competitors, Stephen C. Ekker, Andrew D. Ellington, Wesley A. Wierson, Edward M. Marcotte

**Affiliations:** 1. Department of Molecular Biosciences, University of Texas at Austin, Austin, Texas, USA; 2. The BioML Society, Austin, Texas, USA; 3. LifEngine Animal Health (LEAH) Laboratories Incorporated, Minneapolis, Minnesota, USA; 4. Department of Materials, Imperial College London, London, United Kingdom; 5. Department of Pediatrics, Dell Medical School, University of Texas at Austin, Austin, TX, USA

## Abstract

Artificial intelligence (AI) methods for proteins have advanced rapidly, improving structure prediction and design, particularly for *de novo* binders. However, most evaluations emphasize binding affinity rather than higher-order biological function. We present *Bits to Binders*, a global competition benchmarking *de novo* binder design in the context of chimeric antigen receptor (CAR) T cells. Teams from 42 countries submitted 12,000 designs of 80-amino acid binders targeting human CD20 as CAR binding domains. Designs were screened by pooled CAR-T proliferation, identifying 707 designs exhibiting significant CD20-specific enrichment, with team hit rates from 0.6% to 38.4%. Top-performing candidates were validated as individual constructs, measuring CD20-specific proliferation, expansion, cytokine production, and targeted cell lysis. We identified common design methodologies and factors correlated with DNA synthesis, expression, and target-specific T cell activation which nearly double the success rates when applied as a retrospective filter. We release this dataset as an open resource, with practical recommendations to support more effective AI-driven binder design.

## Introduction

The application of artificial intelligence (AI) methods to proteins has shown remarkable success in recent years^1^. One application area, protein binder design, has garnered significant interest due to its demonstrated tractability and potential to create antibody-like therapeutics^2^. Many AI-driven models have been validated on their ability to design proteins that bind to arbitrary targets, with reported success rates exceeding 10% of tested designs. Despite this progress, the best practices for protein binder design are not yet standardized and it is unclear which computational approaches are most effective across diverse contexts.

Regularly occurring benchmarks like the Critical Assessment of Structure Prediction (CASP) and the Critical Assessment of Predicted Interactions (CAPRI) have been essential in advancing protein structure prediction models to their current level of performance^3,4^. Similar efforts are emerging in AI-driven protein design, such as the Protein Engineering Tournament and Adaptyv Bio’s EGFR binder challenge, which validate crowd-sourced designs against measurable objectives such as binding affinity and activity, providing insight into method performance^5,6^. While these competitions have been instrumental in assessing AI-driven tools for designing proteins that bind, the suitability of designs for other functional endpoints remains largely unknown.

Binding is one of several important features of immune function, and engineered binding by Chimeric Antigen Receptor (CAR) T Cells is of great therapeutic import^7^. CAR-T cells are derived from a patient’s normal T cells through introduction and expression of a chimeric T cell receptor, where the extracellular domain enables the recognition of a targeted tumor surface antigen^8,9^. Upon target engagement by that antigen, receptor clustering occurs, and downstream pathways are activated that drive T cell proliferation, cytokine secretion, and cytotoxic killing.

Prior work has demonstrated the general feasibility of AI-designed CAR binding domains^10-13^, and thus this system provides an excellent opportunity for evaluating different design approaches in a competition setting. Designing binders in the context of a CAR-T cell requires construct expression and membrane localization of the receptor that is compatible with cell viability and engages downstream signaling upon target recognition, leading to proliferation, expansion, cytokine production, and target cell lysis. We created the *Bits to Binders* competition which engaged a total of 28 teams to generate 12,000 protein binders that targeted the lymphoma surface antigen CD20, to be synthesized and assayed for CAR-T cell function. From the resulting dataset, we extracted design principles and predictive metrics that contribute to functional binder performance in the context of a CAR-T cell therapy, with the goal of moving the field closer to standardized and effective methods for AI-driven protein design.

## Results

### Crowd-sourcing *de novo* designed CD20-binding CARs for the *Bits to Binders* competition

**Figure 1.**
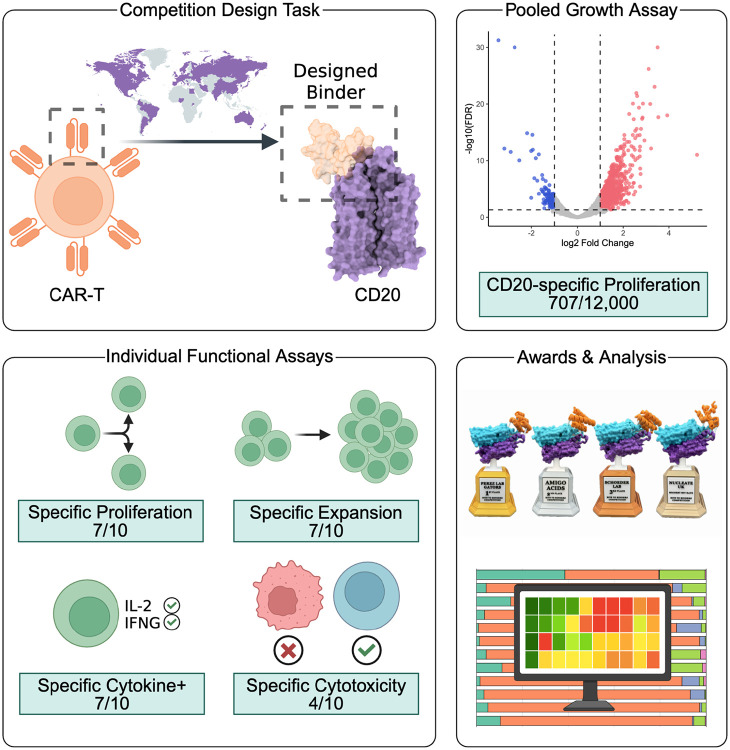
Overview of the *Bits to Binders* competition. Competitors from around the world used AI-driven protein design methods to design 12,000 proteins that bind CD20 and initiate a T cell immune response when used as the binding domain of a CAR-T cell. The 12,000 designs were tested in a competitive growth assay, in which CD20-specific CAR-T cell proliferation was measured. The designs with the greatest CD20-specific fold change in growth were tested individually for CD20-specific proliferation, expansion, cytokine production, and target cell lysis. The results were then analyzed to identify computational features leading to increased success rates for AI-driven CAR design. Figure 1 was created using BioRender (https://biorender.com).

Competitors from 42 countries were tasked to use their chosen AI-driven protein design methods to design 500 80-amino-acid binders to the extracellular domain of human CD20 that could be accommodated in the context of a CAR (Fig. 1). This task differed from a pure binder design task in several ways. First, while the competitors submitted only binding domains, they were aware that these should eventually be compatible with the greater CAR scaffold, in particular the transmembrane domain. Second, success was dependent on achieving sufficient affinity to effectively recognize the target, but not so much that the CAR-T cell becomes exhausted^14,15^. And finally, success would ultimately depend on CAR-T function, not just binding. These nuances, along with a primer on CAR-T biology, the exact CAR scaffold used, and an in-depth explanation of all assays were conveyed to the participants during the solicitation (Supplementary Information C). In addition, several research and review articles on AI-driven protein design, CAR-T therapies, and CD20 biology were provided to the participants (Supplementary Information C).

At the end of the six-week design stage, a total of 12,000 designs were collected. The approaches used spanned a wide range of generative design and selection strategies (Supplementary Information E). Generative diffusion models were the most common approach, with 18 teams using RFdiffusion^16^ and two teams using sequence-structure co-design models like Chroma^17^. The non-diffusion approaches included constraint- or hallucination-based design such as BindCraft^18^ (3 teams), EvoBind^19^ (2), and ColabDesign (3), as well as protein language models like PepMLM^20^ (1), ESM-2^21^ (2), and RayGun^22^ (1). Fixed backbone design tools were widely used for sequence generation and diversification, notably ProteinMPNN^23^ (19 teams) followed by SolubleMPNN^24^ (2) and CARBonAra^25^ (1). Twelve teams generally followed the default RFdiffusion–ProteinMPNN–AlphaFold2 pipeline, while nine teams opted to perform “iterative diffusion” in which the initial binder was generated, the low-scoring segments were removed, and the process was repeated until the scores were sufficient.

Many teams conditioned their generative models on CD20 in the Rituximab-bound state (PDB: 6VJA) or by starting with fragments of CD20-specific antibodies. Initial structures were frequently refined using FastRelax^26^, GROMACS^27^, or OpenMM^28^. After generation, it was common to select sequences using scores, structure prediction models, or other bioinformatics tools. To confirm that generated sequences were predicted to fold into the desired structures, AlphaFold2^29^ was used by 17 teams, with others using ColabFold^30^ (4), ESMFold^21^ (4), Chai-1^31^ (1), or HelixFold^32^ (1). Interestingly, only one team reported folding their designs in the context of the entire CAR sequence. Other selection criteria included Rosetta energy scores^33,34^, HDOCK scores^35^, and binding affinity predictions from Prodigy^36^. The wide variety of inputs, model types, and scoring strategies resulted in substantial sequence diversity between teams: over 83% of submissions did not produce a significant MMseqs2^37^ alignment to sequences from other teams (Fig. SA1). In contrast, within-team sequence redundancy was relatively high, with 20% of sequences aligning nearly perfectly to another design from the same team (≥0.95 MMseqs2 identity).

### Screening 12,000 *de novo* CAR binding domains in a T cell proliferation assay

**Figure 2.**
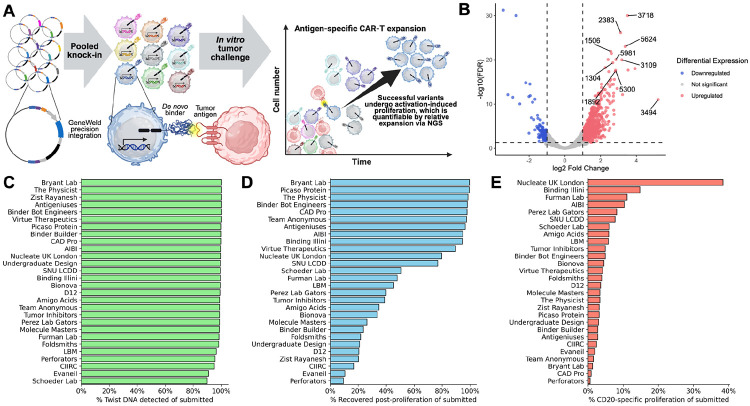
Pooled proliferation assay identifies designs with CD20-specific CAR-T cell response. **(A)** CD20 binder transgenes are knocked into a population of T cells, which is then subjected to either a tumor challenge or a control condition. Target-specific T cell proliferation indicates antigen recognition and is quantified as the relative change in abundance of each transgene sequence in the tumor challenge condition compared with the control. **(B)** Of 12,000 binders, 707 showed a significant CD20-specific ≥2-fold increase in growth, indicating potential recognition of CD20 by the binder. **(C)** Percent of each team’s submitted sequences detected in the plasmid pool during Twist quality control. **(D)** Percent of each team’s submitted sequences with ≥25 reads in each of the six proliferation assay replicates. Post-proliferation recovery suggests that the binder produced a viable CAR-T cell. **(E)** Percent of each team’s submitted sequences with significant ≥2-fold change in CD20-specific proliferation. Panel A was created using BioRender (https://biorender.com).

To functionally evaluate the 12,000 binder designs we employed a pooled CAR-T challenge assay that measures antigen-dependent T cell proliferation (Fig. 2A). Pools of codon-optimized DNA fragments encoding the 80-amino-acid binding domain were synthesized and cloned into a plasmid encoding a second-generation CAR backbone by replacing the binder region of a second-gen CAR28z construct (**Methods**). The plasmid library was used as a DNA donor for targeted integration of the CAR at the T cell receptor alpha chain (TRAC) to engineer a pool of TRAC(−), CAR(+) T cells^38,39^.

The sequence of the integrated, designed binder in each CAR construct served as its own unique DNA barcode for each cell. To enrich for T cells carrying integrations of the pooled CAR library and negatively select unedited T cells, we subjected the population to methotrexate selection^40,41^ via the co-expressed DHFR^F/S^ variant. After selection and expansion, the remaining CAR-expressing cells were co-cultured either alone or with the CD20-positive Raji cell line^42^, both in triplicate. Enrichment of binders was measured by the relative change in “barcode” sequences after two weeks in Raji co-culture compared to those that were cultured without CD20+ targets. Enrichment or depletion in the non-target group may be indicative of dysregulation in CAR-T signaling or decreased cellular fitness independent of binding. Thus, comparing the Raji co-culture with this group allows the effect of CD20+ targets on proliferation to be robustly quantified.

NGS reads were quantified across the three technical replicates to identify significant CD20-specific enrichment (Fig. 2B, **Methods**), with few jackpot effects occurring between the replicates (Fig. SA2). Across the 12,000 designs, 98.3% passed Twist’s DNA quality check (Fig. 2C), but only 56.8% passed the minimum read thresholds after growth. Given that a functional CAR is required to provide the basal signaling required for sustained T cell fitness and survival, this drop-off indicates that many of the designs may have failed to yield a valid receptor (Fig. 2D).

Ultimately, 707 clones showed significant CD20-specific enrichment of at least twofold relative to controls. Hit rates varied substantially between teams, ranging from 0.6% to 38.4% of submitted sequences (Fig. 2E). The top ten non-redundant designs were chosen to proceed to a series of individual T cell functional assays.

### Confirming function with individual T cell assays

**Figure 3.**
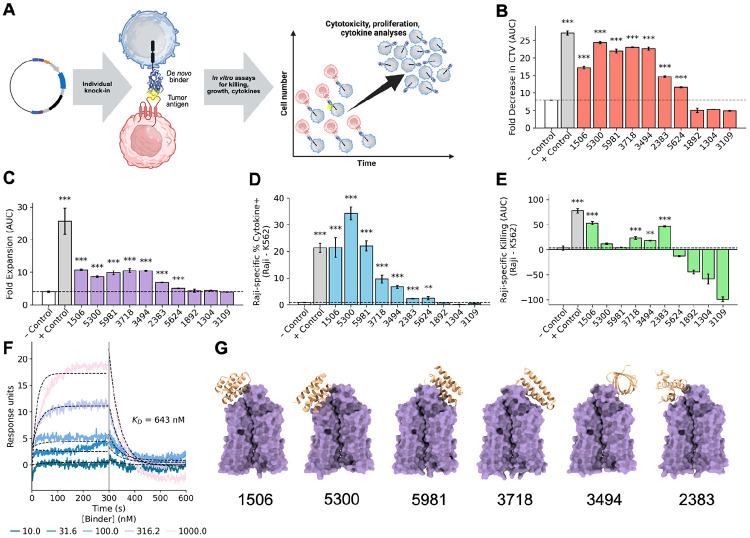
*In vitro* assays confirm designed binders function as CAR-T cells. **(A)** DNA constructs of designed binders are integrated into primary T cells for functional analysis against CD20+ tumor cells. **(B)** CD20-specific proliferation, computed by AUC of CTV dilution (Raji - K562) across four E:T ratios (Fig. SA4). Bars show mean CTV MFI ± SE; significance by BH-corrected one-sided Welch’s t-test vs. negative control. **(C)** CD20-specific expansion, computed as AUC of fold-expansion (Raji - K562) across four E:T ratios (Fig. SA6). Bars show mean ± SE; BH-corrected one-sided Welch’s t-test vs. negative control. **(D)** CD20-specific production of IL-2 and IFNγ. Bars show mean percent of cytokine+ cells in the vs-Raji condition ± SE; BH-corrected one-sided Welch’s t-test vs. negative control. **(E)** CD20-specific cytotoxicity, computed as AUC (Raji - K562) across three E:T ratios (Fig. SA8). Bars show mean ± SE; BH-corrected one-sided z-test vs. negative control. **(F)** SPR measurements for design 2383 indicate moderate binding affinity to detergent-solubilized CD20 when expressed as an isolated 80-mer (*K*_*D*_ = 643nM). **(G)** Boltz-1 structures of selected CD20-bound designs that broadly outperformed the negative control. Significance stars relative to negative controls: *, p < 0.05; **, p < 0.01; ***, p < 0.001. Panel A was created using BioRender (https://biorender.com).

Each of the top ten binders were cloned as individual CAR constructs in a manner identical to how they would have appeared in the pooled library. CAR-T cells expressing each of these binders were characterized in a series of assays probing different aspects of T cell activation and effector function, including assays for expression, proliferation, expansion, cytokine production, and target cell lysis (Fig. 3A). CD20-targeting, scFv-based CAR-T and untransfected TCR+ T cells served as positive and negative controls, respectively.

Flow cytometry was used to immunophenotype TCR and CAR expression (Fig. SA3A, SA3B). Seven of the designs (1506, 1892, 3494, 3718, 5300, 5624, 5981) proved to be CAR+ cells, with 10-50% of the population being CAR+ cells (Fig. SA3D). Design 2383 had <5% CAR+ cells, while 1304 and 3109 all but lacked expression (Fig. SA3D).

CAR-T cell cumulative expansion and cell division were evaluated five days after stimulation with CD20-positive target cells at varying effector-to-target ratios. Seven clones outperformed the CAR-negative untransfected control T cells in expansion and proliferation (Fig. 3B, 3C, SA4, SA5, SA6; clones 1506, 2383, 3494, 3718, 5300, 5624, 5981). The remaining three designs (1304, 1892, 3109) underperformed or were equal to the negative control.

Target-specific production of the cytokines IL-2 and IFNγ was assessed with flow cytometry, and the same seven designs outperformed the negative control when cultured with CD20+ targets (Fig. 3D, SA7). Clones 1506, 5300, and 5981 showed the most activity, generating similar, if not more, cytokines than the scFv control. The remaining three designs (1304, 1892, 3109) once again did not outperform the negative control.

Finally, specific lysis of CD20+ target cells over 48 hours was evaluated by co-incubating CAR-T cells with CD20-positive target cells (Raji-GFP) and CD20-negative controls (K562-GFP) at varying effector-to-target ratios. Although the same seven designs outperformed the negative control in terms of non-specific cytotoxicity, lysing both CD20+ and CD20− target cell lines at the measured E:T ratios, only four of these (1506, 2383, 3494, 3718) had significant, specific lysis of CD20+ cell lines above the negative control (Fig. 3E, SA8).

### Characterization of binding

Activation in the presence of CD20+ Raji cells implies that there were productive interactions with the designed binders, so we further assessed binding via a surface plasmon resonance assay in which detergent-solubilized human CD20 was flowed across the immobilized 80-amino-acid designs. The known scFv was used as a positive control and was found to have a *K*_*D*_ of 1.75nM (Fig. SA9). Of the top ten binders, only three were found to have appreciable binding in this assay: 2383 had a *K*_*D*_ of 643nM and designs 1506 and 5981 had detectable binding but weak signal, estimated by Anabel^43^ to be between 200-600nM (Fig. 3F, Fig. SA9). This is perhaps unsurprising, given that optimal CAR function often occurs within a moderate range of binding constants; if binding is too high, trogocytosis and T cell exhaustion can occur and many lower affinity CARs have been found to elicit good if not better tumor elimination ^44,45^.

### Competition winners and outcomes of methodological choices

Overall, design 2383 from Nucleate UK London bound strongly to CD20, had high specific lysis, moderate proliferation and expansion, low cytokine %, and low CAR+ %. Design 1506 from Perez Lab Gators had weaker binding but high specific lysis, cytokine %, proliferation, expansion, and CAR+%. Design 5981 from the Schoeder Lab also had weaker binding and high proliferation, expansion, and CAR+%, but had strong lysis with weak specificity. Designs 3494, 3718, 5300, and 5624 (from SNU LCDD, SNU LCDD, Amigo Acids, and Binding Illini, respectively) had no measured binding despite being broadly functional, with two of the four having target-specific lysis, and all four having overall lysis, suggesting that these designs may have largely relied on avidity effects or mechanisms other than CD20 recognition. Designs 1304 and 3109 did not express, while design 1892 expressed but failed all functional assays. One conclusion is thus that 7 of the top 10 designs were successful in functional assays, suggesting that a variety of generative design approaches (from teams Nucleate UK London, Perez Lab Gators, Amigo Acids, Schoeder Lab, SNU LCDD, and Binding Illini) can yield functional CAR-T cells.

**Figure 4.**
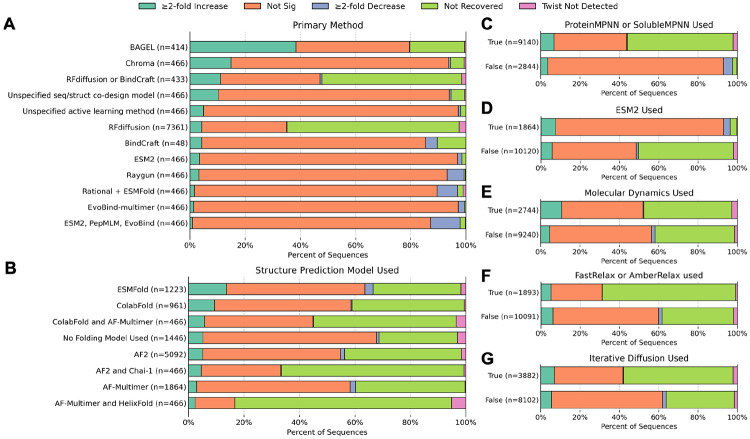
Experimental outcomes based on methodological choices. Methodological choices were categorized into: **(A)** choice of primary generative method, **(B)** choice of structure prediction model, **(C)** the use of ProteinMPNN or SolubleMPNN, **(D)** the use of ESM-2, **(E)** the use of molecular dynamics prior to or after generation, **(F)** the use of FastRelax or AmberRelax prior to or after generation, and **(G)** the use of diffusion models in an iterative manner. Although some trends are visible, the causality is unclear and may result from artifacts of this categorization failing to capture higher order methodological details.

The choices made by each team in the design process were numerous and provide ample ground for further methodological analysis. The best-performing generative methods were BAGEL^46^ and Chroma, followed by RFdiffusion, BindCraft, and team AIBI’s sequence-structure co-design model (Fig. 4A). Many designs were verified with structure prediction models, with ESMFold in first, then ColabFold and AlphaFold-like models (Fig. 4B). However, as ESMFold’s success is carried by its use in BAGEL, the true causal impact of the structure prediction models is unclear. That said, the effect of ProteinMPNN or SolubleMPNN usage is very strong, with the use of these models causing an increase in CD20-specific enrichment and simultaneous lack of detection in the proliferation assay, as further analyzed below (Fig. 4C, 5B). Conversely, ESM-2 usage resulted mostly in sequences that were recovered in the proliferation assay (Fig. 4D). Using molecular dynamics tools on the structure either before generating to obtain an optimal pose, or afterwards for filtering, correlated with a modest increase in successful designs, though this was not true for FastRelax or AmberRelax (Fig. 4E-F).

There were common methodologies shared between the top-performing teams (Perez Lab Gators, Amigo Acids, Schoeder Lab, Nucleate UK London, SNU LCDD, Binding Illini, and Furman Lab). First, all of these teams used a diffusion model, either RFdiffusion or Chroma. Second, all of these teams used ProteinMPNN or SolubleMPNN for some stage of the design. Third, many of these teams employed these models in an iterative or refinement strategy, although this was found to have minimal impact on success across all teams (Fig. 4G). The teams with the two best-performing binders (Perez Lab Gators, Amigo Acids, Fig. SA10) both had highly similar pipelines employing iterative RFdiffusion, ProteinMPNN and FastRelax. Notably, the team with the top-performing design (Perez Lab Gators, Fig. SA10) was the only team that reported folding their designs within the greater CAR sequence.

### Analysis of factors leading to design failure and success

The diversity of sequences generated during the competition by various methods are themselves a wealth of information on the success of AI-driven tools, as applied by many different users, for designing proteins to modify cellular functionality. The nucleotide and amino acid sequences of the 12,000 designed binders were used to compute over 400 metrics, ranging from simple readouts like sequence entropy to more involved ones such as the average energy of the binder-CD20 interface on a predicted structure (**Methods;** Supplementary Information B). The varied features were further analyzed for their ability to predict (a) detection in the DNA quality report, (b) whether or not the design was recovered by NGS after proliferation, and (c) the CD20-specific change in proliferation.

**Figure 5.**
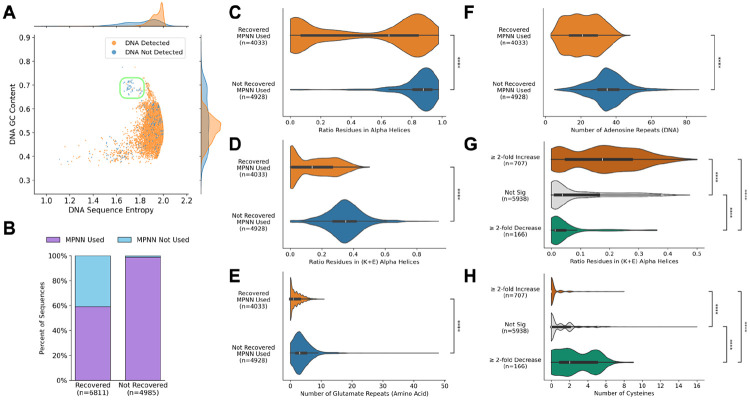
Computational analysis suggests mechanisms leading to success and failure. **(A)** The combination of DNA GC content and Shannon entropy separates out 55 designs that failed to synthesize as DNA. **(B)** Almost all of the designs that failed to be recovered were created with ProteinMPNN or SolubleMPNN. **(C-D)** The MPNN designs that failed to be recovered were enriched in alpha helices that contained mostly lysine and glutamate. **(E-F)** These designs had significant glutamate repeats (EE) in the amino acid sequence and adenosine repeats (AA) in the DNA sequence, both of which have been shown to cause ribosomal termination, suggesting a possible mechanism for the absence of these sequences. **(G)** The designs that caused a ≥2-fold increase in CD20-specific proliferation were enriched in (K+E) alpha helices, though to a lesser extent than those that were not recovered. **(H)** The designs that caused a ≥2-fold decrease in CD20-specific proliferation were enriched in cysteines predicted to form disulfide bonds within the binder chain (Fig. SA11). Significance from two-sided BH-corrected Mann-Whitney-Wilcoxon tests (**** for p ≤ 0.0001).

The Twist plasmid pool quality report indicated that 204 of the 12,000 designs were not detected. Almost all of the failed designs originated from 5 of the 28 teams (71%, Fig. 2C), suggesting that, despite codon optimization, particular design features led to synthesis failures. One common cause seemed to be high GC content and repetitive sequences; a cluster of failed sequences with high GC content and moderately low Shannon entropy can be observed (Fig. 5A). Applying a more stringent filter to remove sequences with <1.85 Shannon entropy and ≥0.65 GC content would have removed a quarter of the DNA failures (55 designs), while having no impact on functional sequences.

Of the synthesized designs, 6,811 were recovered by NGS after the proliferation assay (≥25 reads in all six replicates) and 4,985 were not recovered. As a functional CAR is necessary for sustained T cell fitness and survival, failure in this readout suggests failed protein expression, solubility, or folding. The recovered and non-recovered designs differ markedly in their amino acid compositions, with the non-recovered sequences being preferentially enriched in glutamate, lysine, and alanine (Fig. SA11).

Strikingly, 98.9% of the non-recovered sequences were designed with ProteinMPNN or SolubleMPNN (MPNN), whereas the recovered sequences came from both these models (59.2%) and other methods (40.8%) (Fig. 5B). To determine why some MPNN designs failed where others succeeded, the designs created without MPNN were removed, resulting in 4,033 recovered and 4,928 non-recovered sequences. Categorizing the secondary structure of these designs’ Boltz-1 predicted structures indicated that most of the non-recovered MPNN sequences were almost entirely alpha helices, with an average of 83% of the residues being part of a helix (Fig. 5C). Compared to the alpha helices from the recovered sequences, those from the non-recovered sequences were highly enriched in lysine (K) and glutamate (E) (Fig. 5D). In fact, the ratio of residues in K+E alpha helices was by far the strongest predictor of sequence recovery, averaging 35% of residues for non-recovered MPNN and 15% for recovered MPNN and achieving 0.91 ROC-AUC using a logistic regression model trained with stratified 5-fold cross validation (Table SA1, Fig. SA12, **Methods**).

We thus formulated hypotheses to explain the mechanism behind the importance of K+E alpha helices. Repeats of acidic amino acids are known to directly impact protein synthesis since "nascent chains enriched in aspartic acid (D) or glutamic acid (E) in their N-terminal regions alter canonical ribosome dynamics, stochastically aborting translation”^47^. There were 3.9 EE repeats in the non-recovered MPNN sequences on average versus 1.6 in the recovered, and this feature achieves 0.82 ROC-AUC (Fig. 5E). Similarly, adenosine repeats in the nucleotide sequence have been shown to be potent translational disrupters^48,49^, which may correlate with the use of lysine (AAA, AAG) and glutamate (GAA) codons. The non-recovered sequences were significantly enriched in adenosine repeats in the DNA sequence, with means of 35.4 adenosine dinucleotide repeats in the non-recovered versus 21.7 in the recovered (Fig. 5F). The number of AA repeats was one of the most predictive features for sequence recovery, achieving 0.88 ROC-AUC, and also plausibly explaining why DNA sequence entropy alone had high predictive power at 0.90 ROC-AUC (Table SA1).

The most likely explanation for these design biases is that ProteinMPNN and SolubleMPNN preferentially predict K and E^50^, a preference which may be further compounded by the fact that RFdiffusion frequently creates alpha helix-rich backbones^51^. That said, sequences that had ≥2-fold increase in proliferation still had a mean of 18% of residues in K+E alpha helices (relative to the 35% seen in non-recovered sequences and to the 4% seen in the designs without significant change in proliferation (Fig. 5G)).

The sequences that exhibited CD20-specific depletion contained a large number of cysteines that were predicted to form disulfides within the binder chain (Figs. 5H, SA11). Given that tonic signaling can be enhanced through disulfide bond formation between hinge domain sulfhydryls^52-54^, and that inefficient folding (or, more likely, misfolding) may expose the hinge sulfhydryls or those in the binding domain, weak tonic signals might be greatly enhanced upon CD20 contact and subsequent disulfide bond formation.

Beyond these individual correlations, global confidence statistics from structure prediction models, such as ipTM and pLDDT, did not predict experimental outcomes (Table SA1). Likewise, ipSAE^55^, LIS score^56^, SAP score^57^, Rosetta InterfaceAnalyzer metrics^58^, PDockQ^59,60^, ProteinMPNN likelihoods, and ESM-2 pseudo log likelihood (PLL) also lacked substantial predictive power (Table SA1). For the latter two metrics, artifacts in the data led to deviation from expected scoring behavior. Likelihoods obtained from ProteinMPNN and SolubleMPNN on the Boltz-1-predicted structures were generally biased in favor of designs created using these models (Fig. SA15). ESM-2 PLL correlated with, and slightly underperformed in predictive power (Table SA1), amino acid sequence entropy for most sequences (Fig. SA16). This trend can be explained by the masked language modeling task becoming easier the less entropic the sequence is, which is further exemplified by the two outlier clusters defying this trend which contained large repeated regions that plausibly make the task of single residue infilling trivially easy with an in-context lookup, in agreement with existing literature^61^.

Overall, these findings exemplify how simple metrics can be more predictive of function than complex deep learning-based scores. Filtering out designs with ≥60% GC content and <1.9 DNA sequence entropy, ≥45 AA repeats in the DNA, ≥8 glutamate repeats, and ≥30% of residues in a K+E alpha helix results removes 4,600 sequences and increases the percent recovered after proliferation from 56.8% to 81.2% while increasing the percent of those that had CD20-specific proliferation from 5.9% to 7.6%. Further removing any sequences with cysteines raises the CD20-specific proliferation rate to 10.6%, though it reduces the percent recovered slightly to 72.6%. Notably, this filter removes two of the designs from the top 10 that lacked function entirely (1304, 1892) while preserving the rest. In contrast, filtering out sequences with <0.5 Boltz-1 ipTM would have removed two of the top 10 designs that were broadly functional and had binding affinity (designs 1506 and 5981) while only increasing the recovery rate by 0.7% and the CD20-specific proliferation rate by 0.3%. The predictive features discovered herein may help constrain future CAR-T engineering, both by AI-driven methods and otherwise.

## Discussion

The *Bits to Binders* competition was hosted to evaluate AI-driven protein design tools via a discovery-to-therapeutic pipeline. We collected 12,000 binder designs from 28 teams around the world that were created with a wide variety of approaches. Only 204 variants could not be cloned, in part due to repetitive sequences with high GC content, and the rest were tested in LEAH Labs’ high-throughput screen to evaluate a variety of phenotypes, starting with CD20-specific CAR-T proliferation. 4,985 of the designs (42%) dropped out of the population, indicating that some sequences or proteins interfered with cell viability. To verify that CD20-specific proliferation in the pooled growth assay translated to overall T cell function, we performed a series of functional assays on the ten best-performing unique designs, measuring specific cytotoxicity, cytokine release, proliferation, and expansion. We found that the majority of binders (7/10) outperformed the negative control across CD20-specific cytokine release, proliferation, and expansion, and 4/10 outperformed it on CD20-specific cytotoxicity. Two of the otherwise unsuccessful designs exhibited high non-specific killing. Ultimately, all of the designs underperformed the scFv positive control in all functional readouts except for cytokine production, in which design 5300 achieved more than 50% higher intracellular accumulation when cultured with CD20+ targets. As a caveat, while we established that some of the designs have broad T cell function, this was only performed in a single clinical isolate and the efficacy of these CARs in different donor lines is currently unknown.

Almost all of the designs that failed to be detected in the proliferation screen (98.9%) were created with ProteinMPNN or SolubleMPNN. We found that most of the failed MPNN designs preferentially contained highly adenosine-rich, lysine- and glutamate-containing alpha-helices, encompassing an average of 35% of residues versus only 15% in the recovered designs. While unnatural structures may pose problems on their own, we believe that many of the MPNN designs failed because of translational interference by glutamate and/or lysine^47,48,49^. To the extent that this hypothesis is correct, it provides a cautionary note that beyond the structure-centric task of binding, optimizing a protein’s amino acid sequence, and corresponding nucleotide sequence, is important for success, especially designing specifically for cellular expression. That said, there was clearly a sweet spot for design, as lower abundance K+E alpha-helices (18%) were moderately predictive of CD20-specific proliferation, in contrast to only 4% K+E in designs that did not show significant changes in proliferation. Designs associated with CD20-specific depletion, potentially indicative of exhaustion^62^, were highly enriched in cysteines. This validates the cautionary choice many model developers have made in explicitly avoiding cysteines in generated proteins.

Analyzing the top teams, we found some correlations between methodological choices and experimental outcome. BAGEL, Chroma, Bindcraft, and RFdiffusion all performed moderately well at CAR binder design. However, most of the sequences in this competition were created using RFdiffusion and ProteinMPNN, and this monoculture eventually skewed failure modes (K+E failures). Annealing structures with molecular dynamics prior to or after generation seemed to increase design success, whereas FastRelax and AmberRelax had no effect. While the top-performing teams employed iterative diffusion, this did not increase success across the board.

Overall, the *Bits to Binders* competition demonstrates that generative protein design has advanced to the point that it can directly be assessed by functional benchmarking. In particular, the identification of potential failure modes (GC dropouts, K+E abundance, excessive cysteines) can further guide CAR-T design, with a set of relatively simple filters almost doubling the rate of CD20-specific proliferating designs. By designing for a functional outcome, the results also begin to piece apart the complex interplay between expression, display, background (tonic) signaling, ligand-dependence, and proliferation. In particular, these results provide a counterpoint to the common perception that robust scFVs are the only viable starting points for CAR-T design, and raise the interesting prospect of initially canting design towards lower affinity spaces. The datasets and principles emerging from efforts like *Bits to Binders* should continue to serve as milestones for standardizing evaluation practices, guiding the development of more biologically informed models, and accelerating the application of AI-driven protein design for functional and ultimately clinical applications.

## Online Methods

### Selecting designs to test in each assay.

Teams were asked to submit 500 sequences in order of confidence of success. In total, 28 teams submitted designs. The first 466 sequences from each team were selected to accommodate 12,000 total designs, including 16 designs chosen by LEAH Labs. Some teams submitted less than 466 designs or submitted duplicates, resulting in fewer total sequences: Foldsmiths: 465, Furman lab: 433, Nucleate UK London: 414, Perforators: 343, Zist Rayanesh: 308, Amigo Acids: 187, and Virtue Therapeutics: 48. 32 of the submitted sequences contained “X” as one of the amino acids which was arbitrarily converted to “L” by Twist Bioscience.

The designs were sorted by -log10(FDR)*logFC, and the top ten non-redundant designs were chosen to proceed to the individual T cell functional assays. These designs came from the following teams: CAD Pro (1304), Perez Lab Gators (1506), LBM (1892), Nucleate UK London (2383), AIBI (3109), SNU LCDD (3494, 3718), Amigo Acids (5300), Binding Illini (5624), Schoeder Lab (5981). Sequence redundancy was determined by sharing at least 50% sequence identity (exact match).

For the binding affinity study, designs were pulled from the three CD20-specific proliferation categories: ≥2-fold increase, ≥2-fold decrease, and no significant change. Sequences from each category were clustered with MMseqs2. For the ≥2-fold decrease category, 10 sequences were chosen in order of ascending -log10(FDR)*logFC, skipping if a sequence from the same cluster was already chosen. For the no significant change category, a random sequence from 60 random clusters was chosen. For the ≥2-fold increase category, the 10 sequences that underwent individual functional assays were chosen, in addition to 20 sequences randomly chosen from the ≥2-fold increase category, ensuring that each sequence was chosen from a distinct cluster. In total, this amounted to 100 sequences along with the control scFv.

### Plasmid library construction.

The CAR construct began with a CD8α leader sequence, “stuffer” sequence (deoxyribose-phosphate aldolase) in the place of a binder via HiFi Assembly (New England Biolabs), a CD28 hinge and transmembrane domain, a CD28 co-stimulatory domain, and a CD3ζ signaling domain (modified from^63^). The F31S dihydrofolate reductase (DHFR) mutant was included after a P2A cleavage site, 3’ to the CAR construct, for further enrichment of the edited T cell population via selection with methotrexate (MTX)^64^. The stuffer sequence was removed while the Whitlow 218 linker was introduced via HiFi Assembly (New England Biolabs). This “binderless” construct was onboarded for oligo pool cloning of the 12,000 designed 80-amino-acid binders between the CD8α leader sequence and the 218 linker by Twist Bioscience.

### Pooled CAR-T generation and proliferation screen.

CAR-T cell libraries were generated with GeneWeld^38,39^, which enables precision integration of the CAR construct through homology mediated end joining by targeting the genome with CRISPR/Cas9 and the donor plasmid with a Universal gRNA to liberate the cargo and reveal 48 nucleotide homology arms. CAR variants were integrated near the N-terminus of the T cell receptor alpha chain, resulting in TRAC-negative, CAR-positive T cells.

Primary human T cells, previously isolated by BACS selection (Akadeum Life Science) from healthy donor leukopaks (Sanguine Biosciences) and batch cryopreserved in CryoStor CS10 (STEMCELL Technologies). For experiments, T cells were thawed and allowed to recover overnight in T cell media comprised of X-Vivo15 + 5% AB serum and, after a brief stimulation with DynaBeads at 3:1 bead to cell ratio, GeneWeld components were delivered via Nucleofection in P3 Buffer using the EO-115 program (Lonza). After a 72 hour recovery in T cell media containing 10 ng/mL each of IL-7 and IL-15, and CAR-T cells were transferred to GREX wells for an additional 7 days of expansion under methotrexate selection. After sampling gDNA for NGS of the “Input” sample, the cells were subject to repeated challenge with mitotically-inactivated (MI) - Raji-GFP target cells at an effector to target ratio of 1:1 in triplicate. Briefly, Raji-GFP cells were mitotically-inactivated with 10 ug/mL Mitomycin C (STEMCELL Technologies) with for 2 hours at a cell concentration of 1E6 cells/mL before washing three times and cryopreserving at 10-20E6 cells/mL in CryoStorCS10 for storage at −80°C. Running in parallel with MI-Raji-GFP co-cultures, the same CAR-T cell libraries were cultured alone in a GREX 6-well plate in triplicate to control for false-positives. After 2 weeks, a minimum of 12E6 cells were harvested per replicate for genomic DNA isolation, using Quick-DNA Miniprep Plus Kit from Zymo Research, and NGS analysis in triplicate for both conditions^65^. PCR was done with Herculase II Fusion DNA Polymerase from Agilent to add Nextera adaptors. PCR purification was done using the SPRIselect DNA Size Selection Reagent from Beckman Coulter. Samples were submitted to the University of Minnesota Genomics Center for further library preparation and sequencing using the Element AVITI24 sequencing platform.

Variants were counted, and those represented by fewer than 25 reads in any replicate were excluded from further analysis. For each construct, CD20-specific proliferation was defined as the ratio of normalized barcode abundance in the CD20-positive condition relative to the CD20-negative condition, CAR-T cells alone as a control for false-positive, non-specific expansion. Statistical significance was assessed using edgeR^66^, and false discovery rates (FDRs) were controlled at 5%.

### CAR-T cell immunophenotyping by flow cytometry.

Following methotrexate selection, CAR-T cells were characterized by multi-parameter flow cytometry. 1E5 cells were harvested into a round-bottom 96-well plate and washed with ice-cold MACS Buffer (PBS supplemented with 0.5% BSA and 2 mM EDTA). Surface antigen staining was performed by incubating cells with a fluorophore-conjugated antibody cocktail targeting the CAR (anti-218L-AlexaFluor 488, Cell Signaling Technologies) and the TCR (anti-TCRa/b-APC, Miltenyi Biotec), each at a 1:50 dilution in MACS buffer for 10 minutes at 4°C in the dark. To assess viability and exclude non-viable cells from analysis, cells were washed with MACS buffer and stained with eBioscience Fixable Viability Dye eFluor 450 (Invitrogen) at a 1:500 dilution for 10 minutes at 4°C. After two final washes, cells were resuspended in MACS buffer and acquired on a MACSQuant Analyzer 16 flow cytometer (Miltenyi Biotec). Data were analyzed using FlowJo software (v10) with the following hierarchical gating strategy: 1) Identification of lymphocytes based on forward scatter (FSC-A) and side scatter (SSC-A) to exclude debris, followed by 2) doublet discrimination via FSC-A vs FSC-H to ensure single-cell analysis, and 3) gating on the viability dye-negative population to exclude dead cells, allowing 4) CAR and TCR subset and expression analysis within the live cell population. Controls included Mock-transfected T cells (TCR+CAR−) and unstained (FMO) samples that did not receive anti-CAR (218L) or anti-TCR antibodies.

### Specific lysis individual T cell functional assay.

CD20+ target cells -specific cytotoxicity was evaluated after co-incubating 25,000 CAR-T cells per well with CD20-positive target cells (Raji-GFP) and CD20-negative controls (K562-GFP) at varying effector-to-target ratios for 48 hours. A MACSQuant Analyzer 16 flow cytometer (Miltenyi Biotec) was used to quantify specific lysis by counting the number of GFP+ target cells remaining per well when co-cultured with T cells, relative to wells containing targets cells alone as no lysis controls. Specific Lysis = (number of GFP+ targets remaining / target cells alone) x 100%. Multiple effector-to-target ratios allowed for AUC analysis to compare specific lysis of CD20+ targets (Raji^AUC^-K562^AUC^) between individual CAR-T clones of interest and control T cells.

### Cytokine generation individual T cell functional assay.

T cells were cultured alone or co-cultured with Raji-GFP target cells at a 1:1 E:T ratio for 16 hours before adding Brefeldin A (BioTechne) for an additional 4-5 hours. After activation, cells were harvested, washed and stained with eBioscience Fixable Viability Dye eFluor 450 (Invitrogen) at a 1:500 dilution for 10 minutes at 4°C to exclude non-viable cells from analysis before fixation, permeabilization, and intracellular cytokine staining using CytoFix/CytoPerm kit (BioTechne). Specifically, anti-IL2-PE and anti-IFNg-VioR667 REA antibodies, along with fluorochrome conjugated REA isotype controls (1:50 dilution, Miltenyi Biotec), were utilized to enumerate cytokine producing T cells with a MACSQuant Analyzer 16 flow cytometer (Miltenyi Biotec). Fluorochrome-conjugated REA Intracellular isotype antibodies were used as controls to assess the percentages of T cells producing either IL2, IFNγ, or both were added together to reflect total frequency of cytokine producing T cells.

### Expansion and proliferation individual T cell functional assays.

CAR-T cell division and cumulative expansion were tracked over 5 days after stimulation with CD20-positive targets at varying effector-to-target ratios. Expansion was quantified as total viable CAR-T yield 5 days after stimulation using volumetric sampling for accurate cell enumeration with the MACSQuant Analyzer 16 flow cytometer. T cell proliferation over 5 days was simultaneously inferred from cell division rates measured by the change in the mean fluorescence intensity (MFI) of Cell Trace Violet (Thermo Fisher Scientific) in T cells labeled according to the manufacturer’s instructions. Multiple effector-to-target (E:T) ratios along with no MI-Raji target controls allowed for AUC analysis to compare expansion and proliferation between individual CAR-T clones of interest and control T cells.

### Cell-free expression of binder variants for binding affinity.

DNA constructs encoding the binder variants were designed by reverse-translating the protein sequences and optimizing them for manufacturability and yield using codon optimization algorithms. Each construct was fused to a C-terminal GFP11-TwinStrep (TST) tag to enable split-GFP-based expression quantification and affinity capture for kinetic characterization. Optimized variant sequences and the 3′ fragment encoding linker and affinity tags were ordered as Gene Fragments (Twist Bioscience). Constructs were assembled using the NEBuilder HiFi DNA Assembly Kit (NEB) in 2 μL reactions. Assembly quality was verified by capillary electrophoresis (Agilent ZAG DNA Analyzer), and DNA concentrations were quantified using the Qubit DNA Quantification Kit (Invitrogen).

Protein expression was performed in 8 μL reactions using an optimized prokaryotic cell-free protein synthesis (CFPS) system with 4 nM assembled DNA template. Reactions were incubated at 37 °C for 8–12 hours. Soluble expression was confirmed using a split-GFP complementation assay, and protein yields were normalized using an affinity-based quantification assay. Crude lysates were used directly for downstream kinetic measurements.

### Surface Plasmon Resonance (SPR) Affinity Characterization

Binding kinetics were characterized using SPR on a Carterra LSA XT high-throughput platform. A carboxymethylated chip surface was functionalized with Strep-Tactin XT (IBA Lifesciences) using EDC/NHS chemistry. TwinStrep-tagged binder variants (from crude CFPS lysates diluted 125-fold) were captured onto the surface using the 96-channel array head (750 s capture, 600 s baseline).

The target antigen, full-length human CD20 (Acro Biosystems, CD0-H52D5; Flag/His-tagged, detergent-solubilized), was reconstituted in running buffer (50 Mm HEPES, 150 mM NaCl, DDM + CHS, pH 7.5) and injected in solution over the immobilized variants. CD20 was tested at five half-log serial dilutions starting from 1000 nM. Measurements were performed in triplicate using multi-cycle kinetics, consisting of 60 s baseline, 300 s association, and 600 s dissociation. Surfaces were regenerated with 10 mM glycine-HCl (pH 1.5) between full concentration series.

### Bio-Layer Interferometry (BLI)

A subset of variants was additionally tested by BLI on a Gator Pro system using the same CD20 preparation and detergent-containing buffer (50 mM HEPES, 100 mM NaCl, DDM + CHS). Binder variants were immobilized, and CD20 was run in solution as analyte. Assay steps consisted of 60 s baseline, 120 s loading, 120 s baseline, 60 s association, and 300 s dissociation.

### Data Processing and Kinetic Analysis

Sensorgrams were reference- and baseline-corrected and globally fitted to a 1:1 Langmuir binding model using Adaptyv Fitting software when signal quality permitted. Kinetic parameters (*KD, kon, koff*) were extracted from global fits across concentrations. When global fitting was not feasible, alternative models were applied and parameters were selected based on fit quality.

Variants were classified as binders based on quantifiable kinetic fits. In cases where fitting was not reliable but association-phase signal exceeded 300% of the negative control, binding was assigned based on signal magnitude and estimated using Anabel^43^.

### Computational feature generation and statistical modeling.

For each submitted binder, we computed a panel of >400 features from the nucleotide and amino acid sequences (Supplementary Information B). Sequence-derived metrics included nucleotide and amino acid composition (for example, GC content), k-mer and repeat statistics (including homopolymers and dipeptide repeats), and Shannon entropy at the DNA and protein levels to quantify sequence complexity. Structure-informed features were derived from Boltz-1, ESMFold, and Chai-1 predictions of binder–CD20 complexes. These included model confidence statistics (e.g., pLDDT and ipTM), global and per-residue structural descriptors (e.g., secondary-structure composition and predicted disulfide content), and interface-focused metrics computed on the predicted complexes (e.g., contact counts and interface energy or interaction scores).

Features were analyzed against four outcomes: detection in the Twist plasmid pool quality report, recovery by NGS after the pooled proliferation assay, and CD20-specific change in proliferation. For binary outcomes, we trained logistic regression models using stratified 5-fold cross validation and evaluated performance with ROC-AUC. To mitigate method-specific confounding, analyses were repeated on restricted subsets when appropriate (e.g., only ProteinMPNN/SolubleMPNN-generated designs). Feature relevance was assessed by comparing cross-validated performance across individual features and by inspecting class-conditional feature distributions.

## Supplementary Material

Supplement 1

## Data Availability

All data associated with this study is publicly available on Zenodo at doi.org/10.5281/zenodo.18840101. Summarized data used for the analysis can also be found at github.com/kosonocky/bits-to-binders.

## Code Availability

Code used in the competition analysis is publicly available on Zenodo at doi.org/10.5281/zenodo.18840101 and at github.com/kosonocky/bits-to-binders. Code used by the competitors is listed in Supplementary Information E, when available. Inquiries on the competitors’ code should be directed to the contacts listed therein.

## List of *Bits to Binders* Competitors

Daniel Acosta, Stefano Angioletti-Uberti, Vinayak Annapure, Neil Anthony, Rana A. Barghout, Max Beining, Parth Bibekar, Dionessa Biton, Patrick Bryant, Anton Bushuiev, Cianna Calia, Ava Chan, Jie Chen, Akshay Chenna, Nicole Chiang, Vivian Chu, Joseph Clark, Alia Clark-ElSayed, Elliot Cole, Qian Cong, Shreyasi Das, Tanner Dean, Tyler Derr, Alejandro Diaz, Jesse Durham, Moritz Ertelt, Antonio Fonseca, Ora Furman, Tynan Gardner, Jokent Gaza, Nathaniel Greenwood, Rui Guo, Abel Gurung, Vivian Haas, Seyed Alireza Hashemi, Dieter Hoffmann, Sofiia Hoian, Corey Howe, Calvin XiaoYang Hu, Jinling Huang, Nader Ibrahim, Satoshi Ishida, Ansar Ahmad Javed, Aswini Javvadi, Roman Jerala, Daksh Joshi, Even Kiely, Haelyn Kim, Diego Kleiman, Sarah Knapp, Huan Koh, Petr Kouba, Yong Youn Kwon, Duško Lainšček, Jakub Lála, Hakyung Lee, Qiuzhen Li, Max Lingner, Yuxuan Liu, Yunchao Liu, Qianchen Liu, Ajasja Ljubetič, Marko Ludaic, Karl Lundquist, Ashish Makani, Javier Marchena-Hurtado, Freddie Martin, Jens Meiler, Fernando Meireles, David Miller, Nuria Mitjavila, Tjaša Mlakar, Rajarshi Mondal, Saeideh Moradvandi, Rocco Moretti, Sai Jaideep Reddy Mure, Pragati Naikare, Fatemeh Nasiri, Kaveh Nasrollahi, Yisel Martinez Noa, Joseph Openy, Aoi Otani, Vikrant Parmar, Jimin Pei, Alberto Perez, Rajat Punia, Francisco Requena, Dominic Rieger, Aparna Sahu, Elias Sanchez, Rohit Satija, Tadej Satler, Tom Schlegel, Yang Shen, Diwakar Shukla, Rangana De Silva, Benedikt Singer, Arjun Singh, Amardeep Singh, Bhumika Singh, Jinwoong Song, Harish Srinivasan, Hannah Stewart, Zhaoqian Su, Julia Varga, Xin Wang, Dion Whitehead, Matthew Williams, Max Witwer, Rujie Yin, Song Yin, Rongqing Yuan, Nikola Zadorozhny, Dishant Parag Zaveri, Genhui Zheng, Yizhen Zheng, Jian Zhou, Shaowen Zhu, Lin Zhu
