# Supplementary material for: Validation and analysis of 12,000 AI-driven CAR-T designs in the Bits to Binders competitions
Source: bioRxiv. 2026 Mar 4:2026.03.03.709355. Preprint. [Version 2] doi: 10.64898/2026.03.03.709355 (PMC13001334; doi:10.64898/2026.03.03.709355)
Supplement: Supplement 1 [file NIHPP2026.03.03.709355v2-supplement-1.pdf]

bioRxiv preprint doi: https://doi.org/10.64898/2026.03.03.709355; this version posted March 4, 2026. The copyright holder for this preprint (which was not certified by peer review) is the author/funder, who has granted bioRxiv a license to display the preprint in perpetuity. It is made available under aCC-BY 4.0 International license.

Supplemental Information

A. Supplemental Figures and Tables

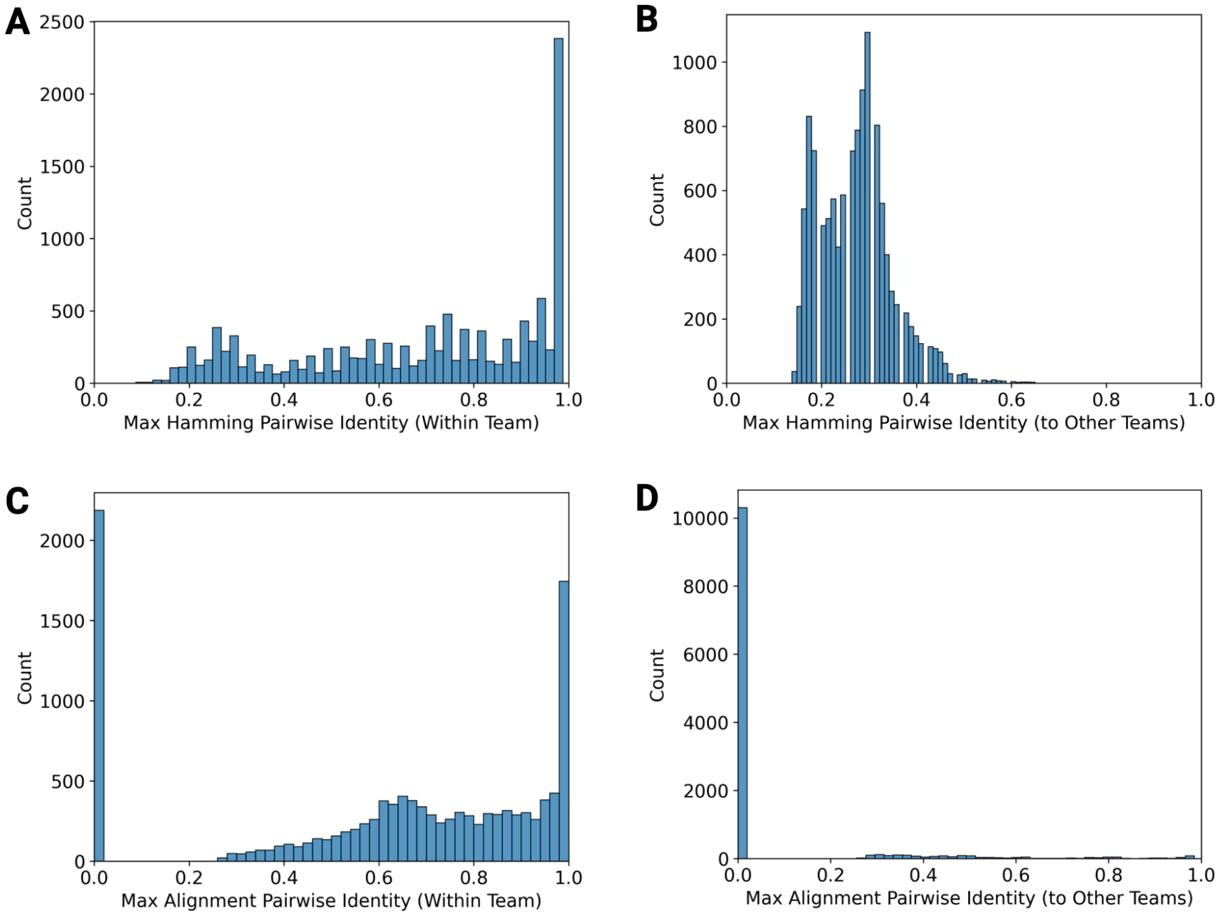

Figure SA1. Maximum pair-wise sequence identities of the designed binders. The following sequence identity metrics were computed: **(A)** Hamming similarity (full sequence, position-by-position, ungapped) between each sequence and the other sequences from the same team, **(B)** Hamming similarity between each sequence and all sequences from the other teams, **(C)** Local alignment-based sequence identity (MMseqs2[37], -s 7.5, --cov-mode 0, qcov & tcov ≥ 0.3) between each sequence and all sequences from the same team, **(D)** Local alignment-based sequence identity between each sequence and all sequences from the other teams. Insignificant alignments are set to zero pairwise identity. Although there was high within-team redundancy, the designs from a given team were largely unique relative to those from other teams. The exceptions to this were teams that included significant portions of known CD20 antibodies.

bioRxiv preprint doi: https://doi.org/10.64898/2026.03.03.709355; this version posted March 4, 2026. The copyright holder for this preprint (which was not certified by peer review) is the author/funder, who has granted bioRxiv a license to display the preprint in perpetuity. It is made available under aCC-BY 4.0 International license.

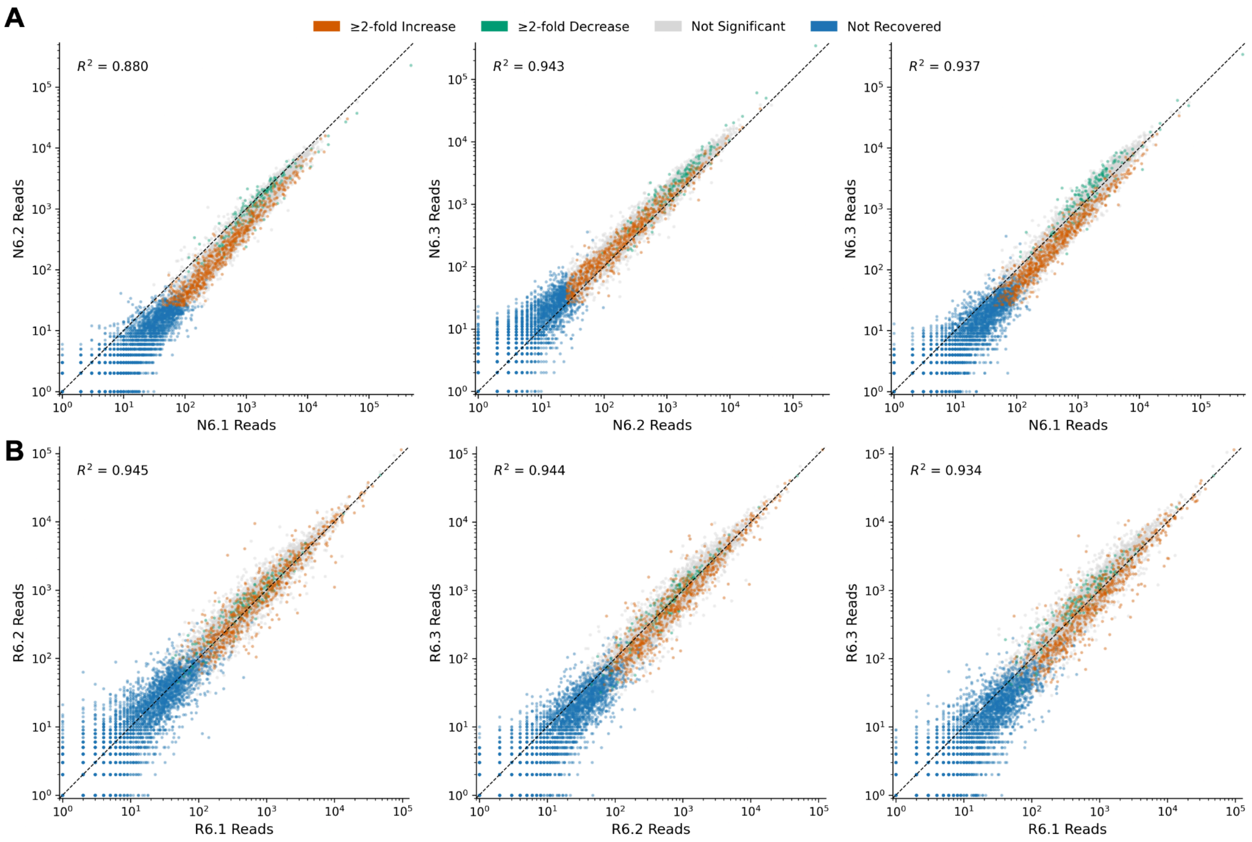

Figure SA2. NGS reads demonstrate minimal jackpot effects between replicates in pooled proliferation assay. (A) Reads were generally correlated between the three non-CD20 replicates (N6) and the **(B)** three CD20+ co-culture replicates (R6), indicating that there was minimal jackpot effect occurring.

bioRxiv preprint doi: https://doi.org/10.64898/2026.03.03.709355; this version posted March 4, 2026. The copyright holder for this preprint (which was not certified by peer review) is the author/funder, who has granted bioRxiv a license to display the preprint in perpetuity. It is made available under aCC-BY 4.0 International license.

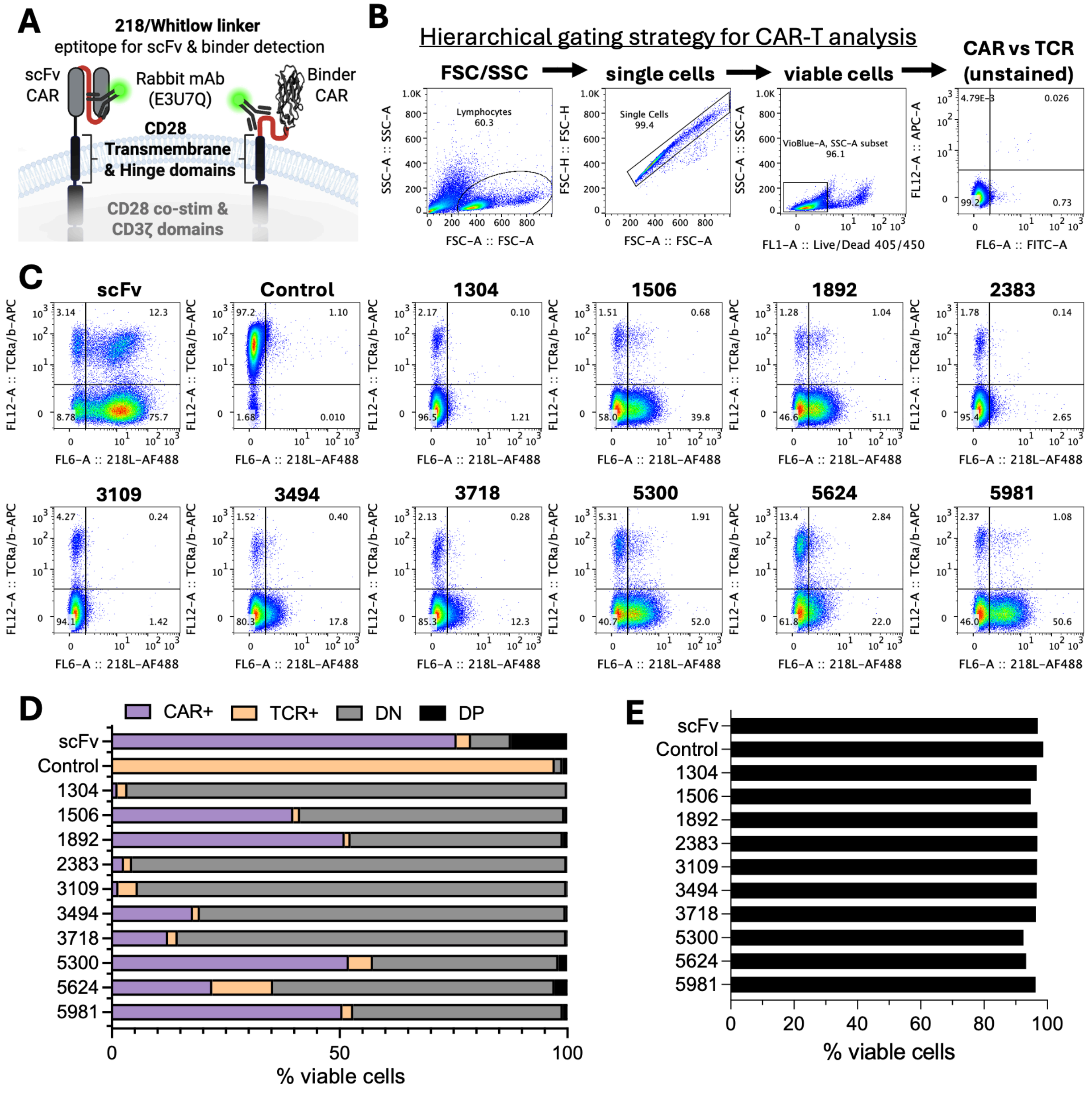

Supplemental Figure SA3: CAR and TCR expression determined by flow cytometry. (A) 218/Whitlow linker present in the scFv was designed into the binder construct as an antibody epitope. **(B)** Hierarchical gating strategy for viable CAR-T enumeration, as shown in **(C)** scatter plots depicting CAR and TCR surface expression for controls and binder variant CAR-T cells. **(D)** graphical representation of flow cytometry data showing T cell populations expressing CAR, TCR, neither, or both. **(E)** Viability assessment of controls and binder variant CAR-T cells.

bioRxiv preprint doi: https://doi.org/10.64898/2026.03.03.709355; this version posted March 4, 2026. The copyright holder for this preprint (which was not certified by peer review) is the author/funder, who has granted bioRxiv a license to display the preprint in perpetuity. It is made available under aCC-BY 4.0 International license.

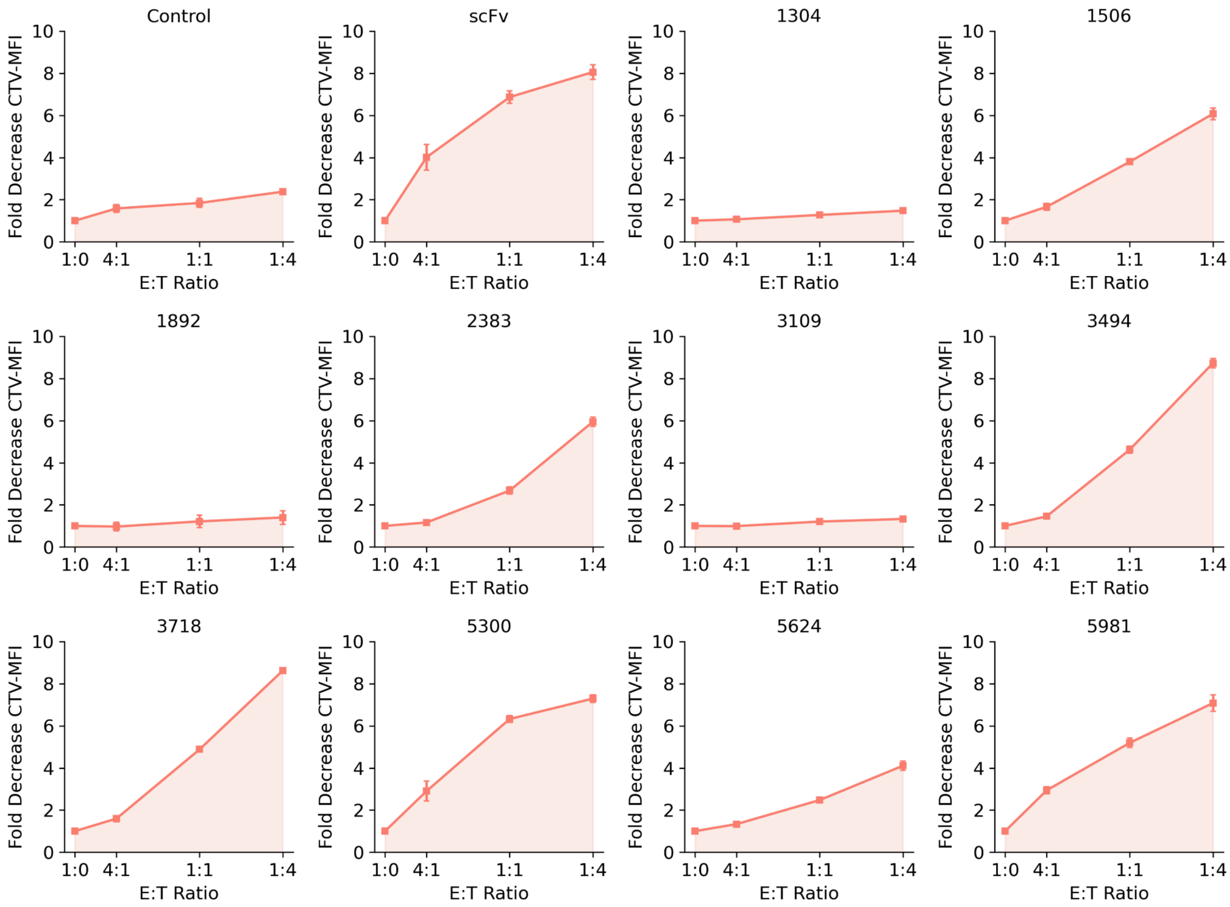

Figure SA4. Proliferation via decrease in CTV-MFI. Proliferation was measured over 5 days following stimulation with Raji (CD20+) targets across multiple E:T ratios by decrease in median fluorescence intensity (MFI) of the Cell Trace Violet (CTV) signal.

bioRxiv preprint doi: https://doi.org/10.64898/2026.03.03.709355; this version posted March 4, 2026. The copyright holder for this preprint (which was not certified by peer review) is the author/funder, who has granted bioRxiv a license to display the preprint in perpetuity. It is made available under aCC-BY 4.0 International license.

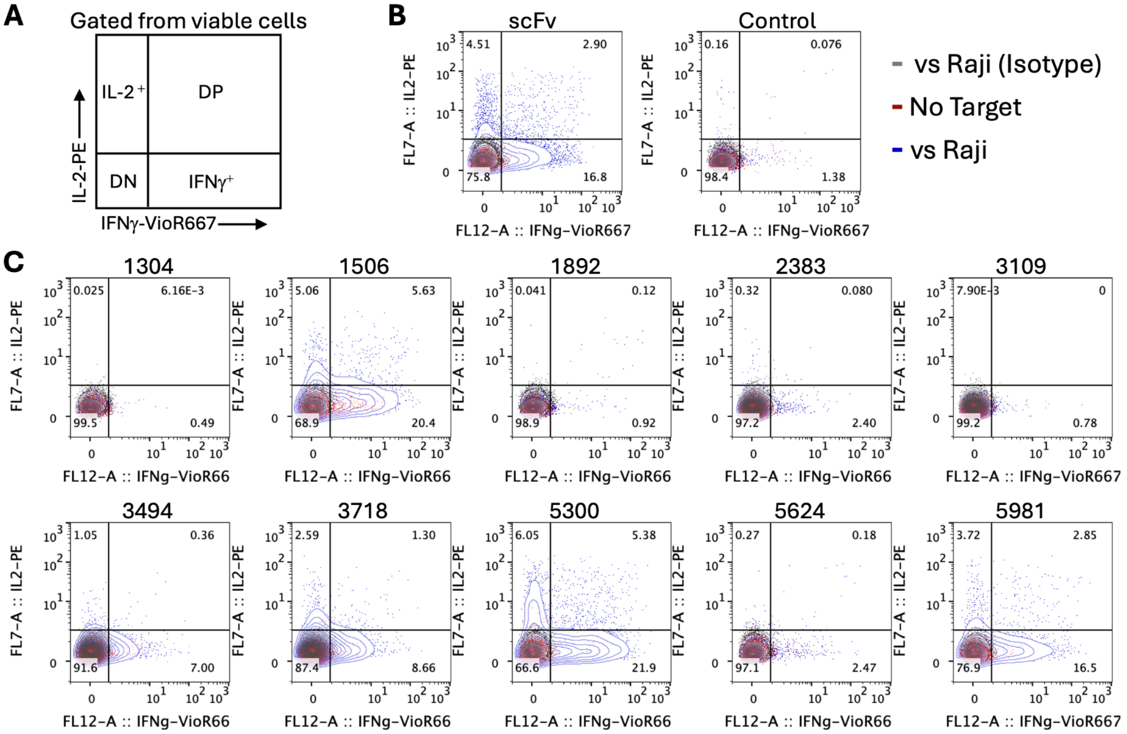

Figure SA5. Flow cytometry data of T cell proliferation with CellTrace Violet (CTV) dilution. CTV (x-axis) peaks analyzed after 5 days in co-culture with MI-Raji target cells at various Effector-to-Target (E:T) ratios.

bioRxiv preprint doi: https://doi.org/10.64898/2026.03.03.709355; this version posted March 4, 2026. The copyright holder for this preprint (which was not certified by peer review) is the author/funder, who has granted bioRxiv a license to display the preprint in perpetuity. It is made available under aCC-BY 4.0 International license.

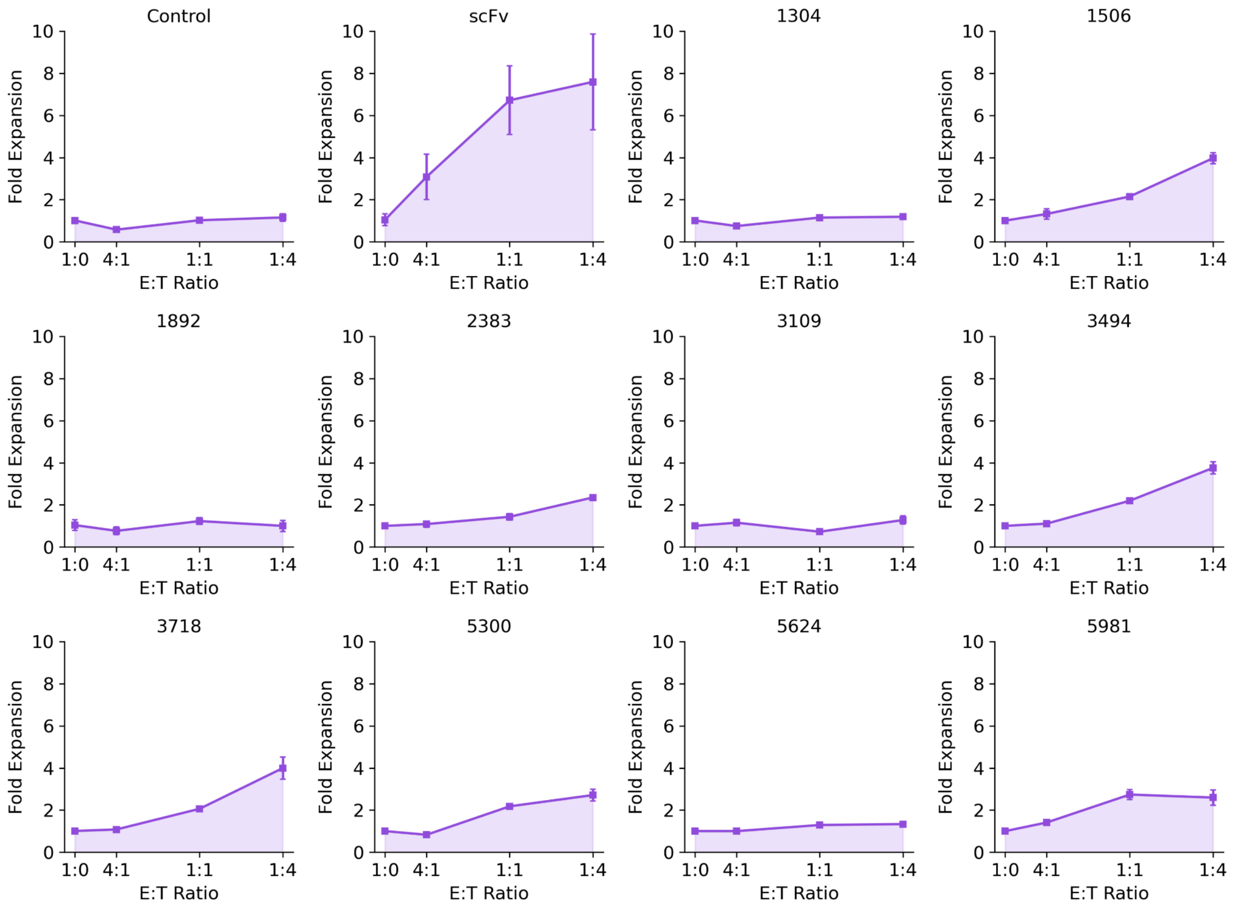

Figure SA6. Fold expansion by cell counts. Total CAR-T expansion was measured over 5 days following stimulation with Raji (CD20+) targets across multiple E:T ratios by total viable CAR-T yield using volumetric flow cytometry.

bioRxiv preprint doi: https://doi.org/10.64898/2026.03.03.709355; this version posted March 4, 2026. The copyright holder for this preprint (which was not certified by peer review) is the author/funder, who has granted bioRxiv a license to display the preprint in perpetuity. It is made available under aCC-BY 4.0 International license.

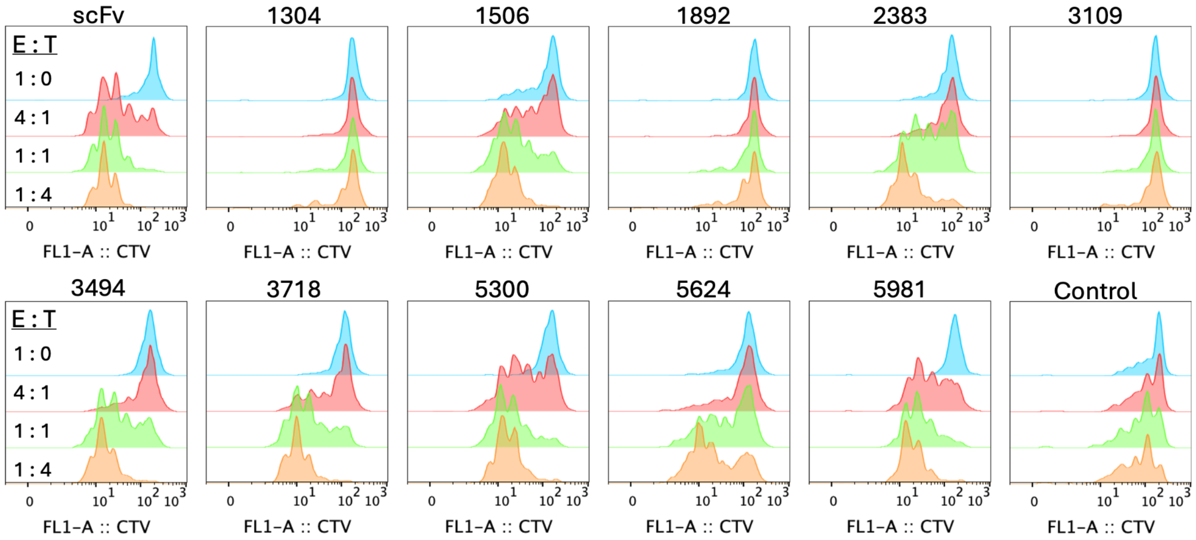

Fig SA7. CAR-T cell cytokine generation induced by CD20+ Raji target cells. (A) Gating strategy illustrating cytokine-generating populations as analyzed by flow cytometry. **(B)** CD20 scFv CAR and Control (unedited, TCR+CAR-neg T cells), were stained with fluorochrome-conjugated antibodies after incubating alone (red) or in co-culture with Raji target cells. Specifically, anti-IFNg and anti-IL2 antibodies, or the requisite isotype antibody controls, were used to analyze both controls and **(C)** individual CD20 binders as CAR-T cells.

bioRxiv preprint doi: https://doi.org/10.64898/2026.03.03.709355; this version posted March 4, 2026. The copyright holder for this preprint (which was not certified by peer review) is the author/funder, who has granted bioRxiv a license to display the preprint in perpetuity. It is made available under aCC-BY 4.0 International license.

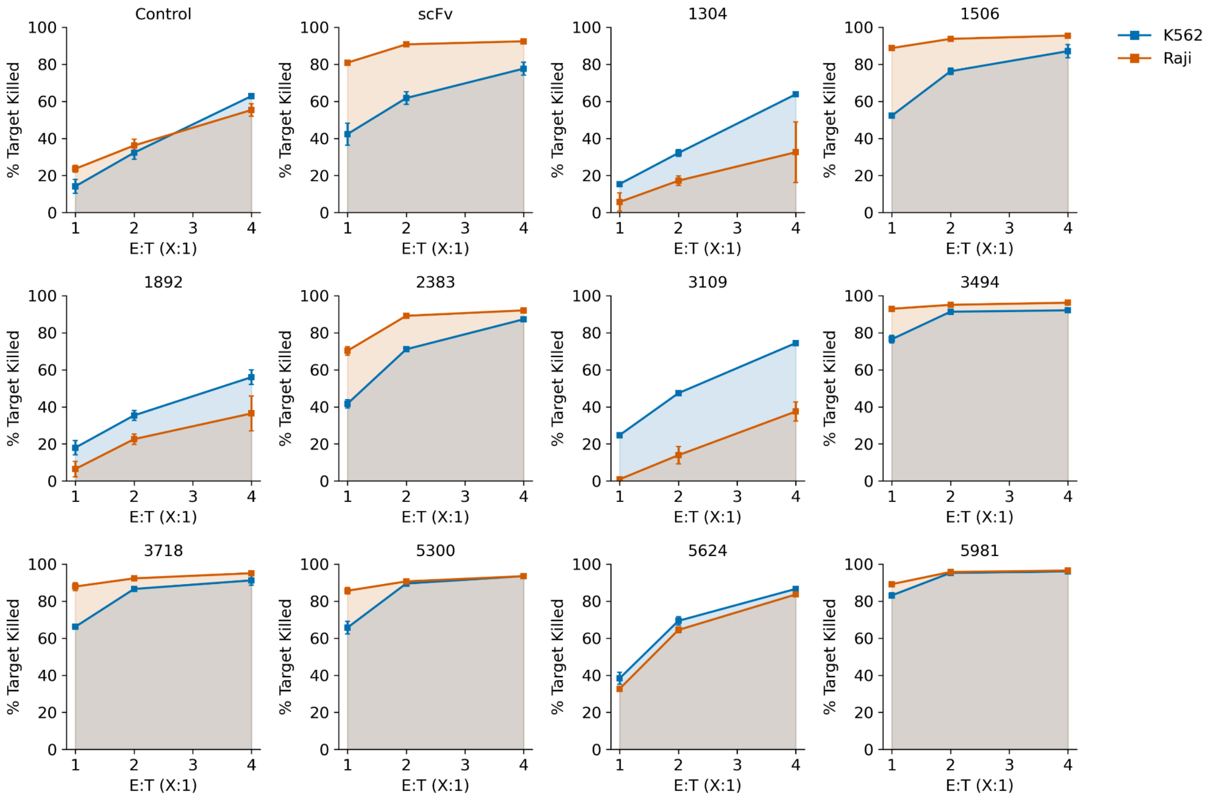

Figure SA8. Raji-specific cytotoxicity vs K562 control. GFP assay readouts across multiple E:T ratios for Raji (CD20+) and K562 (CD20-) targets, overlaid for direct comparison.

bioRxiv preprint doi: https://doi.org/10.64898/2026.03.03.709355; this version posted March 4, 2026. The copyright holder for this preprint (which was not certified by peer review) is the author/funder, who has granted bioRxiv a license to display the preprint in perpetuity. It is made available under aCC-BY 4.0 International license.

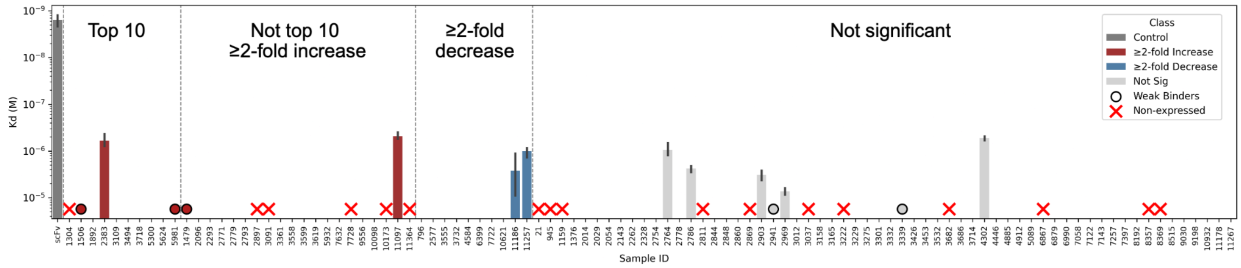

Figure SA9. Binding affinities to detergent-solubilized CD20 for 100 binders expressed as 80-amino acid constructs by *in vitro* transcription/translation. Along with the positive control scFv, 100 binders were submitted for binding characterization via SPR and BLI spanning the three proliferation categories in triplicate. These include the top ten from the high-throughput proliferation screen, 20 additional designs showing CD20-specific ≥2-fold increase, 10 designs showing CD20-specific ≥2-fold decrease, and 60 that had no significant change. Those that demonstrated binding, but were not quantifiable in the experimentally tested concentrations, are denoted with a filled circle colored by its class. Those that were not able to be expressed as isolated 80-mer constructs by *in vitro* transcription/translation are marked with red X's. Absence of a bar indicates successful expression but no observed binding.

bioRxiv preprint doi: https://doi.org/10.64898/2026.03.03.709355; this version posted March 4, 2026. The copyright holder for this preprint (which was not certified by peer review) is the author/funder, who has granted bioRxiv a license to display the preprint in perpetuity. It is made available under aCC-BY 4.0 International license.

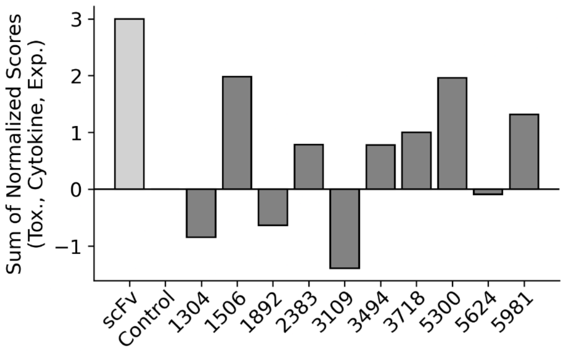

Figure SA10. Rankings used to determine competition winners. To get an overall assessment on the functional performance of each selected design, the final rankings in the competition were determined by using cytotoxicity, cytokine, and expansion outcomes min/max normalized to the negative and positive control, respectively. These were chosen to reduce bias away from growth, which would have occurred had both proliferation and expansion been used in the final score.

bioRxiv preprint doi: https://doi.org/10.64898/2026.03.03.709355; this version posted March 4, 2026. The copyright holder for this preprint (which was not certified by peer review) is the author/funder, who has granted bioRxiv a license to display the preprint in perpetuity. It is made available under aCC-BY 4.0 International license.

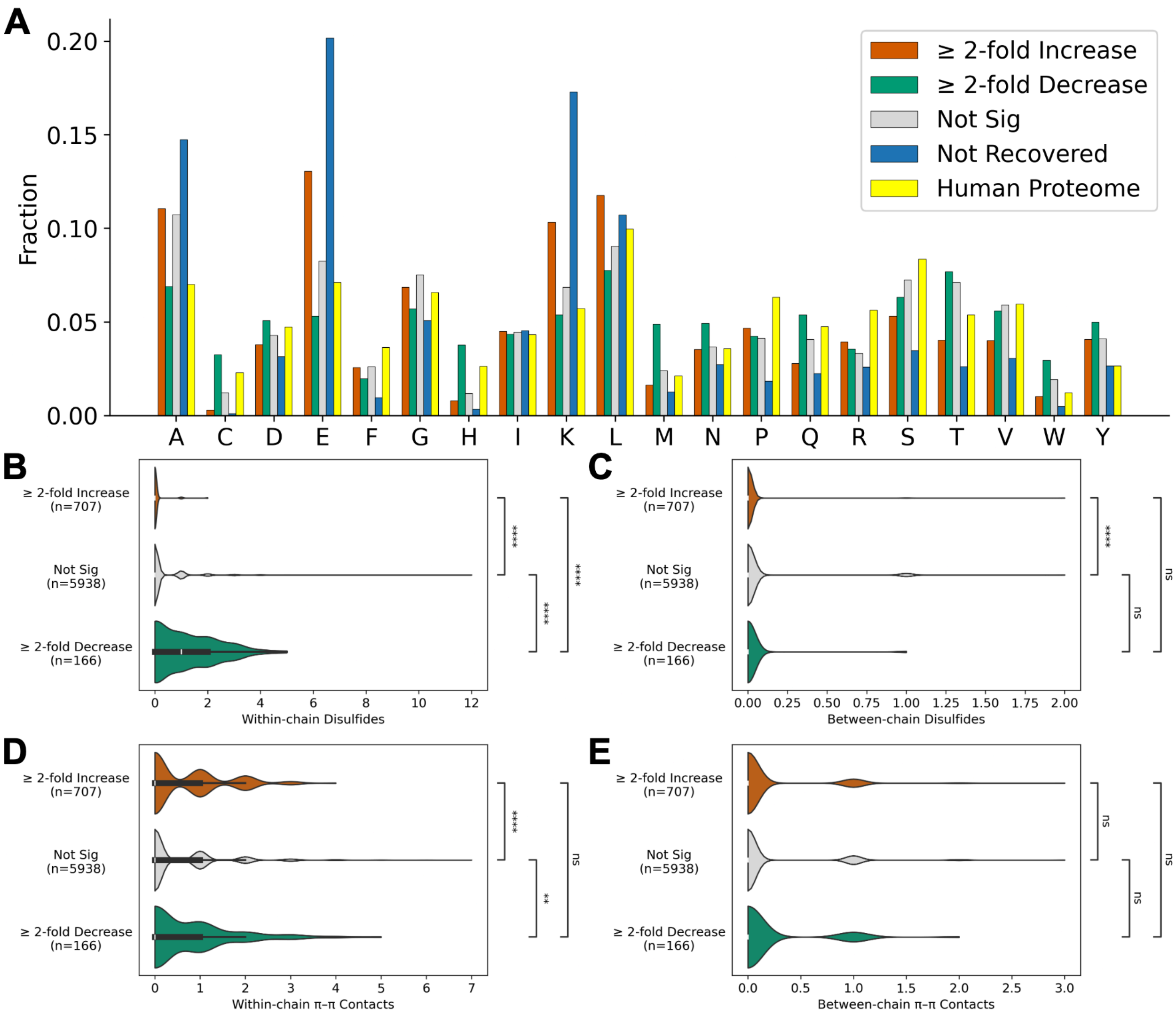

Figure SA11. Amino acid compositional differences and predicted contacts. (A) Composition of the four categories obtained from the proliferation screen. Compared to the human proteome, the ≥2-fold increase category is enriched in A, E, K, and L and diminished in C and H, whereas the ≥2-fold decrease category was enriched in C, H, M, and W. **(B)** The ≥2-fold decrease category is enriched in predicted disulfide bonds within the binder chain on Boltz-1 predicted structures. **(C)** There is no trend for disulfides between the binder and CD20. **(D)** There is no trend for pi-pi contacts within the binder chain. **(E)** There is no trend for pi-pi contacts between the binder and CD20. Significance from two-sided BH-corrected Mann-Whitney-Wilcoxon tests; ns, not significant; *, p ≤ 0.05; **, p ≤ 0.01; ***, p ≤ 0.001; ****, p ≤ 0.0001.

bioRxiv preprint doi: https://doi.org/10.64898/2026.03.03.709355; this version posted March 4, 2026. The copyright holder for this preprint (which was not certified by peer review) is the author/funder, who has granted bioRxiv a license to display the preprint in perpetuity. It is made available under aCC-BY 4.0 International license.

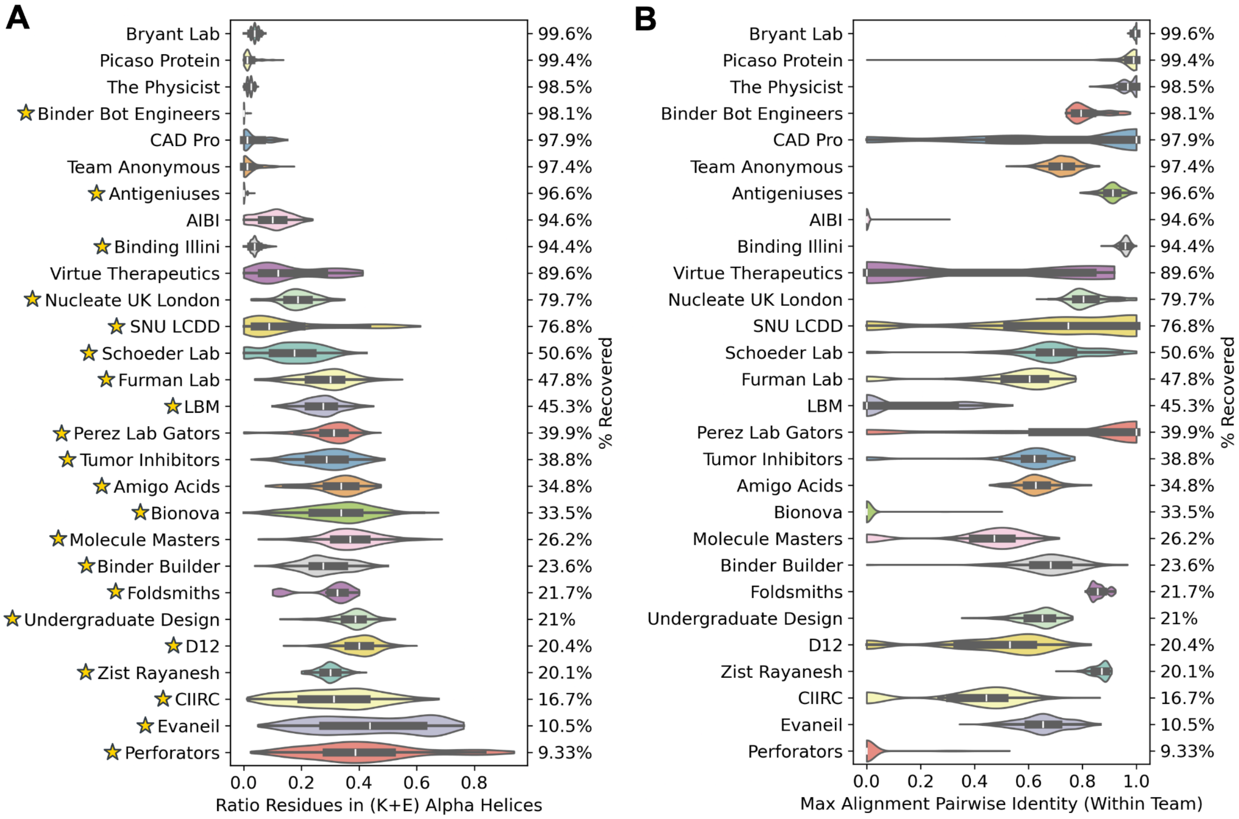

Figure SA12. Team-wise breakdown of sequence recovery. (A) Sequence recovery by ratio of residues in K+E alpha helices. Teams that used ProteinMPNN or SolubleMPNN are denoted by stars. **(B)** Sequence recovery by sequence diversity calculated by the maximum MMseqs2 alignment pairwise similarity to a sequence from the same team (higher = more redundant; lower = more unique). Several of the teams with high sequence recoveries have redundant sequences, differing by only a few residues. However, several teams achieved high sequence diversity with substantial diversity between designs.

bioRxiv preprint doi: https://doi.org/10.64898/2026.03.03.709355; this version posted March 4, 2026. The copyright holder for this preprint (which was not certified by peer review) is the author/funder, who has granted bioRxiv a license to display the preprint in perpetuity. It is made available under aCC-BY 4.0 International license.

Table SA1. Best features for predicting experimental readouts. For each of four experimental outcomes, selected high-performing features are shown. Each feature was used to train a logistic regression model (max_iter=1000, 5-fold random split) to predict the given class. Similar to above, negative samples were filtered out at each step. Set sizes are as follows: DNA detected: 12K total; LEAH Recovered: 11796 total (removed DNA not detected); ≥2-fold increase and ≥2-fold decrease: 6811 (removed both DNA not detected and LEAH not recovered). Values are ROC-AUC scores across the 5 splits, sorted by ≥2-fold increase.

Metric	DNA detected	LEAH recovered	≥2-fold increase	≥2-fold decrease
ratio_KE	0.617	0.896	**0.725**	0.620
dssp_ke_alpha_ratio	0.638	**0.905**	0.700	0.648
num_AA_repeats_dna	0.588	0.882	0.681	0.567
num_EE_repeats_aa	0.625	0.818	0.670	0.596
num_cysteines	0.561	0.683	0.663	0.738
dna_sequence_entropy	0.790	0.897	0.660	0.658
aa_sequence_entropy	0.795	0.833	0.626	**0.757**
boltz_autoregwithseq_proteinmpnn	0.536	0.665	0.630	0.729
esm2_binder_LL_car_target	0.763	0.834	0.597	0.710
seq_kl_vs_human	**0.803**	0.828	0.578	0.618
boltz_A_BC_max_ipSAE	0.555	0.517	0.519	0.489
dna_gc_content	0.568	0.625	0.519	0.645
boltz_rosetta_complex_normalized	0.493	0.570	0.499	0.463
boltz_iptm	0.555	0.525	0.505	0.457
boltz_iplddt	0.570	0.662	0.570	0.516
boltz_A_BC_max_lis_score	0.533	0.532	0.515	0.503
boltz_sap_score	0.697	0.791	0.548	0.558
boltz_A_BC_max_pdockq	0.546	0.636	0.553	0.629

bioRxiv preprint doi: https://doi.org/10.64898/2026.03.03.709355; this version posted March 4, 2026. The copyright holder for this preprint (which was not certified by peer review) is the author/funder, who has granted bioRxiv a license to display the preprint in perpetuity. It is made available under aCC-BY 4.0 International license.

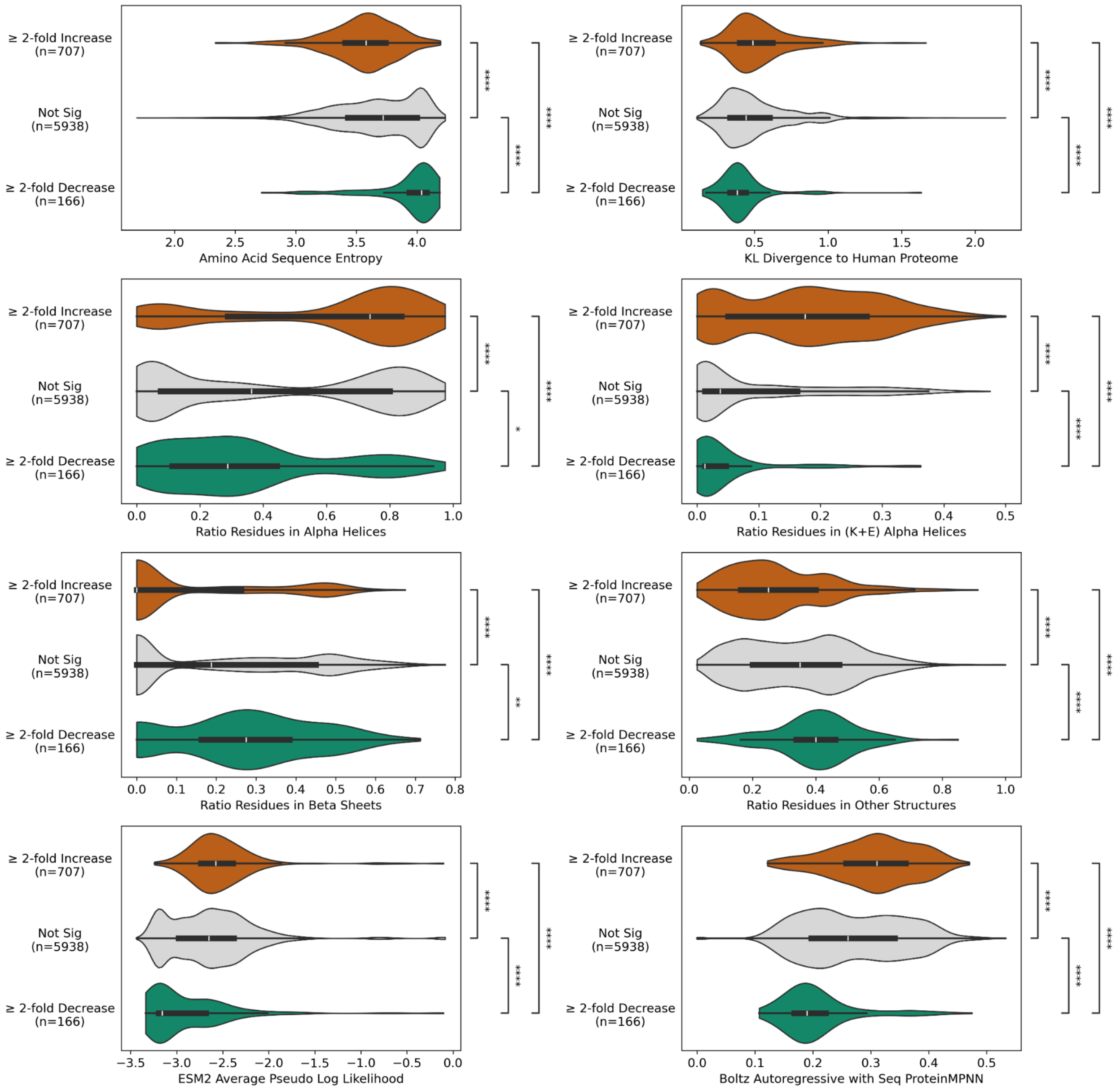

Figure SA13. Additional differences between proliferation outcomes. Significance from two-sided BH-corrected Mann-Whitney-Wilcoxon tests; ns, not significant; *, p ≤ 0.05; **, p ≤ 0.01; ***, p ≤ 0.001; ****, p ≤ 0.0001.

bioRxiv preprint doi: https://doi.org/10.64898/2026.03.03.709355; this version posted March 4, 2026. The copyright holder for this preprint (which was not certified by peer review) is the author/funder, who has granted bioRxiv a license to display the preprint in perpetuity. It is made available under aCC-BY 4.0 International license.

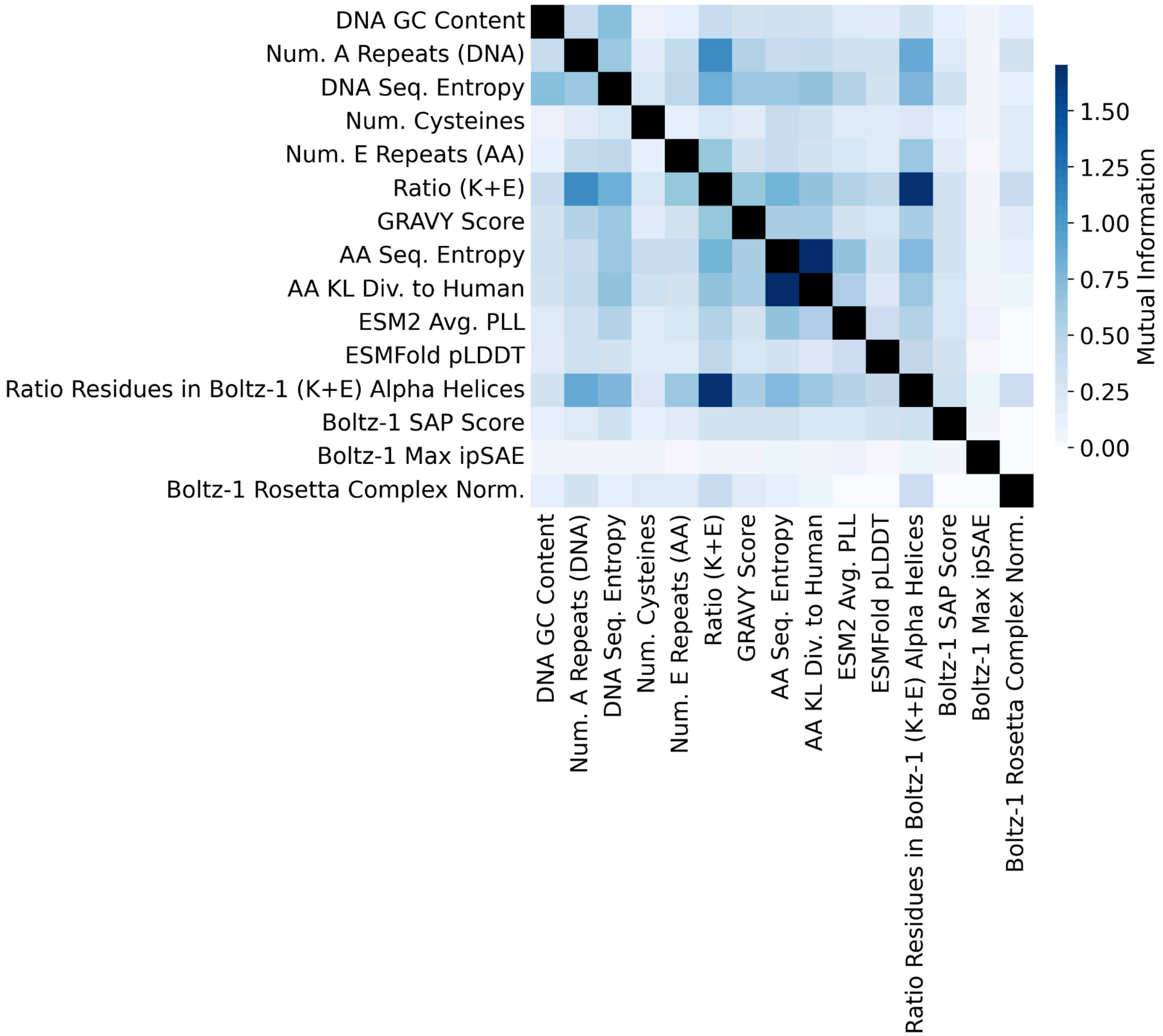

Figure SA14. Mutual information between selected metrics. Mutual information was computed across metrics from all 12,000 sequences, demonstrating correlation between the different features.

bioRxiv preprint doi: https://doi.org/10.64898/2026.03.03.709355; this version posted March 4, 2026. The copyright holder for this preprint (which was not certified by peer review) is the author/funder, who has granted bioRxiv a license to display the preprint in perpetuity. It is made available under aCC-BY 4.0 International license.

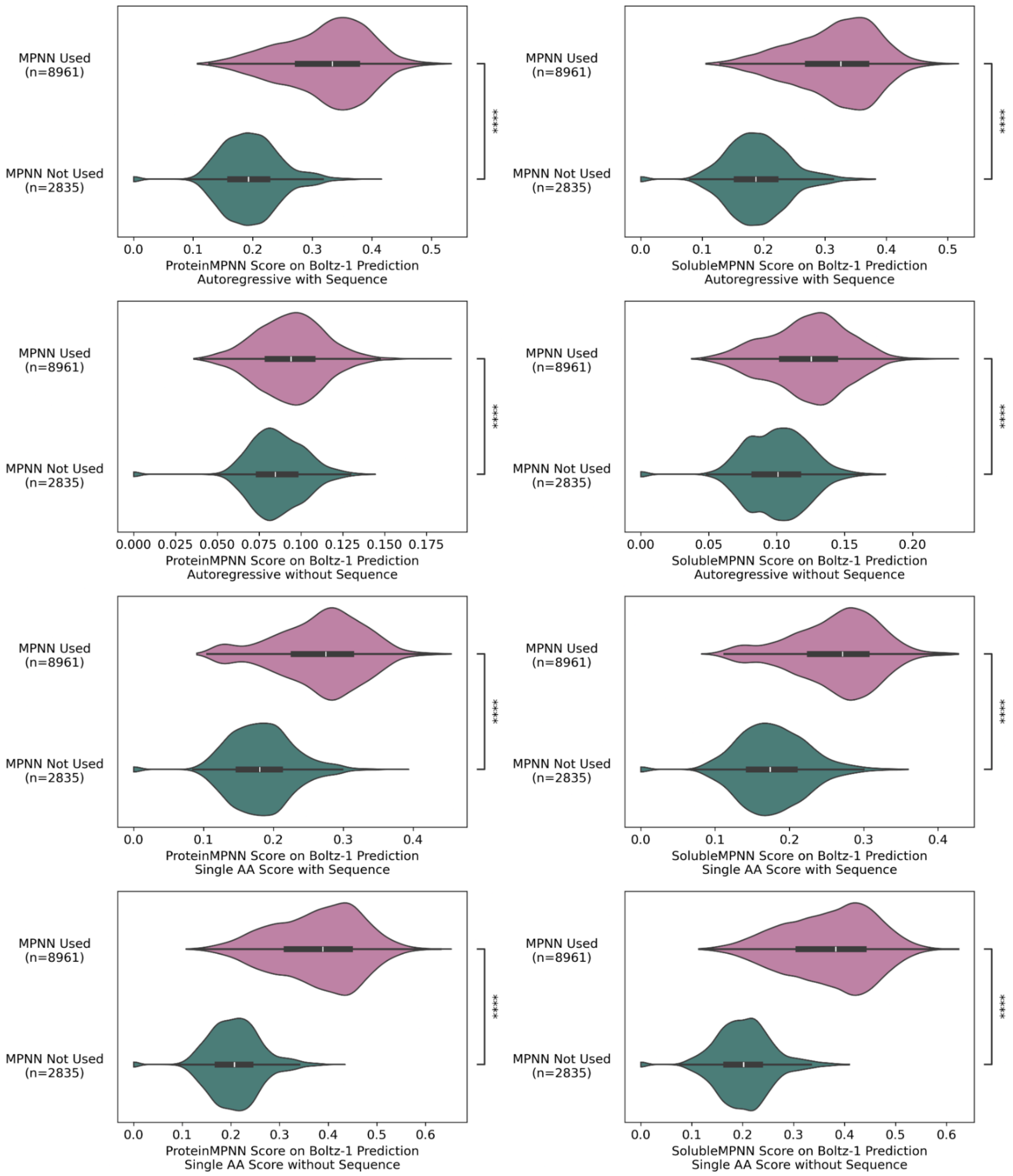

Figure SA15. ProteinMPNN and SolubleMPNN scoring is generally biased toward sequences designed by said models. ProteinMPNN and SolubleMPNN scores were obtained from the predicted Boltz-1 binder:CD20 structures. The ProteinMPNN scoring setting "autoregressive without sequence" has the least sensitivity to the use of MPNN, suggesting that this should be used over the others when the scored proteins were designed with and without an MPNN model. Significance from two-sided BH-corrected Mann-Whitney-Wilcoxon tests; ns, not significant; *, $p \leq 0.05$; **, $p \leq 0.01$; ***, $p \leq 0.001$; ****, $p \leq 0.0001$.

bioRxiv preprint doi: https://doi.org/10.64898/2026.03.03.709355; this version posted March 4, 2026. The copyright holder for this preprint (which was not certified by peer review) is the author/funder, who has granted bioRxiv a license to display the preprint in perpetuity. It is made available under aCC-BY 4.0 International license.

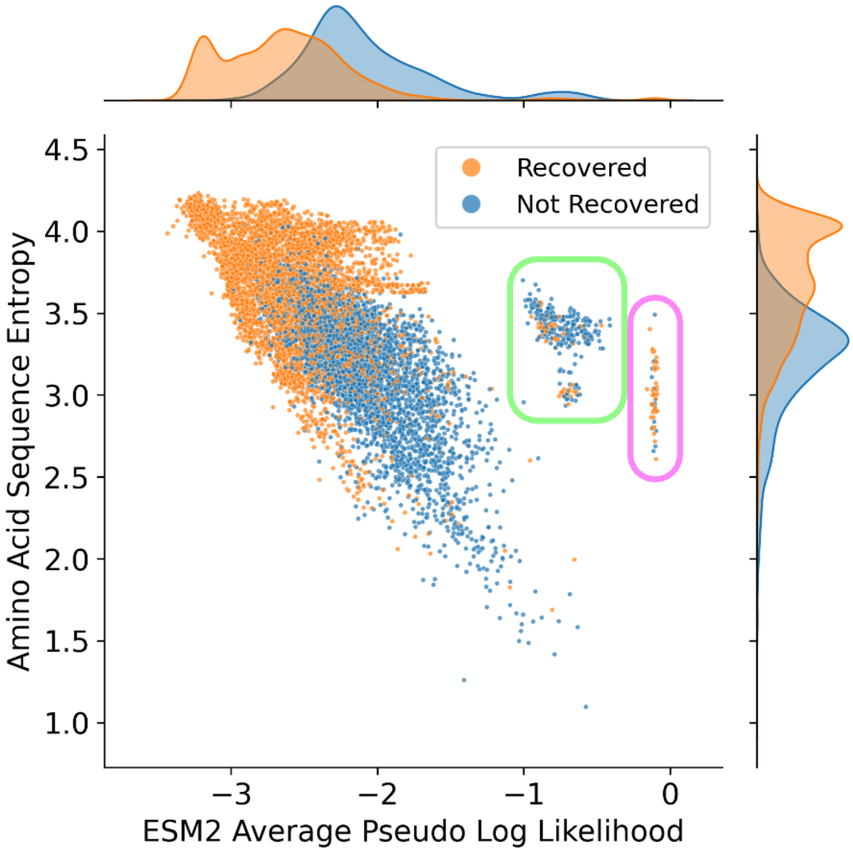

Figure SA16. ESM-2 Pseudo Log Likelihood (PLL) artifacts and entropy correlation. ESM-2 PLL (0.85 ROC-AUC) generally correlates with the Shannon entropy of the amino acid sequence, increasing as sequence entropy decreases. However, two clusters defy this trend. The green region contains only sequences from a single team, in which all 268 sequences had duplicated segments of length 29 or more, with 58-70 residues being part of a repeated substring pair. The far right vertically linear cluster, within the pink region, contains only sequences from a single other team, in which all 60 sequences had 3-4 linkers, with 60-80 of the design's residues being part of a repeated substring pair. Together, these explain the deviations from the ESM-2 PLL trend. This metric is computed by masking out one residue at a time, a task that becomes trivially easy when the answer is repeated elsewhere in the sequence. In agreement with existing literature[61], caution should be exercised when using PLL from a protein language model to down-select sequences, as they may result from syntactic sequence artifacts rather than evolutionary likelihood.

bioRxiv preprint doi: https://doi.org/10.64898/2026.03.03.709355; this version posted March 4, 2026. The copyright holder for this preprint (which was not certified by peer review) is the author/funder, who has granted bioRxiv a license to display the preprint in perpetuity. It is made available under aCC-BY 4.0 International license.

B. Computational Metrics

From the 12K binder sequences, a variety of *in silico* metrics were computed. Many metrics were computed using predicted structures from Boltz-1[67], Chai-1[31], and ESMfold[21]. To avoid modeling long disordered regions, a truncated CD20 sequence from PDB:6VJA was used, spanning residues 46 through 217.

Table SB1. Sequence-derived metrics

**Metric Name**	**Description**
DNA sequence entropy	Shannon entropy of the DNA sequences
DNA GC content	Ratio of guanine and cytosine in binder DNA sequence
Amino acid sequence entropy	Shannon entropy of the AA sequences
Number of cysteines	Number of cysteines in the binder sequence
Ratio of KE residues	Ratio of lysine and glutamate to total
Amino Acid composition KL divergence to human proteome	KL divergence between the binder's amino acid composition and that of the human proteome
Predicted charge	Computed with Bio.SeqUtils.IsoelectricPoint at pH 7
Max percent identity within team (Hamming distance)	Highest percent identity, calculated using Hamming distance, between a sequence and all other sequences from the same team. Measure of sequence uniqueness
Max percent identity cross team (Hamming distance)	Highest percent identity, calculated using Hamming distance, between a sequence and all other sequences from other teams. Measure of sequence uniqueness
Max percent identity within team (MMseqs2 alignment)	Highest percent identity, calculated using MMseqs2, between a sequence and all other sequences from the same team. Measure of sequence uniqueness
Max percent identity cross team (MMseqs2 alignment)	Highest percent identity, calculated using MMseqs2, between a sequence and all other sequences from the same team. Measure of sequence uniqueness
MMseqs2 MSA depth	Number of sequence entries found through MMseqs2
RNN solubility	Solubility prediction (0.0 to 1.0), using peptide.bio's RNN model
ESM-2 Full LL Alone	Log-likelihood for binder alone
ESM-2 Full LL CAR	Log-likelihood for CAR + binder

bioRxiv preprint doi: https://doi.org/10.64898/2026.03.03.709355; this version posted March 4, 2026. The copyright holder for this preprint (which was not certified by peer review) is the author/funder, who has granted bioRxiv a license to display the preprint in perpetuity. It is made available under aCC-BY 4.0 International license.

ESM-2 Binder LL CAR	Binder-specific log-likelihood with CAR context
ESM-2 Full LL CAR Target	Log-likelihood for CAR + binder + target
ESM-2 Binder LL CAR Target	Binder log-likelihood with CAR + target context
ESM-2 Full LL Target	Log-likelihood for binder + target
ESM-2 Binder LL Target	Binder log-likelihood with target context

Multiple sequence alignments (MSAs) were computed across all binders using MMseqs2 to UniRef30 and ColabFoldEnv databases with default Colabfold MMSeqs server parameters. 10,329 of the 12,000 binders had zero MSA depth. Boltz-1 was used to generate structures, using the Boltz MSA server whenever MMseqs2 failed to return a valid output. Rosetta FastRelax was applied to each Boltz pose prior to Rosetta InterfaceAnalyzer.

Table SB2. Boltz-1 structure-derived metrics

Metric Name	Description
DSSP secondary structure metrics	Dictionary of Secondary Structure in Proteins (DSSP) was used to assign membership to alpha helices, beta sheets, and other secondary structure elements
Ratio of residues in K+E alpha helices	The ratio of residues that are lysine or glutamate and are found in an alpha helix. This was found to be highly correlated with NGS recovery after growth, and is also indicative of ProteinMPNN.
Hotspots engaged	Ratio of residues in large and small loops of CD20 that are engaged by the binder.
Hotspot PAE, iPLDDT, PDE	Values from Boltz matrix subsetted at the hotspot positions.
SAP Score	Spatial Aggregation Propensity, computed from Rosetta.
Rosetta InterfaceAnalyzer	All metrics from Rosetta InterfaceAnalyzer were computed: https://docs.rosettacommons.org/docs/latest/application_documentation/analysis/interface-analyzer
Angle of binder N/C terminii relative to central axis of CD20	This was computed to check whether geometric constraints determine binder success.
Binder location relative to membrane	Binder's COM displacement from origin. Positive means outer membrane.
ipSAE[55]	https://github.com/DunbrackLab/IPSAE

bioRxiv preprint doi: https://doi.org/10.64898/2026.03.03.709355; this version posted March 4, 2026. The copyright holder for this preprint (which was not certified by peer review) is the author/funder, who has granted bioRxiv a license to display the preprint in perpetuity. It is made available under aCC-BY 4.0 International license.

LIS score[56]	https://github.com/flyark/AFM-LIS
pDockQ[59]	https://www.nature.com/articles/s41467-022-28865-w
pDockQ2[60]	https://academic.oup.com/bioinformatics/article/39/7/btad424/7219714
Number of predicted disulfide bonds (within chain)	Predicted disulfides within the binder chain only. Disulfides were found by collecting all CYS residues with an SG atom, checking every pair's SG–SG distance, and recording pairs within 3.0 Å
Number of predicted disulfide bonds (between chains)	Predicted disulfides between the binder and CD20. Disulfides were found by collecting all CYS residues with an SG atom, checking every pair's SG–SG distance, and recording pairs within 3.0 Å
Number of predicted pi-pi interactions (within chain)	Predicted pi-pi interactions within the binder chain only. Pi-pi was found by collecting aromatic residues (PHE/TYR/TRP/HIS), computing each ring's centroid and plane normal from selected ring atoms, and recording residue pairs whose centroids are within 7.0 Å and whose ring-plane normals are within 30°
Number of predicted pi-pi interactions (between chains)	Predicted pi-pi interactions between the binder and CD20. Pi-pi was found by collecting aromatic residues (PHE/TYR/TRP/HIS), computing each ring's centroid and plane normal from selected ring atoms, and recording residue pairs whose centroids are within 7.0 Å and whose ring-plane normals are within 30°
ProteinMPNN autoregressive with sequence	Likelihood derived from ProteinMPNN using:<br>–autoregressive 1<br>–use_sequence 1
ProteinMPNN autoregressive without sequence	Likelihood derived from ProteinMPNN using:<br>–autoregressive 1<br>–use_sequence 0
ProteinMPNN single AA with sequence	Likelihood derived from ProteinMPNN using:<br>–single_aa_score 1<br>–use_sequence 1
ProteinMPNN single AA without sequence	Likelihood derived from ProteinMPNN using:<br>–single_aa_score 1<br>–use_sequence 0
SolubleMPNN autoregressive with sequence	Likelihood derived from SolubleMPNN using:<br>–autoregressive 1<br>–use_sequence 1
SolubleMPNN autoregressive	Likelihood derived from SolubleMPNN using:

bioRxiv preprint doi: https://doi.org/10.64898/2026.03.03.709355; this version posted March 4, 2026. The copyright holder for this preprint (which was not certified by peer review) is the author/funder, who has granted bioRxiv a license to display the preprint in perpetuity. It is made available under aCC-BY 4.0 International license.

without sequence	–autoregressive 1<br>–use_sequence 0
SolubleMPNN single AA with sequence	Likelihood derived from SolubleMPNN using:<br>–single_aa_score 1<br>–use_sequence 1
SolubleMPNN single AA without sequence	Likelihood derived from SolubleMPNN using:<br>–single_aa_score 1<br>–use_sequence 0

Given that ESMFold does not natively multimer input, we introduced a shift in the positional encoding and/or a glycine linker. We tested different chain permutations depending on the position of the binder (start, middle, end); as well as with or without the glycine linker (mode_glycine_linker). The following scores were computed on these structures.

Table SB3. ESMFold structure-derived metrics.

**Metric Name**	**Description**
pTM, iPAE, iPTM, pLDDT	Common folding metrics
Binder location relative to membrane	Binder's COM displacement from origin. Positive means outer membrane.
ipSAE	https://github.com/DunbrackLab/IPSAE
LIS score	https://github.com/flyark/AFM-LIS
pDockQ	https://www.nature.com/articles/s41467-022-28865-w
pDockQ2	https://academic.oup.com/bioinformatics/article/39/7/btad424/7219714

Table SB4. Chai-1 structure-derived metrics

**Metric Name**	**Description**
pTM, iPAE, iPTM, pLDDT	Common folding metrics
Binder location relative to membrane	Binder's COM displacement from origin. Positive means outer membrane.
ipSAE	https://github.com/DunbrackLab/IPSAE
LIS score	https://github.com/flyark/AFM-LIS

bioRxiv preprint doi: https://doi.org/10.64898/2026.03.03.709355; this version posted March 4, 2026. The copyright holder for this preprint (which was not certified by peer review) is the author/funder, who has granted bioRxiv a license to display the preprint in perpetuity. It is made available under aCC-BY 4.0 International license.

pDockQ	https://www.nature.com/articles/s41467-022-28865-w
pDockQ2	https://academic.oup.com/bioinformatics/article/39/7/btad424/7219714

Table SB5. Twist measured values

Measurement Name	Description
Twist DNA read percentile	The percentile of reads in Twist's report. Normalized by norm=(raw read number for that seq/324737) * 1000. Zero indicates not detected.

Table SB6. LEAH 12K proliferation values

Measurement Name	Description
K: CD20- NGS reads (x3)	NGS counts after proliferation without presence of CD20+ cells
R: CD20+ NGS reads (x3)	NGS counts after proliferation in presence of CD20+ cells
logFC	log2(R/K)
logCPM	log counts per million
FDR	False discovery rate
Recovered	<25 reads in any of the six replicates
Fourclass	Up: ≥2-fold logFC and significant based on FDR<br>Down: ≤2-fold logFC and significant based on FDR<br>Not Sig: Not significant based on FDR<br>Not Recovered: <25 reads in any of the six replicates
Final score	-log10(FDR)*logFC

Table SB7. LEAH individual functional assays

Measurement Name	Description
Cytotoxicity (K: CD20-)	Measure of how much the CAR-T cells kill CD20- cells
Cytotoxicity (R: CD20+)	Measure of how much the CAR-T cells kill CD20+ cells
Cytotoxicity (R-K)	Measure of how much the CAR-T cells preferentially kill CD20+ cells
% cytokine+	Measure of how what percentage of T cells produce the expected cytokines

bioRxiv preprint doi: https://doi.org/10.64898/2026.03.03.709355; this version posted March 4, 2026. The copyright holder for this preprint (which was not certified by peer review) is the author/funder, who has granted bioRxiv a license to display the preprint in perpetuity. It is made available under aCC-BY 4.0 International license.

Fold change CTV MFI AUC	Measure of proliferation. Fold change in CellTrace Violet Mean Fluorescence Intensity Area Under Curve. Indicative of proliferation, as intensity decreases as cells proliferate.
Fold change total cell count per well	Measure of expansion.
Sum of min/max normalized scores	The following scores were min/max normalized to the negative/positive controls: Cytotoxicity (R-K), % cytokine+, Fold change CTV MFI AUC. This was done to determine the best-performing binders across three orthogonal T cell functions: specific cell killing, cytokine release, and proliferation.

bioRxiv preprint doi: https://doi.org/10.64898/2026.03.03.709355; this version posted March 4, 2026. The copyright holder for this preprint (which was not certified by peer review) is the author/funder, who has granted bioRxiv a license to display the preprint in perpetuity. It is made available under aCC-BY 4.0 International license.

C. Competition Information, Discussion, and Lessons

Abbreviated timeline

May 2024: Initial planning begins
June 2024: Sponsor acquisition
July 16th, 2024: Public announcement
August 24th, 2024: Kickoff event
September 31st, 2024: Submission deadline (original)
October 7th, 2024: Submission deadline (post-extension)
October 2024: Sequences ordered
Jan 30th, 2025: DNA arrived from Twist to UT Austin. Shipped to LEAH Labs
May 2025: 12K proliferation results announcement
August 2025: Functional assays for top 10 completed
September 2nd, 2025: Winning teams announced
November 2025: Adaptyv begins binding affinity study
December 2025: Adaptyv completes binding affinity study

Entrant statistics

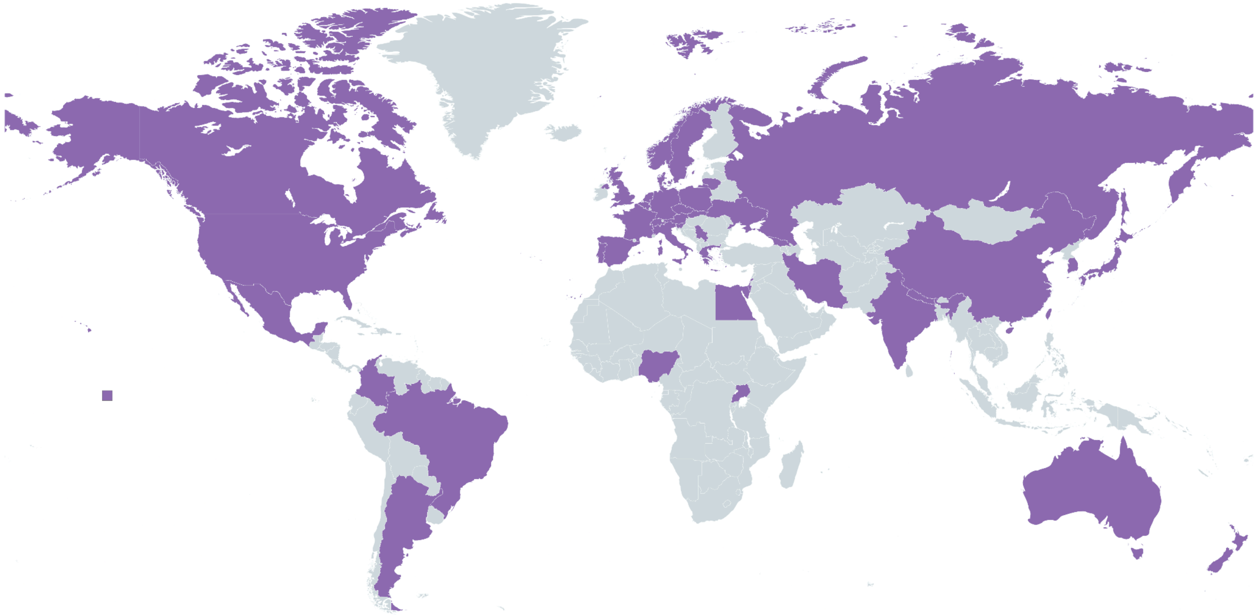

Figure SC1. Countries of citizenship reported by competition entrants. Entrants span 42 countries across 6/7 continents. Data collected from the 169 competition RSVPs and the team formation responses. Map created with mapchart.net.

bioRxiv preprint doi: https://doi.org/10.64898/2026.03.03.709355; this version posted March 4, 2026. The copyright holder for this preprint (which was not certified by peer review) is the author/funder, who has granted bioRxiv a license to display the preprint in perpetuity. It is made available under aCC-BY 4.0 International license.

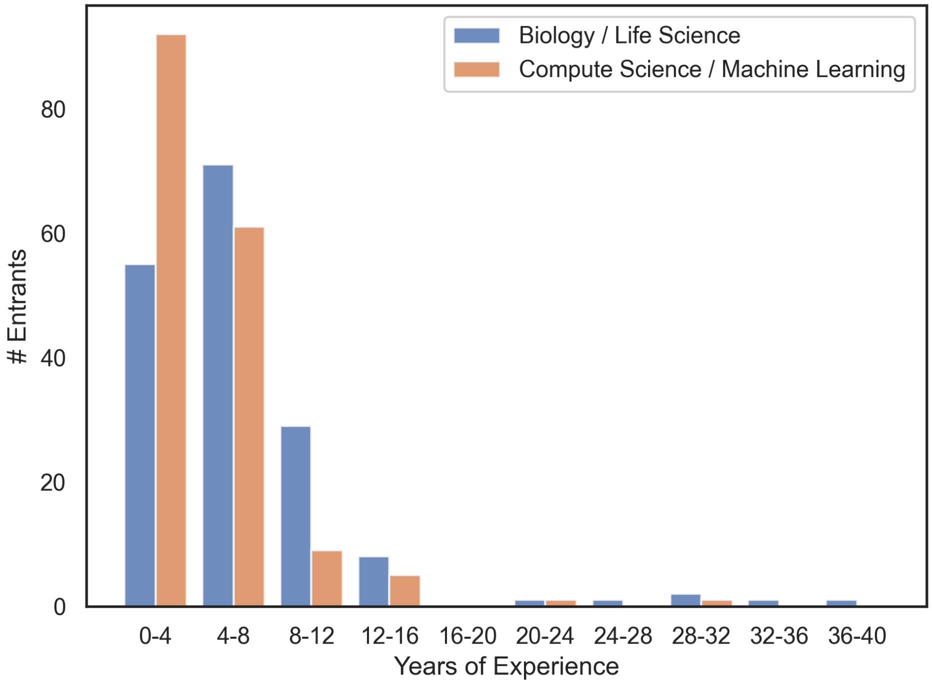

Figure SC2. Competition entrant years of experience. Data collected from the 169 competition RSVPs and the team formation responses.

Intellectual property considerations and avoiding conflicts of interest

Given the fact that competitors from academic and commercial entities were designing potential therapeutics validated by commercial entities, we required a very clear stance on how intellectual property would be handled. After considering the various options, it was deemed that the best option was for everyone to release all claims on intellectual property, and that LEAH Labs would not commercialize any of the submitted sequences. This was further complicated by some models having licenses that forbid commercial entities from testing the created proteins. To avoid any conflicts, competitors were required to acknowledge and agree to a participant agreement that explicitly addressed both intellectual property rights and the use of commercial software. This agreement was as follows:

<u>Participant Agreement:</u>

1. Intellectual Property: No intellectual property (IP) rights will be claimed on the designed sequences. All designed sequences must be released to the public domain. The organizers and sponsors will not be claiming intellectual property rights to the designed sequences nor will they be commercializing them.
2. Open Source and Public Availability Requirement:
    a. All datasets, models, methods, and code developed or used during the competition should be made open source whenever possible.

bioRxiv preprint doi: https://doi.org/10.64898/2026.03.03.709355; this version posted March 4, 2026. The copyright holder for this preprint (which was not certified by peer review) is the author/funder, who has granted bioRxiv a license to display the preprint in perpetuity. It is made available under aCC-BY 4.0 International license.

b. We discourage teams from using closed-source and license-restrictive models such as AlphaFold3 and ESM-3. However, we cannot police this given the number of submissions we are dealing with, so by submitting your designs you agree that the burden of responsibility for all software used rests upon you, the submitting party, and not the organizers or sponsors.

Further, the participant represents that they have the right to use any software, models, tools or data that they have used or will use to produce any entry in the competition. Participant also represents that Participant (i) has not filed a patent application or submitted an invention disclosure to Participant's employer or institution for the purpose of a patent application being filed, and (ii) is not aware of any invention disclosure or patent application, that attempts to protect or cover the sequence, sequences, molecule or molecules, or any method of using them, that Participant has submitted or will submit as an entry in the competition. Participant further represents that Participant will not in the future file such an application or submit such an invention disclosure to an employer or institution.

Kickoff event

A 2-hour remote kickoff event was held on Saturday August 24th, 2024. The purpose of this event was to formally reveal the design target, explain the logistics in more detail, and more. The event proceeded with the following schedule:

- Motivation for the competition
- Sponsor talks: Lonza Group, ScaleReady, Twist Bioscience, LEAH Labs
- Problem statement, target reveal, CAR-T background information
- Submission requirements, IP considerations
- Experimental validation overview and winners selection process
- Example binder design workflow
- Compute options
- Closing remarks
- Office hours

Slides: https://github.com/kosonocky/bits-to-binders/blob/main/misc/kickoff_slides.pdf
Recording: https://www.youtube.com/watch?v=fSoEX0c-Gko

Resources provided to competitors

A compilation of resources was provided to the competitors after the kickoff event. This included papers, code, and web-servers for: protein structure prediction, inverse folding, generative models, language models, and other relevant tools. Additional papers and lectures were provided on computational protein design, along with several reviews on CAR-T therapies and CD20. Links to guides on using TACC, Synthia, and additional

bioRxiv preprint doi: https://doi.org/10.64898/2026.03.03.709355; this version posted March 4, 2026. The copyright holder for this preprint (which was not certified by peer review) is the author/funder, who has granted bioRxiv a license to display the preprint in perpetuity. It is made available under aCC-BY 4.0 International license.

tools suggested by Adaptyv Bio were additionally provided. These resources can be found at: https://github.com/kosonocky/bits-to-binders-resources.

Compute support

Competitors were given access to compute at the Texas Advanced Computing Center (TACC) Vista GH200 supercluster. Many competitors did not use this resource as many models and open source software were not yet compatible and compiled for Vista's ARM operating system. This highlights a need for computational resources to be aligned with the user expertise for usability.

Miscellaneous logistics

Advertisement for the competition went out on the organizer's social media platforms (LinkedIn and X), university communications, and communications from the competition's sponsors. An RSVP form was sent out with the announcement. This form was used to collect competitor demographics and was used to add people to a Slack channel for further announcements. In this channel, people without teams were encouraged to reach out to one another and form teams on their own. Throughout the event, announcements were primarily made through this Slack channel, though occasional communications went out over email. The main disadvantage of using the free version of Slack for an event like this is that messages disappear after 90 days, meaning that past communications become inaccessible.

The kickoff event was hosted virtually, with organizers present in-person in Austin, Texas to ensure a smooth rollout. The event was on Saturday at the end of the summer prior to the beginning of the fall semester to improve attendance from both industry and academic participants.

Determination of winning teams

From the 12K proliferation screen, ten of the top-performing non-redundant designs were chosen to proceed to a series of individual CAR-T functional screens. From these top ten, data on proliferation, expansion, cytotoxicity, and cytokine production were obtained. A final score was created from this data to best reflect overall performance without being biased to a given behavior. Given their probable correlation, proliferation was withheld from the final score and just expansion was retained. Values for cytotoxicity, cytokine, and expansion were min/max normalized to the negative/positive controls, and summed for the "final score". Designs were then ranked by this final score, and the top three designs were deemed 1st, 2nd, and 3rd place. A fourth winner was also announced to be the team with the highest percentage of successful sequences in the 12K proliferation screen.

The top three teams by 12K screen hit rate were as follows:

bioRxiv preprint doi: https://doi.org/10.64898/2026.03.03.709355; this version posted March 4, 2026. The copyright holder for this preprint (which was not certified by peer review) is the author/funder, who has granted bioRxiv a license to display the preprint in perpetuity. It is made available under aCC-BY 4.0 International license.

1. *Nucleate UK London (38.4%)
2. Binding Illini (14.8%)
3. Furman Lab (11.1%)

The top three finalist teams by the min/maxed “final score” were as follows:
1. *Perez Lab Gators (1.99/3.00)
2. *Amigo Acids (1.95/3.00)
3. *Schoeder Lab (1.34/3.00)

Each winning team, designated with an asterisk, was gifted a 3D-printed trophy of their successful binder. For Nucleate UK London, a random successful binder was used.

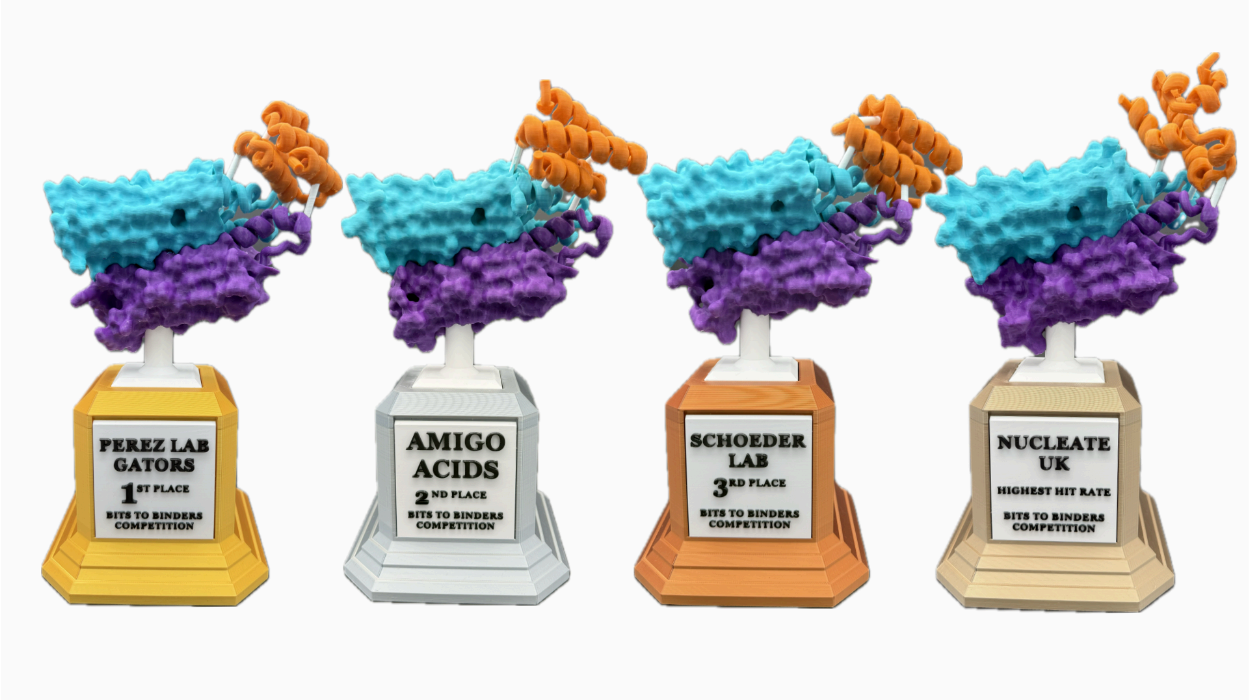

Figure SC3. 3D-printed trophies for the winning teams. Trophies were given to the three teams with the best individual sequences and to the team with the highest hit rate in the 12K proliferation screen.

bioRxiv preprint doi: https://doi.org/10.64898/2026.03.03.709355; this version posted March 4, 2026. The copyright holder for this preprint (which was not certified by peer review) is the author/funder, who has granted bioRxiv a license to display the preprint in perpetuity. It is made available under aCC-BY 4.0 International license.

Feedback to organizers

Feedback was obtained from 14 teams that entered the competition. All of these teams except one submitted sequences, with the remaining one not submitting due to scheduling conflicts.

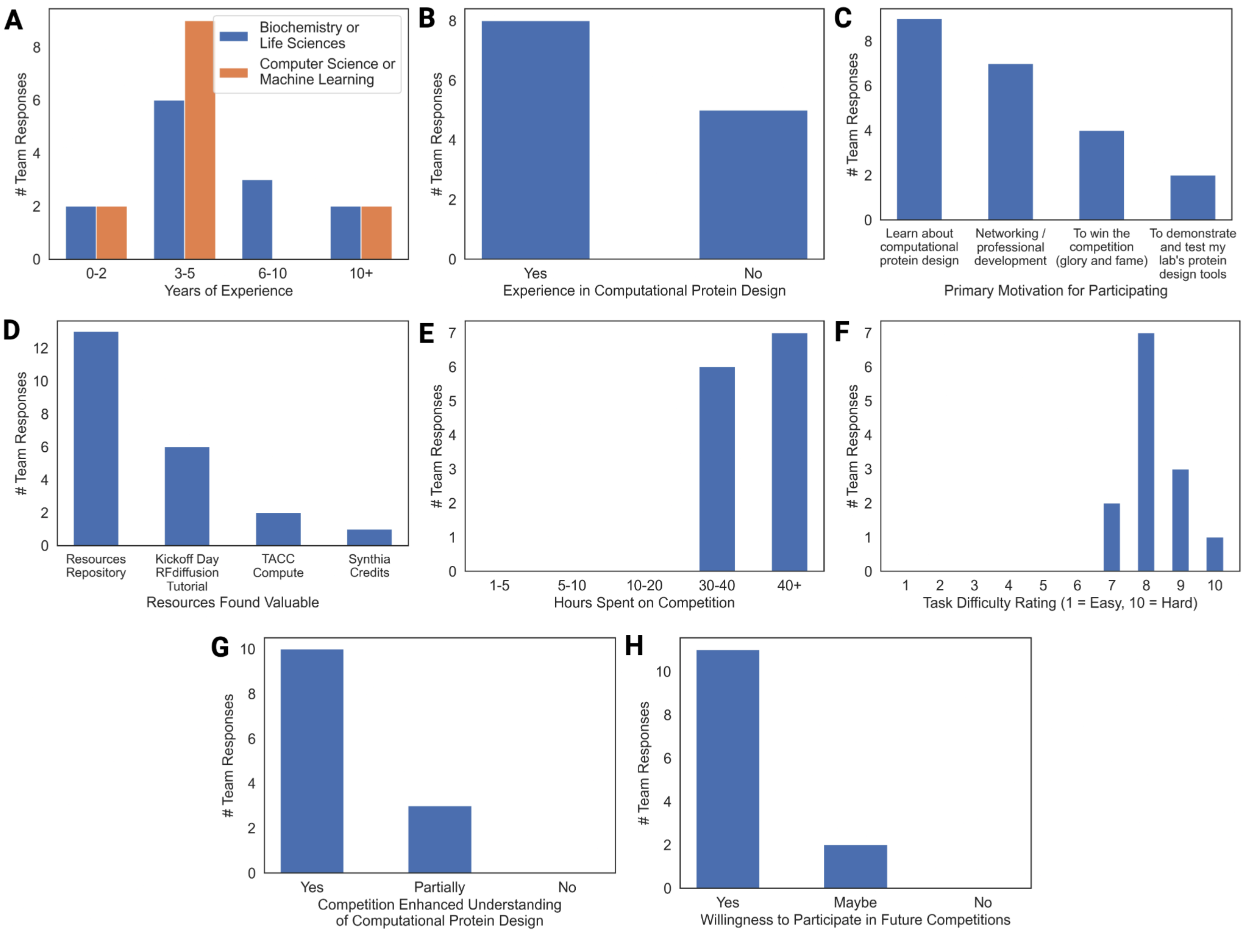

Figure SC4. Survey response from 13 teams that submitted designs. (A) Years of experience in biochemistry, life sciences, computer science, and machine learning. **(B)** Previous experience in computational protein design. **(C)** Primary reason for entering the competition. Survey response from 13 teams that submitted designs. One other reason for entering was "I met all of the people on my team at a summer internship at the Baker lab. We wanted to stay in touch!!". **(D)** Provided resources that were deemed valuable. **(E)** Hours spent on the competition. **(F)** Protein design task perceived difficulty. **(G)** Enhancement of participant understanding in computational protein design. **(H)** Participant interest in future competitions.

<u>Additional Question 1. If [you didn't find any of the provided resources useful], why? Which additional resources, if any, would have been useful?</u>

"It was very difficult to coordinate and manage compute resources and the data between them"

bioRxiv preprint doi: https://doi.org/10.64898/2026.03.03.709355; this version posted March 4, 2026. The copyright holder for this preprint (which was not certified by peer review) is the author/funder, who has granted bioRxiv a license to display the preprint in perpetuity. It is made available under aCC-BY 4.0 International license.

"Easier setup process to access TACC computer resources, I was using custom scripts so didn't need synthia credits, and I have used rfdiffusion before"
"AWS or GCP credits"

Additional Question 2. Did you find participating in the competition worthwhile for other reasons? Why or why not?
"I feel like it was extremely fun and challenging. I learned a lot about state of the art techniques for binding to hydrophilic surface proteins, something that I didn't know a lot about before the competition."
"It was great to have a fully remote networking opportunity, and to work on my teamwork skills."
"Interesting to work on a target that one would usually avoid for binder design (small extracellular domain, no known native interaction)"
"Yes, collaboration with different individuals"
"Absolutely. I learned a lot of domain-specific knowledge that I wasn't able to get."
"I was testing my scientific collaboration platform to address the difficulties in scientific collaboration"

Additional Question 3. What aspects of the competition worked well?
"I think the overall competition and the instructions were very clear"
"Resources and kickoff"
"It's awesome that you test so many of the designs directly in CARs"
"Discussion session and help session"
"timeline"
"Github repos, tutorials"

Additional Question 4. What changes or improvements would you like to see in future competitions?
"The process of finding a team in Slack was a little awkward. Maybe some clear guidance at the beginning about how to find teammates would have made this part a little smoother."
"Better documentation for connecting to and using TACC resources with common models."
"Easier to access resources (more user friendly), and more closing communication / followup"
"More office hours in regards to infrastructure"
"Sharing workflows between teams"

Additional Question 5. What topic would you like to see a competition focused on in the future?

bioRxiv preprint doi: https://doi.org/10.64898/2026.03.03.709355; this version posted March 4, 2026. The copyright holder for this preprint (which was not certified by peer review) is the author/funder, who has granted bioRxiv a license to display the preprint in perpetuity. It is made available under aCC-BY 4.0 International license.

"Enzymes!!!"
"Designing proteins/peptides for selective purification of lanthanide metals"
"Enzyme design"
"Enzyme activity, peptide binders"
"Spatial Transcriptomics that can be verified in the lab"
"Drug interaction, kinetic improvement of enzymes"

Additional Question 6. Do you have any additional feedback, comments, or suggestions?

"Do an enzyme competition. I think at this time next year the tech will be available and now that AF3 is out this would be such a cool contest!"
"Many thanks to the organizers for this great opportunity!"
"This was an awesome competition. I learned a lot!"
"A great competition idea, thank you for making it possible."

bioRxiv preprint doi: https://doi.org/10.64898/2026.03.03.709355; this version posted March 4, 2026. The copyright holder for this preprint (which was not certified by peer review) is the author/funder, who has granted bioRxiv a license to display the preprint in perpetuity. It is made available under aCC-BY 4.0 International license.

D. Sponsor Contributions

The *Bits to Binders* competition benefitted considerably from a number of academic and industrial sponsors. This competition would not have been possible without their generous support.

LEAH Laboratories:

Website:

https://www.leahlabs.com/

Contribution:

LEAH Labs helped plan and run the competition. Utilizing their patented precision integration technology developed in primary human T cells, LEAH Labs conducted pooled library integration and high-throughput screening as well as performed all experimental validation on T cells, and analyzed the experimental results.

Adaptyv Bio:

Website:

https://www.adaptyvbio.com/

Contribution:

Adaptyv Bio provided triplicate binding affinity measurements for 100 binders to CD20 free-of-charge.

Twist Bioscience:

Website:

https://www.twistbioscience.com/

Contribution:

Twist provided a 50% discount on the plasmid pools.

The University of Texas at Austin Center for Systems and Synthetic Biology:

Website:

https://www.facebook.com/CSSBUT2015/

Contribution:

The UT Austin Center for Systems and Synthetic Biology provided funding to purchase the DNA and the trophies.

bioRxiv preprint doi: https://doi.org/10.64898/2026.03.03.709355; this version posted March 4, 2026. The copyright holder for this preprint (which was not certified by peer review) is the author/funder, who has granted bioRxiv a license to display the preprint in perpetuity. It is made available under aCC-BY 4.0 International license.

Texas Advanced Computing Center:
Website:
https://tacc.utexas.edu/

Contribution:
TACC provided free compute to competitors on their Vista GH200 superchip cluster.

Modal:
Website:
https://modal.com/

Contribution:
Modal provided $5K in compute credits that allowed J.L. to compute *in silico* metrics for the competition measurement data.

Lonza:
Website:
https://www.lonza.com/

Contribution:
Lonza provided reagents for CAR-T cell experiments.

ScaleReady:
Website:
https://www.scaleready.com/

Contribution:
ScaleReady provided reagents for CAR-T cell experiments.

VWR:
Website:
https://www.vwr.com/

Contribution:
VWR provided reagents for CAR-T cell experiments.

bioRxiv preprint doi: https://doi.org/10.64898/2026.03.03.709355; this version posted March 4, 2026. The copyright holder for this preprint (which was not certified by peer review) is the author/funder, who has granted bioRxiv a license to display the preprint in perpetuity. It is made available under aCC-BY 4.0 International license.

KUNGFU.AI:
Website:
https://www.kungfu.ai/

Contribution:
KUNGFU.AI provided AI consulting to the competitors during the kickoff event.

Maker Clinic:
Website:
linkedin.com/in/adamsalmi

Contribution:
Maker Clinic provided custom 3D-printed trophies for the winning binders at a discounted rate.

Synthia:
Website:
https://www.synthialabs.com/

Contribution:
Synthia provided credits to competitors to use their protein design LLM.

The University of Texas at Austin; Dell Medical School:
Website:
https://dellmed.utexas.edu/

Contribution:
The UT Austin Dell Medical School helped advertise the competition and facilitate some of the connections to sponsors.

Nucleate AI in Biotech:
Website:
https://nucleate.org/

Contribution:
Nucleate AIxBio helped advertise the competition.

bioRxiv preprint doi: https://doi.org/10.64898/2026.03.03.709355; this version posted March 4, 2026. The copyright holder for this preprint (which was not certified by peer review) is the author/funder, who has granted bioRxiv a license to display the preprint in perpetuity. It is made available under aCC-BY 4.0 International license.

The BioML Society:
Website:
https://www.biomlsociety.org/

Contribution:
The BioML Society formulated, organized, and ran the competition.

bioRxiv preprint doi: https://doi.org/10.64898/2026.03.03.709355; this version posted March 4, 2026. The copyright holder for this preprint (which was not certified by peer review) is the author/funder, who has granted bioRxiv a license to display the preprint in perpetuity. It is made available under aCC-BY 4.0 International license.

E. *Bits to Binders* Teams, Competitors, and Design Methods

At the time of design submission, teams were asked to provide an abstract on the methods used as well as steps to reproduce the designs. Email addresses are listed for the designated primary team contacts. Questions regarding a team's methods should be directed toward these individuals.

1. AIBI

Team Members:

Yang Shen (yshen@tamu.edu), Shaowen Zhu, Yuxuan Liu, Rujie Yin, Dishant Parag Zaveri, Sai Jaideep Reddy Mure, Pragati Naikare

Abstract:

We mainly used a joint sequence-structure co-design model, which is a diffusion model conditioned on target CD20 structure & epitope, without or with the fixed context's sequence and structure of the designed protein. We made multimer inference using three diffusion model variants and monomer inference with linkers across chains using the last variant. And we combined the four types of model inferences with five types of epitopes (three for known CD20 antibodies, the union of the three, and the entire extracellular domain) and generated 180 designs for each of the 20 combinations. The resulting 3600 designs are geometrically ranked based on contact consistency with intended epitopes. We also used a pretrained sequence-alone ESM2[21] to impute the 80 amino acids from the context sequence and picked the top 50 from the 200 designs based on PIPR-predicted[68] affinity to CD20.

Reproducibility:

Method A: Main method, no citation yet, designs 1-270 and 301-480.

A1. Joint sequence-structure generation using target (and scaffold) conditioned diffusion models

A2. Model selection using curated cocrystal structures between CD20/CD19 and antibodies

A3. Filtering and ranking designed structures based on consistency with desired contacts

Method B: ESM2 (650M parameters), designs 271-300 and 481-500.

B1. Sequence imputation from the rest of CAR sequences by iterative sampling of a pretrained ESM2, which is not specific to CD20.

B2. Filtering and ranking designed sequences based on sequence-predicted binding affinity to CD20

bioRxiv preprint doi: https://doi.org/10.64898/2026.03.03.709355; this version posted March 4, 2026. The copyright holder for this preprint (which was not certified by peer review) is the author/funder, who has granted bioRxiv a license to display the preprint in perpetuity. It is made available under aCC-BY 4.0 International license.

2. Amigo Acids

Team Members:
Antonio Fonseca (ahof1704@gmail.com), Joseph Openy, Ava Chan, Freddie Martin, Ansar Ahmad Javed, Dionessa Biton, Shreyasi Das, Francisco Requena

Abstract:
We used a combination of free diffusion with RFdiffusion[16] constrained by length and using hotspots located on the extracellular portion of the CD20. With the designs that showed reasonable binding affinity, we performed partial diffusion followed by FastRelax to obtain new proteins that could bind more effectively. The ranking of the design was done with AF2[29].

Reproducibility:
- Free diffusion with RFdiffusion (80 AAs) using hotspots
- ProteinMPNN[23] to obtain sequences
- AFPulldown[69] for structure assessment
- Sort designs by ipae and iptm
- Perform partial diffusion with RFdiffusion
- Predict sequences using ProteinMPNN+FastRelax
- Predict structure using ColabFold[30]

3. Antigeniuses

Team Members:
Alia Clark-ElSayed (ac86387@my.utexas.edu), Satoshi Ishida, Daniel Acosta, Tynan Gardner

Abstract:
We generated a list of protein sequences that are known to bind to CD20. Due to the fact that this was over 500 sequences, we wanted to down-select to the top ~10 binders. To do this we used AlphaFold-Multimer[70] to co-fold the binder with CD20. Based on the model scores, we selected the top 10 sequences to use for further design and diversification. We diversified these structures with RFdiffusion and then generated the sequences using ProteinMPNN.

Reproducibility:
- Generated list of CD20 binders from databases
- AlphaFold Multimer to assess binding (https://www.biorxiv.org/content/10.1101/2021.10.04.463034v2)

bioRxiv preprint doi: https://doi.org/10.64898/2026.03.03.709355; this version posted March 4, 2026. The copyright holder for this preprint (which was not certified by peer review) is the author/funder, who has granted bioRxiv a license to display the preprint in perpetuity. It is made available under aCC-BY 4.0 International license.

- RFDiffusion (https://www.nature.com/articles/s41586-023-06415-8)
- ProteinMPNN (https://www.science.org/doi/10.1126/science.add2187)

4. Binder Bot Engineers

Team Members:

Genhui Zheng (genhuizheng@utexas.edu), Rajarshi Mondal, Calvin XiaoYang Hu, Aoi Otani, Aparna Sahu

Abstract:

In this binder design experiment, we employ two approaches—active learning (AL)-based methods and traditional strategies—for antibody sequence generation. First, these methods are used to generate initial template sequences, which are then filtered using binding affinity prediction tools to select promising candidates.

Next, from the filtered templates, we create a diverse mutational library by introducing amino acid mutations, insertions, and deletions. This library is further evaluated using stability and other protein property prediction tools to ensure viability and functionality.

Finally, the sequences are ranked based on their predicted binding affinity, stability, and additional relevant properties. The top 500 sequences are selected for submission.

Reproducibility:

Sequence Generation:

Utilize both active learning (AL)-based methods and traditional methods to generate initial antibody template sequences [1-2][23,71] .

Binding Affinity Filtering:

Apply binding affinity prediction tools to filter and retain only the most promising sequences[3][29].

Library Creation:

Generate a diverse sequence library by performing mutations, insertions, and deletions on the filtered templates to explore a wide range of amino acid variants.

Property Evaluation:

Use tools to evaluate stability and other key protein properties (e.g., solubility, aggregation, immunogenicity) for the entire sequence library [4][72].

Ranking and Selection:

bioRxiv preprint doi: https://doi.org/10.64898/2026.03.03.709355; this version posted March 4, 2026. The copyright holder for this preprint (which was not certified by peer review) is the author/funder, who has granted bioRxiv a license to display the preprint in perpetuity. It is made available under aCC-BY 4.0 International license.

Rank sequences based on binding affinity, stability, and additional protein properties. Select the top 500 sequences for submission.

[1]. Dauparas J, Anishchenko I, Bennett N, Bai H, Ragotte RJ, Milles LF, Wicky BIM, Courbet A, de Haas RJ, Bethel N, Leung PJY, Huddy TF, Pellock S, Tischer D, Chan F, Koepnick B, Nguyen H, Kang A, Sankaran B, Bera AK, King NP, Baker D. Robust deep learning-based protein sequence design using ProteinMPNN. Science. 2022 Oct 7;378(6615):49-56. doi: 10.1126/science.add2187. Epub 2022 Sep 15. PMID: 36108050; PMCID: PMC9997061.
[2]. Manso T, Folch G, Giudicelli V, Jabado-Michaloud J, Kushwaha A, Nguefack Ngoune V, Georga M, Papadaki A, Debbagh C, Pégorier P, Bertignac M, Hadi-Saljoqi S, Chentli I, Cherouali K, Aouinti S, El Hamwi A, Albani A, Elazami Elhassani M, Viart B, Goret A, Tran A, Sanou G, Rollin M, Duroux P, Kossida S. IMGT® databases, related tools and web resources through three main axes of research and development. Nucleic Acids Res. 2022 Jan 7;50(D1):D1262-D1272. doi: 10.1093/nar/gkab1136. PMID: 34875068; PMCID: PMC8728119.
[3]. Jumper J, Evans R, Pritzel A, Green T, Figurnov M, Ronneberger O, Tunyasuvunakool K, Bates R, Žídek A, Potapenko A, Bridgland A, Meyer C, Kohl SAA, Ballard AJ, Cowie A, Romera-Paredes B, Nikolov S, Jain R, Adler J, Back T, Petersen S, Reiman D, Clancy E, Zielinski M, Steinegger M, Pacholska M, Berghammer T, Bodenstein S, Silver D, Vinyals O, Senior AW, Kavukcuoglu K, Kohli P, Hassabis D. Highly accurate protein structure prediction with AlphaFold. Nature. 2021 Aug;596(7873):583-589. doi: 10.1038/s41586-021-03819-2. Epub 2021 Jul 15. PMID: 34265844; PMCID: PMC8371605.
[4]. Wilkins MR, Gasteiger E, Bairoch A, Sanchez JC, Williams KL, Appel RD, Hochstrasser DF. Protein identification and analysis tools in the ExPASy server. Methods Mol Biol. 1999;112:531-52. doi: 10.1385/1-59259-584-7:531. PMID: 10027275.

5. Binder Builder

Team Members:
Corey Howe (Coreyhowe99@gmail.com)

Abstract:
I used the standard binder design pipeline of RFDiffusion for backbone generation, Soluble MPNN for sequence design, and AF2 for validation. With some promising binders in hand, I diversified and further optimized them with sequence redesign of the non-interacting residues as well as in silico directed evolution with AF2.

Reproducibility:

bioRxiv preprint doi: https://doi.org/10.64898/2026.03.03.709355; this version posted March 4, 2026. The copyright holder for this preprint (which was not certified by peer review) is the author/funder, who has granted bioRxiv a license to display the preprint in perpetuity. It is made available under aCC-BY 4.0 International license.

-Trim down the dimer CD20 structure to remove most of the transmembrane domain
-Run RFDiffusion with default settings at different binder sizes to find good designs
-Run Soluble MPNN to design sequences to the RFD diffusion backbones
-Validate the sequence designs with AlphaFold2 multimer
-Optimize the top designs by sequence redesign of non-interacting residues with soluble MPNN
-Optimize the top designs by in silico directed evolution with AlphaFold2 (code is personal fork of ColabDesign)
-Rank by plddt, i_pae, i_ptm

6. Binding Illini

Team Members:

Diwakar Shukla (diwakar@illinois.edu), Diego Kleiman, Tanner Dean, Song Yin, Lin Zhu, Joseph Clark

Abstract:

After extracting interface residues from Rituximab bound to CD20 (PDB ID: 6VJA)[73], we utilized Chroma[17] and ESM to generate sequences preserving those interface amino acids. Then, we predicted the structures of all generated sequences using Colabfold and selected the most promising models based on pLDDT and ipTM. We carried out a design loop similar to EvoBind2[74], where new sequences where generated using ProteinMPNN and structures were predicted using Colabfold. The structure prediction that minimized a loss function based on pLDDT, ipTM, iPAE, and radius of gyration was then used as input for the next round.

Reproducibility:

- Extract interface residues from Rituximab (<5 A away from CD20)
- Generate sequences of desired length by stitching together the interface residues using Chroma (conditioned on interface structure and sequence) and ESM (conditioned on interface sequence)
- Predicted structures of CD20-peptide complex using Colabfold
- Carried out iterative design loop, sequences were generated from ProteinMPNN and structures were predicted using Colabfold. Loss function was based on pLDDT, ipTM, iPAE, and radius of gyration.

Citations:

Rougé, Lionel, et al. "Structure of CD20 in complex with the therapeutic monoclonal antibody rituximab." Science 367.6483 (2020): 1224-1230.

bioRxiv preprint doi: https://doi.org/10.64898/2026.03.03.709355; this version posted March 4, 2026. The copyright holder for this preprint (which was not certified by peer review) is the author/funder, who has granted bioRxiv a license to display the preprint in perpetuity. It is made available under aCC-BY 4.0 International license.

Ingraham, John B., et al. "Illuminating protein space with a programmable generative model." Nature 623.7989 (2023): 1070-1078.

Lin, Zeming, et al. "Evolutionary-scale prediction of atomic-level protein structure with a language model." Science 379.6637 (2023): 1123-1130.

Mirdita, Milot, et al. "ColabFold: making protein folding accessible to all." Nature methods 19.6 (2022): 679-682.

Dauparas, Justas, et al. "Robust deep learning–based protein sequence design using ProteinMPNN." Science 378.6615 (2022): 49-56.

Li, Qiuzhen, Efstathios Nikolaos Vlachos, and Patrick Bryant. "Design of linear and cyclic peptide binders of different lengths only from a protein target sequence." bioRxiv (2024): 2024-06.

7. Bionova

Team Members:
Xin Wang (xin.wang.1@vanderbilt.edu), Yunchao Liu, Zhaoqian Su, Tyler Derr, Rocco Moretti, Jens Meiler

Abstract:
We implemented a standard pipeline encompassing backbone generation, inverse folding, and filtering to tackle the challenge. For binder design with the RFdiffusion model, our focus was on generating binder backbones that specifically target epitope 3 of CD20, located in the extracellular region. A diverse subset of these backbones was selected for inverse folding using ProteinMPNN, producing multiple sequence designs for each backbone. AlphaFold2 was then used to validate the 3D structures of the sequence designs by comparing the RMSD score to the original backbone. Finally, the Chai-1 model predicted the complex formed between CD20 and the filtered binder sequences. After excluding complex predictions that failed to meet the correct binding site and extracellular constraints, we ranked the binder sequences based on their iptm scores output by Chai-1.

Reproducibility:
1. Backbone Generation via RFDiffusion [1]
2. Inverse Folding via ProteinMPNN [2]
3. Relaxed Filtering via AlphaFold2 Initial Guess [2][75]
4. Final Validation of Chai [4]

bioRxiv preprint doi: https://doi.org/10.64898/2026.03.03.709355; this version posted March 4, 2026. The copyright holder for this preprint (which was not certified by peer review) is the author/funder, who has granted bioRxiv a license to display the preprint in perpetuity. It is made available under aCC-BY 4.0 International license.

[1] Watson, J.L., Juergens, D., Bennett, N.R. et al. De novo design of protein structure and function with RFdiffusion. Nature 620, 1089–1100 (2023). https://doi.org/10.1038/s41586-023-06415-8

[2] Bennett, N.R., Coventry, B., Goreshnik, I. et al. Improving de novo protein binder design with deep learning. Nat Commun 14, 2625 (2023). https://doi.org/10.1038/s41467-023-38328-5

[3] Chai Discovery team. (n.d.). Chai-1 technical report. https://chaiassets.com/chai-1/paper/technical_report_v1.pdf

8. Bryant Lab

Team Members:

Patrick Bryant (patrick.bryant@scilifelab.se), Qiuzhen Li

Abstract:

This study employed EvoBind-multimer, a novel in silico-directed evolution framework based on AlphaFold2, to design linear peptides targeting CD20. The peptides, ranging from 5 to 20 amino acids in length, were designed to function as molecular glues, bridging interactions between two proteins of interest. The CD20 sequence was extracted from a known protein complex structure, and the algorithm autonomously identified optimal binding sites for the designed peptides.

Reproducibility:

Obtain the CD20 sequence from the Structure of CD20 in complex with rituximab Fab (PDB: https://www.rcsb.org/structure/6vja).

2. Utilize EvoBind-multimer, an in-house tool, for cyclic peptide design.
3. Set peptide length ranges from 5 to 20 amino acids.
4. Configure EvoBind-multimer[74] to autonomously select optimal binding sites on CD20.
5. Run EvoBind-multimer for 1000 iterations per peptide design.
6. Execute 8 cycles per iteration.
7. Analyze resulting cyclic peptide designs for potential molecular glue function between ligase domains and CD20

9. CAD Pro

Team Members:

Aswini Javvadi (aswini@boltzmann.co), Daksh Joshi

bioRxiv preprint doi: https://doi.org/10.64898/2026.03.03.709355; this version posted March 4, 2026. The copyright holder for this preprint (which was not certified by peer review) is the author/funder, who has granted bioRxiv a license to display the preprint in perpetuity. It is made available under aCC-BY 4.0 International license.

Abstract:

In this study, we aimed to design novel peptide binders targeting the CD20 antigen by employing a multi-step workflow. Initially, sequences of 80 amino acids in length were generated using PEPMLM and ESM models with both unconditional and conditional generation methods. These sequences were subsequently filtered based on AlphaFold2 (AF2) metrics, including inter-residue predicted aligned error (iPAE), predicted local distance difference test (pLDDT), and Local Interaction Score (LIS) models, to assess structural quality. Additionally, binding affinity predictions were employed to further refine the selection process.

We then performed structure prediction and molecular docking to evaluate the interaction potential of these sequences with CD20. The best candidates were selected by focusing on those with strong interactions specifically at the epitope 3 region of CD20. Sequences showing the highest number of interactions were ranked as top candidates for further analysis.

The Final Data was further augmented with sequences generated using EvoBind2, going through the same filtration steps as above.

Reproducibility:

•Generated 80 length sequences of target based using PEP-MLM[20]

Chen T, Dumas M, Watson R, Vincoff S, Peng C, Zhao L, Hong L, Pertsemlidis S, Shaepers-Cheu M, Wang TZ, Srijay D, Monticello C, Vure P, Pulugurta R, Kholina K, Goel S, DeLisa MP, Truant R, Aguilar HC, Chatterjee P. PepMLM: Target Sequence-Conditioned Generation of Therapeutic Peptide Binders via Span Masked Language Modeling. ArXiv [Preprint]. 2024 Aug 11:arXiv:2310.03842v3. PMID: 37873004; PMCID: PMC10593082.

•Generated sequences using ESM2 https://huggingface.co/facebook/esm2_t36_3B_UR50D

•Used iPAE, PLDDT, IPTM as scoring functions to filter sequences along with binding affinity

Bennett NR, Coventry B, Goreshnik I, Huang B, Allen A, Vafeados D, Peng YP, Dauparas J, Baek M, Stewart L, DiMaio F, De Munck S, Savvides SN, Baker D. Improving de novo protein binder design with deep learning. Nat Commun. 2023 May 6;14(1):2625. doi: 10.1038/s41467-023-38328-5. PMID: 37149653; PMCID: PMC10163288.

•Structure prediction using alphafold2

•Hdock for predicted structures and interactions screening using foldX[35]

Yan, Y., Tao, H., He, J. et al. The HDOCK server for integrated protein–protein docking. Nat Protoc 15, 1829–1852 (2020). https://doi.org/10.1038/s41596-020-0312-x

•Evobind for augmenting with more sequences, going through the same steps as above

bioRxiv preprint doi: https://doi.org/10.64898/2026.03.03.709355; this version posted March 4, 2026. The copyright holder for this preprint (which was not certified by peer review) is the author/funder, who has granted bioRxiv a license to display the preprint in perpetuity. It is made available under aCC-BY 4.0 International license.

for filtration. Li, Qiuzhen, Efstathios Nikolaos Vlachos, and Patrick Bryant. "Design of linear and cyclic peptide binders of different lengths only from a protein target sequence." bioRxiv (2024): 2024-06.

10. CIIRC

Team Members:

Nikola Zadorozhny (nikola.svobodova762@gmail.com), Petr Kouba, Anton Bushuiev

Abstract:

We utilized RFDiffusion, an open-source state-of-the-art method, to generate protein structures targeting epitopes associated with current antibiotics. By employing the "Complex Model" highlighted in the publication, as well as beta model and guided diffusion via potentials, we aimed to design effective binders. Sequences were obtained using ProteinMPNN. Due to challenges in filtering and validation with standard pipeline (AF2 initial guess), we explored alternative tools like HelixFold3[32] and AlphaFold-Multimer to assess some of our designs. Additionally, we generated designs using BindCraft[18].

Reproducibility:

- Utilized RFDiffusion for protein binder generation, experimenting with beta models and guided diffusion using potentials [1].
- Focused on hot-spot selection from antibiotic-targeted epitopes.
- Designed sequences using ProteinMPNN [2].
- Encountered validation issues with standard pipelines like AlphaFold2 Initial Guess [3].
- Used HelixFold3 [4] and AlphaFold-Multimer [5] for alternative validation on some designs.
- Made additional designs using BindCraft [6].

[1] Watson, Joseph L., et al. "De novo design of protein structure and function with RFdiffusion." Nature 620.7976 (2023): 1089-1100.

[2] Dauparas, Justas, et al. "Robust deep learning–based protein sequence design using ProteinMPNN." Science 378.6615 (2022): 49-56.

[3] Bennett, N.R., Coventry, B., Goreshnik, I. et al. "Improving de novo protein binder design with deep learning." Nat Commun 14, 2625 (2023). https://doi.org/10.1038/s41467-023-38328-5

[4] Liu, Lihang, et al. "Technical Report of HelixFold3 for Biomolecular Structure Prediction." arXiv preprint arXiv:2408.16975 (2024).

[5] Evans, R. et al. "Protein complex prediction with AlphaFold-Multimer." Preprint at bioRxiv (2022). https://doi.org/10.1101/2021.10.04.463034

bioRxiv preprint doi: https://doi.org/10.64898/2026.03.03.709355; this version posted March 4, 2026. The copyright holder for this preprint (which was not certified by peer review) is the author/funder, who has granted bioRxiv a license to display the preprint in perpetuity. It is made available under aCC-BY 4.0 International license.

[6] Martin Pacesa, et al. "BindCraft: one-shot design of functional protein binders. " bioRxiv (2024). https://doi.org/10.1101/2024.09.30.615802

11. D12

Team Members:
Tadej Satler (tadej.satler@ki.si), Roman Jerala, Tjaša Mlakar, Duško Lainšček, Ajasja Ljubetič

Abstract:
Our approach to protein binder design involved a scaffold-guided workflow combining RFdiffusion, ProteinMPNN, and AlphaFold2, with final filtering based on PyRosetta metrics. We generated binder blueprints, creating adjacency matrices for binders up to 80 amino acids long. RFdiffusion was then used to target specific regions of the hCD20 antigen, exploiting both symmetric and non-symmetric designs. Symmetric diffusion allowed us to take advantage of the target's symmetry for more efficient binder generation. Binders were validated through sequence design with ProteinMPNN and structural verification via AlphaFold2, followed by optional redesign and refinement through partial diffusion.

Reproducibility:
https://github.com/kosonocky/bits-to-binders/blob/main/misc/d12_reproducibility.md

12. Evaneil

Team Members:
Neil Anthony (neil.anthony@bath.edu), Even Kiely

Abstract:
The design of novel protein binders targeting CD20 involved a multi-step computational approach. Starting with the crystal structure of CD20 from PDB entry 6VJA, we extracted the extra cellular regions of CD20 only. Key hotspot residues were manually identified to guide the design of backbone conformations that could promote binding interactions in targeted regions. Using RFdiffusion, we generated diverse backbone conformations compatible with these hotspot constraints. These backbones were then used as scaffolds for sequence design using ProteinMPNN, which predicted optimal amino acid sequences to stabilize each backbone conformation while maintaining the desired binding properties. Predicted binder sequences were scored and ranked based on their predicted stability and binding potential.

bioRxiv preprint doi: https://doi.org/10.64898/2026.03.03.709355; this version posted March 4, 2026. The copyright holder for this preprint (which was not certified by peer review) is the author/funder, who has granted bioRxiv a license to display the preprint in perpetuity. It is made available under aCC-BY 4.0 International license.

Reproducibility:
- Extract CD20 from 6VJA, and crop ECM residues -> cd20ecm
- Manually select hotspot residues to promote backbone binder in different regions
- Run RFdiffusion
- Use ProteinMPNN to generate residue on each backbone

https://github.com/RosettaCommons/RFdiffusion
https://github.com/dauparas/ProteinMPNN

13. Foldsmiths

Team Members:
Harish Srinivasan (harish.srinivasan@utsouthwestern.edu), Rongqing Yuan, Rui Guo, Jesse Durham, Jimin Pei, Qian Cong, Jian Zhou

Abstract:
We used relatively modern tools to create binders using rfdiffusion/custom model ->proteinmpnn -> af initial guess and template targeting. For hotspots we used the given interface in the pdb, hydrophobic patches and also from mmgbsa. We optimized the sequence with in silico saturated mutagenesis on alphafold metrics. We found mmgbsa proposed the best hotspots and proteinmpnn struggled to generate good interface residues based on ipae.

Reproducibility:
0)hotspots based on hydrophobic patches and mmgbsa
1)Rfdiffusion (https://www.nature.com/articles/s41586-023-06415-8)
2)proteinmpnn (https://www.science.org/doi/10.1126/science.add2187)
3)alphafold with initial guess and template targeting (https://www.nature.com/articles/s41467-023-38328-5)

14. Furman Lab

Team Members:
Ora Furman (ora.furman-schueler@mail.huji.ac.il), Julia Varga, Sarah Knapp

Abstract:
Our main design strategy was centered on the definition of promising starting fragments that form critical interactions with the receptor, which were then extended to form a stable minibinder. The starting fragments were determined using an adapted version of our PatchMAN[76] peptide docking protocol, which searches for protein structures that

bioRxiv preprint doi: https://doi.org/10.64898/2026.03.03.709355; this version posted March 4, 2026. The copyright holder for this preprint (which was not certified by peer review) is the author/funder, who has granted bioRxiv a license to display the preprint in perpetuity. It is made available under aCC-BY 4.0 International license.

contain structural motifs that are similar to patches on the receptor surface. Fragments that complement these structural motifs in these proteins are then extracted as candidate seeds, if they bind to the extracellular region.
Starting from these fragments, we used to RFdiffusion to build a stable minibinder scaffold around them (both with and without specifying hotspots based on available crystal structures, ProteinMPNN to design a matching sequence, and finally Alphafold2 to test refolding of the design into the target structure. For promising designs, we intensified the search using partial RFdiffusion. We also experimented with the newly published BindCraft protocol.
Promising designs were filtered by iPAE and rmsd, and then minimized with Rosetta[33], scored with InterfaceAnalyzer and contact molecular surface area. Then, we ranked the designs according to ipTM, ipAE, contact molecular surface area and dGcross/dSASA*100 (measures that we and others found to correlate well with successful structure predictions and designs).

Reproducibility:

1. RFDiffusion with motif-scaffolding, starting from fragments defined using PatchMAN

a. Extract starting fragments: We ran PatchMAN[1] to find proteins with structural match to surface patches. From these matches we extracted complementing fragments that could be used as starting points for binder design. We masked the non-extracellular regions, as we did not want the binders to touch the membrane-embedded regions.

b. Build full binder starting from these fragments: We ran RFDiffusion[2] with motif-scaffolding and sequence inpainting of the extracted template in two different modes:

1. General, without specified hotspots

2. Targeted: with specified hotspots, defined using Rosetta alanine scanning[3][77] of crystal structures (Y161 and N171 of CD20)

c. Filter promising backbone candidates: From the backbone outputs, we removed those with high radius of gyration (>16A), and/or with significant contact of the membrane regions (>15% of binder residues), and with low contact to the extracellular regions (<20% of binder residues)

d. Design sequences for backbones: We ran ProteinMPNN using the ColabDesign[4] implementation (excluding cysteine, temperature 0.15), which also provided an interface to refold with AlphaFold2[5]. For refolding, we used templates for CD20, initial guess (as implemented in Benett et al.[6]), AlphaFold2-multimer-v3 model 1 and recycles of 3.

e. Select promising candidates: We removed designs with low predicted alignment errors (ipae > 10A), minimized them with Rosetta[7], and calculated Rosetta interface scores. We later further removed designs with rmsd > 3 between the template and the refolded structure.

bioRxiv preprint doi: https://doi.org/10.64898/2026.03.03.709355; this version posted March 4, 2026. The copyright holder for this preprint (which was not certified by peer review) is the author/funder, who has granted bioRxiv a license to display the preprint in perpetuity. It is made available under aCC-BY 4.0 International license.

2. Partial diffusion on accepted designs

a. To increase the diversity of the designed backbones, we performed additional partial diffusion using RFDiffusion. partial_T was set to 10, and 10 new backbones were generated from original designs that were selected after manual inspection of the best refolding designs. The same ProteinMPNN (but with temperature of 0.3) and AlphaFold2 refolding protocol was run, and the same cutoffs applied as before.

b. A second round of partial diffusion was conducted on the top 15 performing designs from the initial partial diffusion, following the same methodology as in part A.

3. BindCraft

BindCraft[8] was run until reaching 10 accepted designs with default parameters, using the provided Colab notebook. 2 designs with iptm values of equal or better of the methods described above were included in the final set.

4. Ranking candidates:

For ranking the sequences, we calculated the rank of each design by four metrics: ipTM, ipAE, contact molecular surface area and dGcross/dSASAx100. We summed up these ranks for each designed sequence, and sorted the designs according to them.

Bibliography

1. Khramushin, A. et al. Matching protein surface structural patches for high-resolution blind peptide docking. Proc Natl Acad Sci USA 119, e2121153119 (2022).
2. Watson, J. L. et al. De novo design of protein structure and function with RFdiffusion. Nature 620, 1089–1100 (2023).
3. Kortemme, T., Kim, D. E. & Baker, D. Computational alanine scanning of protein-protein interfaces. Sci. STKE 2004, pl2 (2004).
4. GitHub - sokrypton/ColabDesign: Making Protein Design accessible to all via Google Colab! https://github.com/sokrypton/ColabDesign.
5. Evans, R. et al. Protein complex prediction with AlphaFold-Multimer. BioRxiv (2021) doi:10.1101/2021.10.04.463034.
6. Bennett, N. R. et al. Improving de novo protein binder design with deep learning. Nat. Commun. 14, 2625 (2023).
7. Leman, J. K. et al. Macromolecular modeling and design in Rosetta: recent methods and frameworks. Nat. Methods 17, 665–680 (2020).
8. Pacesa, M. et al. BindCraft: one-shot design of functional protein binders. BioRxiv (2024) doi:10.1101/2024.09.30.615802.

bioRxiv preprint doi: https://doi.org/10.64898/2026.03.03.709355; this version posted March 4, 2026. The copyright holder for this preprint (which was not certified by peer review) is the author/funder, who has granted bioRxiv a license to display the preprint in perpetuity. It is made available under aCC-BY 4.0 International license.

15. LBM

Team Members:

Fernando Meireles (fernando.meireles@epfl.ch), Parth Bibekar, Benedikt Singer

Abstract:

Knowing that CD20 is hard target to design binders against, we decided to perform some Molecular Dynamics on the target to get a wider variety of starting structures. We also assumed that flexible chains comprising the epitope might have different conformations one of them maybe being a bit wider allowing for a bigger binding site. After clustering the MD trajectories a total of 29 cluster remained from which 8 were selected based on a more open confirmation. A subsequent RFDiffusion run was started to generate a total of 5,000 structures where either randomly the true target structure or an MD-derived structure was used. Additionally from a set of hotspots, two hotspots were randomly sampled.

Inverse folding was performed using either ProteinMPNN with or CARBonAra[25] with their respective default parameters. For each RfDiffusion-generated structure 5 sequences were generated.

After this localcolabold's version of AlphaFold2 was applied followed by Alphafold-multimer where the true structure of the target was provided. The thus produced metrics (pAE, pLDDT) were used for the submission.

(A short remark about the delayed hand-in: After the deadline extension we had some internal misunderstandings of the timezones. We hope that our submission will still be considered)

Reproducibility:

- Target structures: 6VJA (https://www.science.org/doi/10.1126/science.aaz9356)
- MD-parametrisation: GROMACS 2024.02 (https://www.sciencedirect.com/science/article/pii/S2352711015000059)
- Structure-binder generation: RFDiffusion (https://www.nature.com/articles/s41586-023-06415-8)
- Inverse folding:
    - Protein-MPNN (https://doi.org/10.1126%2Fscience.add2187)
    - CARBonAra (https://www.biorxiv.org/content/10.1101/2023.06.19.545381v1.full.pdf)
- Validation:
    - Colabfold (https://www.nature.com/articles/s41592-022-01488-1)
        - Localcolabfold (https://github.com/YoshitakaMo/localcolabfold)
        - AlphaFold2 (https://www.nature.com/articles/s41586-021-03819-2)
    - AlphaFold-Multimer (https://www.biorxiv.org/content/10.1101/2021.10.04.463034v1)

bioRxiv preprint doi: https://doi.org/10.64898/2026.03.03.709355; this version posted March 4, 2026. The copyright holder for this preprint (which was not certified by peer review) is the author/funder, who has granted bioRxiv a license to display the preprint in perpetuity. It is made available under aCC-BY 4.0 International license.

16. Molecule Masters

Team Members:

Karl Lundquist (klundquist@gmail.com), Dion Whitehead, Arjun Singh, Abel Gurung, Amardeep Singh

Abstract:

We developed a computational pipeline to design protein binders targeting CD20. Starting with RFDiffusion, we generated initial binder structures and optimized their sequences using ProteinMPNN alongside Rosetta FastRelax to optimize for folding into the designed structure as well as complementarity with the extracellular region of CD20. To do this we carried out a series of rounds in which we balanced exploration and exploitation by carrying the top performers into subsequent rounds and creating new binders in each round as well. In each round, we predicted the 3D structures using AlphaFold2. We removed binders that were poorly predicted by AF2 and also those that would protrude into the membrane after proper alignment. In order to accurately assess the binding stability, we used an energy minimization and molecular dynamics simulation procedure to relax the side-chains. Using this equilibrated structure we evaluated binding stability by calculating the total energy and the non-bonded interaction energy between the binder and CD20. We also estimated binding free energies (ΔG) using Prodigy. We also calculated binder radius of gyration in order to preference compact designs. Our scoring function combined binder radius of gyration, interaction energy, and ΔG, adjusted by total energy, to effectively rank the binders. Designs meeting our criteria for energy thresholds and RMSD advanced through the rounds, improving their stability and affinity. Finally, we consolidated and re-ranked the top binders from all rounds. By integrating RFDiffusion for structure generation and ProteinMPNN with FastRelax for sequence design, along with careful filtering steps, our pipeline efficiently produces high-affinity binders specific to the extracellular region of CD20.

Reproducibility:

- RFDiffusion to generate initial protein binder structures
    - Watson, J.L., Juergens, D., Bennett, N.R. et al. De novo design of protein structure and function with RFdiffusion. Nature 620, 1089–1100 (2023). https://doi.org/10.1038/s41586-023-06415-8
- ProteinMPNN, FastRelax, AF2 procedure for binder design
    - Bennett, N.R., Coventry, B., Goreshnik, I. et al. Improving de novo protein binder design with deep learning. Nat Commun 14, 2625 (2023). https://doi.org/10.1038/s41467-023-38328-5
    - Dauparas, J., Anishchenko, I., Bennett, N., et al. (2022). Robust deep learning–based protein sequence design using ProteinMPNN. Science, 378(6615),

bioRxiv preprint doi: https://doi.org/10.64898/2026.03.03.709355; this version posted March 4, 2026. The copyright holder for this preprint (which was not certified by peer review) is the author/funder, who has granted bioRxiv a license to display the preprint in perpetuity. It is made available under aCC-BY 4.0 International license.

49–56. https://doi.org/10.1126/science.add2187
- Jumper, J., Evans, R., Pritzel, A., et al. (2021). Highly accurate protein structure prediction with AlphaFold. Nature, 596(7873), 583–589. https://doi.org/10.1038/s41586-021-03819-2

- Molecular Dynamics for side chain relaxation[28]

- Eastman, P., Swails, J., Chodera, J.D., et al. (2017). OpenMM 7: Rapid development of high performance algorithms for molecular dynamics. PLOS Computational Biology, 13(7), e1005659. https://doi.org/10.1371/journal.pcbi.1005659

- Prodigy for binding free energy calculation[36,78]

- Vangone, A., & Bonvin, A.M.J.J. (2015). Contacts-based prediction of binding affinity in protein–protein complexes. eLife, 4, e07454. https://doi.org/10.7554/eLife.07454
- Xue, L.C., Rodrigues, J.P., Kastritis, P.L., Bonvin, A.M.J.J., & Vangone, A. (2016). PRODIGY: a web server for predicting the binding affinity of protein–protein complexes. Bioinformatics, 32(23), 3676–3678. https://doi.org/10.1093/bioinformatics/btw514

17. N6 / Team Anonymous

Team Members:
Nicole Chiang (nicole.ch27@gmail.com)

Abstract:
We used an approach combing domain knowledge and machine learning. We generate a library of peptide sequences incorporating known binding motifs, emphasizing the integration of an aromatic cradle composed of tryptophan and tyrosine residues, along with hydrogen bond donors like asparagine or serine. Each sequence is evaluated using ESMFold to determine its pLDDT score, and sequences with low scores are filtered out to retain those likely to exhibit better structural integrity. We hope this simple but combined approach may address the challenges of stability and folding in peptide binders while ensuring that specific structural features enhance binding affinity.

Reproducibility:

1. Sequence Generation:

* Generate peptide sequences incorporating known binding motifs based on scaffolds that are known to interact with proline rich domains (which is sort of present in the extracellular loops of CD20), such as SH3 and WW domain[79].
* Design the sequences to include an "aromatic cradle" with tryptophan and tyrosine residues for enhanced recognition of proline residues.

2. Incorporation of Structural Features:

*Integrate additional hydrogen bond donors, such as asparagine or serine, to interact

bioRxiv preprint doi: https://doi.org/10.64898/2026.03.03.709355; this version posted March 4, 2026. The copyright holder for this preprint (which was not certified by peer review) is the author/funder, who has granted bioRxiv a license to display the preprint in perpetuity. It is made available under aCC-BY 4.0 International license.

with backbone carbonyl groups, improving stability.

3. Charged Residue Complementation:
*Introduce complementary charged residues around the binding motifs to enhance binding specificity.

4. Model Selection:
* Utilize ESMFold, a protein structure prediction model, to evaluate the generated peptide sequences.

5. pLDDT Score Calculation:
* Input each generated sequence into ESMFold to obtain pLDDT scores, which indicate structural integrity.

6. Filtering Based on pLDDT Scores:
*Filter the generated sequences by retaining only those with higher pLDDT scores, ensuring a focus on sequences likely to exhibit better structural stability[80].

Citations:
1. Ball, Linda J., et al. "Recognition of proline-rich motifs by protein–protein-interaction domains." Angewandte Chemie International Edition 44.19 (2005): 2852-2869
2. Lin, Zeming, et al. "Language models of protein sequences at the scale of evolution enable accurate structure prediction." BioRxiv 2022 (2022): 500902.
3. Tejera-Nevado, Paloma, et al. "Analysis of the Confidence in the Prediction of the Protein Folding by Artificial Intelligence." International Conference on Practical Applications of Computational Biology & Bioinformatics. Cham: Springer Nature Switzerland, 2023.

18. Nucleate UK London

Team Members:
Jakub Lála (jakublala@gmail.com), David Miller, Stefano Angioletti-Uberti

Abstract:
We employed the high-level programming language from (Hie et al. 2022)[81], later implemented as a part of the BAGEL package[46]. Two design strategies were employed, detailed below.

First, full 80-mer sequences were designed against the CD20 dimer. After the Monte Carlo (MC) optimization, SolubleMPNN[24] was employed to generate alternative

bioRxiv preprint doi: https://doi.org/10.64898/2026.03.03.709355; this version posted March 4, 2026. The copyright holder for this preprint (which was not certified by peer review) is the author/funder, who has granted bioRxiv a license to display the preprint in perpetuity. It is made available under aCC-BY 4.0 International license.

sequences from the same backbones. Specifically, we retrieved the top-100 designs from the MC optimization based on the final value of the objective/energy function. These final sequences were not de-duplicated, so the total number of unique designs from this part is not 100. We then further repainted each backbone many times with SolubleMPNN, and took the top-100 based on the confidence score in SolubleMPNN. In total, this gave 200 sequence submissions from the first design strategy.

Second, 20-mer segments were designed using the MC optimization against the CD20 monomer epitope alone. RFDiffusion[16] was then used to design the 40-mer linker between the two monomer binders, where we alternated various monomer binders with one another (e.g., a monomer binder A and B would be combined as AA, AB, BB). The linkers alone were then inpainted with SolubleMPNN to get the full 80-mer sequence. The monomer binder segments were thus kept conserved from the ESMFold-driven MC optimization. As before, we took the top-300 designs based on the confidence score from SolubleMPNN.

We also validated some of our best designs by visually inspecting 10 ns molecular dynamics, but due to a lack of time did not use this extensively to do any ranking or filtering.

Reproducibility:
1. ESMFold optimization design[81]
2. Earlier version of BAGEL[46]
3. SolubleMPNN alternative sequence design[24]
4. MD Validation with OpenMM[28]

19. Perez Lab Gators

Team Members:

Jokent Gaza (jokent.gaza@ufl.edu), Alberto Perez, Bhumika Singh, Yisel Martinez Noa, Qianchen Liu, Rangana De Silva

Abstract:

Binders for the CD20 extracellular domain were generated using the RFDiffusion to ProteinMPNN-FastRelax pipeline. As an orthogonal assay, the generated sequences, along with the CD20 dimer, were folded using AlphaFold2 Initial Guess. Our initial plan was to use the binding epitopes of three antibodies (rituximab, ofatumumab, and obinutuzumab) as initial motifs for RFDiffusion. Inpainting would then generate a scaffold that preserves the binding epitope and/or interacts with other residues in the CD20 binding domain. However, although the pipeline produced stable monomers, the

bioRxiv preprint doi: https://doi.org/10.64898/2026.03.03.709355; this version posted March 4, 2026. The copyright holder for this preprint (which was not certified by peer review) is the author/funder, who has granted bioRxiv a license to display the preprint in perpetuity. It is made available under aCC-BY 4.0 International license.

predictions for the complex were suboptimal. An initial cut-off of pAE < 15 was set, and none of the 18000 designs (6000 per antibody) met this threshold.

For plan B, we decided to start from a completely de novo design strategy. Out of the 3000 designs, only 5 passed the pAE threshold. Of these, only two (called DN1 and DN2) had all the residues in the extracellular region of CD20. For both DN1 and DN2, we extracted the residues that directly interact with CD20. We then performed another round of RFDiffusion to generate 1000 designs that have a scaffold for these residues. For DN1, we obtained only one good design, while DN2 generated 65. We then extended the number of designs for DN2 to 8000, resulting in 211 designs. These involved optimizing either the entire sequence or just the non-binding residues using ProteinMPNN. We also optimized the non-binding residues of DN1,giving a final list of 223 sequences. A quick sanity test was then performed to test if these are stable proteins. Specifically, we performed AF2 predictions for both the monomer and the full CAR construct. We surmised that good binders should retain their structure both as a monomer and in the full CAR (RMSD < 2 Å) and should be compact (RoG < 14 Å). From these, we were able to further reduce the number of designs to 193 (12 from DN1 and 181 from DN2).

To reach the 500-sequence target, we tried optimizing the final 181 sequences from DN2 using proteinMPNN. We performed three runs, for a total of new 543 sequences. Of these sequences, 166 passed the pAE filter. We also revisited the initial predictions with antibody epitopes and de novo designs. We took all the sequences that have pLDDT > 85 for the binder, and performed the same sanity test. We then ranked the designs based on their pLDDT score as a monomer to decide which of them will be included in the submission. To introduce diversity in our submission, we decided to prioritize the results from the antibody epitopes, as they have more varied topology. In total, the final 500 sequences included 359 good binders, 60 stable monomers from antibody epitopes, and 81 stable monomers from de novo predictions.

Our github page can be found in: https://github.com/PDNALab/BioML

<u>Reproducibility:</u>

1. Extracted the binding epitopes of the following antibodies – rituximab (6VJA), ofatumumab (6Y92), and obinutuzumab (6Y97).
2. Generated 6000 designs for each antibody—3000 using the Complex_base model of RFDiffusion and another 3000 using the Complex_beta model.[82] (1-5)
3. Filtered the designs using a pAE threshold (pAE < 15), but no designs met the criteria.

bioRxiv preprint doi: https://doi.org/10.64898/2026.03.03.709355; this version posted March 4, 2026. The copyright holder for this preprint (which was not certified by peer review) is the author/funder, who has granted bioRxiv a license to display the preprint in perpetuity. It is made available under aCC-BY 4.0 International license.

4. Generated 3000 de novo designs for the CD20 structure from 6VJA using the Complex_beta model with reduced noise (i.e., with denoiser.noise_scale_ca and denoiser.noise_scale_frame set to zero) during sampling. Only five designs passed the filter, and only two (called DN1 and DN2) were considered reasonable binders.
5. Extracted the binding epitope of DN1 and generated 1000 additional designs. Only one design passed the threshold.
6. Tried to optimize the non-binding residues of DN1 using proteinMPNN. The final list of designs from DN1 is 12.
7. Extracted the binding epitope of DN2 and generated 1000 additional designs, resulting in 65 designs. Extended this to 8000 designs, to get a list of 211 DN2 designs.
8. The combined total of designs from DN1 and DN2 was 223. A sanity test was conducted by comparing the binder as a monomer and in the full CAR, using RMSD < 2 Å and a radius of gyration < 14 Å as criteria. The final list of DN1 and DN2 designs was reduced to 193.
9. Optimized the 181 sequences from DN2, resulting in an additional 166 sequences.
10. Performed the same sanity test on all designs (both antibody-based and de novo) with pLDDT > 85. For the final sequence list, we prioritized designs with antibody epitopes and pLDDT > 90.
11. The final list of sequences included 359 good binders, 60 stable monomers from antibody epitopes, and 81 stable monomers from de novo predictions.

References:

1. Watson, J. L. et al. De novo design of protein structure and function with RFdiffusion. Nature 2023, 620, 1089–1100.
2. Dauparas, J. et al. Robust deep learning–based protein sequence design using Protein-MPNN. Science 2022, 378, 49–56.
3. Bennett, N. R.; Coventry, B.; Goreshnik, I.; Huang, B.; Allen, A.; Vafeados, D.; Peng, Y. P.; Dauparas, J.; Baek, M.; Stewart, L.; DiMaio, F.; De Munck, S.; Savvides, S. N.; Baker, D. Improving de novo protein binder design with deep learning. Nature Communications 2023, 14 .
4. Jumper, J. et al. Highly accurate protein structure prediction with AlphaFold. Nature 2021, 596, 583–589.
5. Gaza, J., Roth, M., Montelione, G., & Perez, A. Hybrid AI/physics pipeline for miniprotein binder prioritization: application to the BRD3 ET domain. Chemical Communications 2025.

bioRxiv preprint doi: https://doi.org/10.64898/2026.03.03.709355; this version posted March 4, 2026. The copyright holder for this preprint (which was not certified by peer review) is the author/funder, who has granted bioRxiv a license to display the preprint in perpetuity. It is made available under aCC-BY 4.0 International license.

20. Perforators

Team Members:
Vinayak Annapure (vinayak.annapure@monash.edu), Vikrant Parmar, Huan Koh, Saeideh Moradvandi, Yizhen Zheng

Abstract:
We analyzed the known binders against CD3 and used those residues as a constraint for diffusing potential binders for CD20. We further used ProteinMPNN to solve the reverse folding problem for the designed binders and used ESMFolds confidence score to select the potential binders

Reproducibility:
N/A

24. The Physicist

Team Members:
Rohit Satija (rohitsatija0092@gmail.com)

Abstract:
Solved structures of CD20 in complex with antibodies are available on the PDB: [6VJA](https://www.rcsb.org/structure/6vja), [6Y92](https://www.rcsb.org/structure/6Y92), and [6Y97](https://www.rcsb.org/structure/6Y97). In all three cases, the hotspot region of CD20 present inside the binding region is the short macrocyclic polypeptide connected by a disulfide bridge `NCEPANPSEKNSPSTQYC` (166-184, chain D, 6vja). For this reason, we decided to focus on this epitope as our binder target. We chose to take a rational design approach to pick fragments of antibodies closest to the target epitope in the PDB structures using a nearest neighbor search algorithm. These sequences were ranked according to a perplexity score computed from embeddings calculated using an open source 33 layer ESM-2 model with 650M params, originally trained on UniRef50. Top 500 sequences were submitted for experimental validation. This work resulted in a number of [jupyter notebooks](https://github.com/rohitium/bioml-challenge-2024/tree/main/notebooks) and a [rational protein design package](https://github.com/rohitium/rational_protein_design) that could be useful for future protein design studies.

Reproducibility:
To replicate the final result, i.e.
[submission.fasta](https://github.com/rohitium/bioml-challenge-2024/blob/main/results/s

bioRxiv preprint doi: https://doi.org/10.64898/2026.03.03.709355; this version posted March 4, 2026. The copyright holder for this preprint (which was not certified by peer review) is the author/funder, who has granted bioRxiv a license to display the preprint in perpetuity. It is made available under aCC-BY 4.0 International license.

ubmission.fasta), follow these steps:

1. **Binder Design:** This step is accomplished using the [rational_design.ipynb](https://github.com/rohitium/bioml-challenge-2024/blob/main/notebooks/rational_design.ipynb) notebook. Briefly, the python package [rational_protein_design](https://pypi.org/project/rational-protein-design/) contains a class called `BinderDesigner` that takes in the following attributes:
    * `pdb_file`: Path to an existing PDB structure of the bound complex (Str)
    * `chain_id`: Chain ID of the target (Str)
    * `target_residues_range`: Range of residues on Target chain to design binders for (Tuple(Int))
    * `neighbor_chain_id`: Chain ID of the candidate bound molecule (Str)
    * `N`: Length of binder (Int)
    * `num_seq`: Number of binders to design (Int)

Provided these attributes to an object of the `BinderDesigner` class, the `design_binder()` method runs all the functions necessary to design binders for the target sequence and store them in a provided fasta file path.

2. **Rank Binders:** This step is accomplished using the [compute_embeddings.ipynb](https://github.com/rohitium/bioml-challenge-2024/blob/main/notebooks/compute_embeddings.ipynb) notebook. Briefly, we create embeddings for all candidate binder sequences produced in the previous step using Facebook's `esm2_t33_650M_UR50D` model (https://github.com/facebookresearch/esm?tab=readme-ov-file#main-models-you-should-use-). Next, we compute perplexity for each individual sequence (https://en.wikipedia.org/wiki/Perplexity) that is a metric describing how well the model predicts the existence of the sequence. We rank order the binder sequences using this score and save the top 500 sequences to file.

22. Picaso Protein

Team Members:
Elias Sanchez (ersanchezo@gmail.com), Nuria Mitjavila, Sofiia Hoian, Alejandro Diaz, Marko Ludaic, Jinling Huang, Nader Ibrahim, Yong Youn Kwon

Abstract:
We aimed to design high-affinity binders targeting Rituximab's epitope (C171-174) using three computational strategies. First, we used nanobodies and language models with Raygun to generate optimized binder sequences of 80 aa tailored to Rituximab's epitope. Second, we explored novel sequence spaces through de novo design without

bioRxiv preprint doi: https://doi.org/10.64898/2026.03.03.709355; this version posted March 4, 2026. The copyright holder for this preprint (which was not certified by peer review) is the author/funder, who has granted bioRxiv a license to display the preprint in perpetuity. It is made available under aCC-BY 4.0 International license.

existing templates. Third, we generated cyclic peptides and replaced Rituximab's CDR2 and CDR3 regions with random peptide pairs, refining these using custom metrics. A rules-based mutation strategy was applied, mutating residues based on interaction interface and structural metrics like pLDDT and iPAE. AlphaFold2 Multimer was used to model and select optimal candidates after each mutation round.

Reproducibility:

-We aimed to design high-affinity binders targeting Rituximab's epitope (C171-174) using three distinct computational strategies. [1][83]

-First, we utilized existing nanobodies and language models with Raygun to generate optimal binder sequences and reduce the size to 80 aa, specifically tailored to Rituximab's epitope on CD20. [2-4][22,84,85]

-Second, we performed de novo design of binders to explore novel sequence spaces without relying on existing templates.

-Third, we generated cyclic peptides with high predicted Local Distance Difference Test (pLDDT) scores and replaced the CDR2 and CDR3 regions of Rituximab's heavy chain with randomly selected peptide pairs, followed by sequence refinement using custom scripts and metrics to select optimal candidates. [6]

-To optimize these initial sequences, we applied a rules-based mutation strategy: residues at the interaction interface were mutated at a low rate favoring substitutions with positive BLOSUM scores; neighboring residues were subjected to higher mutation rates based on their pLDDT and inter-residue Predicted Aligned Error (iPAE) values; non-interacting and non-neighboring residues experienced even higher mutation rates aimed at increasing their pLDDT values. Neighboring and interaction residues where determined with getcontacts.[5]

-After each round of in silico mutations, subsets of offspring sequences were generated and modeled using AlphaFold2 Multimer to predict their three-dimensional structures. This iterative process applied evolutionary pressure, selecting sequences with high confidence according to both pLDDT and iPAE metrics, ultimately refining candidate binders toward optimal interaction with Rituximab's epitope.

Get_contacts: https://github.com/getcontacts/getcontacts/tree/master

[1]Klein, C., Lammens, A., Schäfer, W., Georges, G., Schwaiger, M., Mössner, E., … Niederfellner, G. (2012). Epitope interactions of monoclonal antibodies targeting CD20 and their relationship to functional properties. mAbs, 5(1), 22–33. https://doi.org/10.4161/mabs.22771

[2] Liu et al. Selection and characterization of single domain antibodies against human CD20
doi.org/10.1016/j.molimm.2016.09.013

bioRxiv preprint doi: https://doi.org/10.64898/2026.03.03.709355; this version posted March 4, 2026. The copyright holder for this preprint (which was not certified by peer review) is the author/funder, who has granted bioRxiv a license to display the preprint in perpetuity. It is made available under aCC-BY 4.0 International license.

[3] Daneels W, Van Parys A, Huyghe L, Rogge E, De Rouck S, Christiaen R, Zabeau L, Taveirne S, Van Dorpe J, Kley N, Cauwels A, Depla E, Tavernier J, Offner F. High efficacy of huCD20-targeted AcTaferon in humanized patient derived xenograft models of aggressive B cell lymphoma. Exp Hematol Oncol. 2024 Jun 3;13(1):59. doi: 10.1186/s40164-024-00524-4. PMID: 38831452; PMCID: PMC11145843.

[4]Devkota, K., Shonai, D., Mao, J., Soderling, S. H., & Singh, R. (2024). Miniaturizing, Modifying, and Augmenting Nature's Proteins with Raygun. bioRxiv, 2024-08.
[5]Get_contacts: https://github.com/getcontacts/getcontacts/tree/master
[6]Kosugi, T.; Ohue, M. Design of Cyclic Peptides Targeting Protein–Protein Interactions Using[86]

23. Schoeder Lab

Team Members:
Moritz Ertelt (moritz.ertelt@gmail.com), Max Beining, Dominic Rieger, Dieter Hoffmann, Tom Schlegel, Max Lingner, Vivian Haas

Abstract:
The workflow initiated with the crystal structure (PDB: 6VJA) and molecular dynamics (MD) simulations to model the outer loops. Principal component analysis (PCA) identified three predominant loop configurations, and both, the original crystal structure and a representative structure of each cluster, were selected for subsequent steps. Using RFdiffusion, binders were generated by targeting trimmed extracellular domains in dimeric configurations. Hotspot regions, including crucial interaction residues from CD20 and Rituximab, guiding peptide generation and de-novo binder design. Filtering criteria such as contact order, shape complementarity, and secondary structure were applied to identify potential high-quality backbones suitable for sequence design. Sequence design was performed using ProteinMPNN across different temperature levels, with AlphaFold-based target-binder-complex predictions supporting scoring and selection of the designs. Final binders were iteratively refined using metrics like pae_interaction, pLDDT, Rosetta's dG_separated, and additional LIA and LIS filters. In parallel, an AlphaFold hallucination approach was employed to generate peptides of varying lengths. These peptides were scaffolded using RFdiffusion and optimized iteratively for binding against CD20. In addition, peptide repeats with linker regions were introduced to enhance avidity-like binding properties.

Reproducibility:
Crystal structure (PDB: 6VJA) and an MD simulation for the outer loops as a starting

bioRxiv preprint doi: https://doi.org/10.64898/2026.03.03.709355; this version posted March 4, 2026. The copyright holder for this preprint (which was not certified by peer review) is the author/funder, who has granted bioRxiv a license to display the preprint in perpetuity. It is made available under aCC-BY 4.0 International license.

point, MD + PCA results in three main loop configurations (use only one cluster representative for further steps)
Using RFdiffusion [1] in various approaches, only working on trimmed input structures (extracellular part, dimer configuration)
Generating binders by providing different hotspot regions (e.g., the original key interaction residues from CD20 with Rituximab [3], unpublished surface clustering algorithm)
Peptide generation + scaffolding
De-novo binder design
Perform filtering by pyrosetta and specific cutoffs for contact order, shape complementarity, secondary structure etc.
ProteinMPNN [4] for sequence generation using different temperatures and Alphafold initial guess for target-binder prediction and scoring based on the pipeline from [5]
Final designs were filtered using pae_interaction, plddt and Rosetta [6][34] dG_separated
In addition to that, some designs were filtered using the metrics suggested by [7], namely LIA and LIS
Binders were optimized iteratively with ProteinMPNN and Alphafold initial guess by using good scoring models as inputs or partially diffused variations of them
Using an inversion of the AlphaFold structure prediction network [2] and their protocol to generate peptides of different lengths
generated peptides were scaffolded using RFdiffusion
Peptide repeats with linker regions were designed to achieve avidity-like binding

[1] Watson, Joseph L et al. “De novo design of protein structure and function with RFdiffusion.” Nature vol. 620,7976 (2023): 1089-1100. doi:10.1038/s41586-023-06415-8
[2] Goverde, Casper A et al. “De novo protein design by inversion of the AlphaFold structure prediction network.” Protein science : a publication of the Protein Society vol. 32,6 (2023): e4653. doi:10.1002/pro.4653
[3] Lionel Rougé et al. “Structure of CD20 in complex with the therapeutic monoclonal antibody rituximab.” Science367, 1224-1230(2020).DOI:10.1126/science.aaz9356
[4] Dauparas, J et al. “Robust deep learning-based protein sequence design using ProteinMPNN.” Science (New York, N.Y.) vol. 378,6615 (2022): 49-56. doi:10.1126/science.add2187
[5] Bennett, N.R., Coventry, B., Goreshnik, I. et al. Improving de novo protein binder design with deep learning. Nat Commun 14, 2625 (2023). https://doi.org/10.1038/s41467-023-38328-5
[6] Leaver-Fay, Andrew et al. “ROSETTA3: an object-oriented software suite for the simulation and design of macromolecules.” Methods in enzymology vol. 487 (2011): 545-74. doi:10.1016/B978-0-12-381270-4.00019-6
[7] Ah-Ram Kim, Yanhui Hu, Aram Comjean, Jonathan Rodiger, Stephanie E. Mohr,

bioRxiv preprint doi: https://doi.org/10.64898/2026.03.03.709355; this version posted March 4, 2026. The copyright holder for this preprint (which was not certified by peer review) is the author/funder, who has granted bioRxiv a license to display the preprint in perpetuity. It is made available under aCC-BY 4.0 International license.

Norbert Perrimon.”Enhanced Protein-Protein Interaction Discovery via AlphaFold-Multimer”. bioRxiv 2024.02.19.580970; doi: https://doi.org/10.1101/2024.02.19.580970

24. SNU LCDD

Team Members:
Jinwoong Song (jinungsong@snu.ac.kr), Hakyung Lee, Haelyn Kim

Abstract:
We applied multi-track for successful binder design.

For backbone design, we applied 1) de novo, 2) grafting antibody CDR loops 3) grafting CDRH3 only.

We generated the protein sequences with proteinMPNN and filtered out the sequences with Rosetta modeling and scoring.

The remained sequences were modeled the complex structure with AlphaFold2.

Lastly, We run short Molecular Dynamics simulation for the complex structure in order to sort the designs in binding affinity to CD20.

Reproducibility:
RFdiffusion
ProteinMPNN
Rosetta
AlphaFold2
Molecular Dynamics

25. Tumor Inhibitors

Team Members:
Ashish Makani (spiff007@gmail.com), Cianna Calia, Rana A. Barghout, Matthew Williams, Javier Marchena-Hurtado, Vivian Chu

Abstract:
Expanding the set of known protein binders to human CD20 holds promise for medical applications such as CAR T-cell therapies for B-cell malignancies. However, CD20 presents a challenging target for de novo binder design for multiple reasons: The

bioRxiv preprint doi: https://doi.org/10.64898/2026.03.03.709355; this version posted March 4, 2026. The copyright holder for this preprint (which was not certified by peer review) is the author/funder, who has granted bioRxiv a license to display the preprint in perpetuity. It is made available under aCC-BY 4.0 International license.

extracellular loops (ECL1/ECL2) protruding out of the cell membrane provide relatively small surfaces for binding and contain many hydrophilic residues [1], and judging by the slightly lower pLDDT in these loops in CD20's AlphaFoldDB structure [2, 3][87,88], the exposed regions may also have some degree of flexibility. We therefore constructed a multi-tiered approach to attempt to maximize the success rate of CD20 binders we generated with state-of-the-art deep learning tools. First, we used RFdiffusion's [4] motif scaffolding functionality to create a minimal target structure consisting of segments of both CD20 monomers from PDB 6VJA [1], united into a single asymmetrical chain by two diffused glycine linkers. With this structure as the provided target, we used RFdiffusion's "beta" complex parameters to generate over 25,000 80-residue binder backbones. To allow us to generate many sequences per backbone without wasting time on low-quality designs, we filtered our backbones using Biopython [5] to ensure contact with Tyr161 in CD20's ECL2, to prohibit contact with the nearest diffused linker, and to avoid binders with excessively long helices and/or too few target contacts. We also eliminated any backbones incompatible with the cell membrane by aligning each target-binder complex to a membrane-embedded structure of CD20 from MemProtMD [6] and discarding any backbones with more than 10 heavy-atom clashes between the binder and the lipids. For all backbones passing this filtering, we used the ColabDesign framework [7] to run ProteinMPNN [8] to generate 64 sequences each with a sampling temperature of 0.000001, followed by screening with AlphaFold2 (using model_1_ptm with initial guess, target templating, three recycles, and no MSAs) [9, 10]. Because we found binders with pae_interaction < 10 to be rare, we prioritized further exploration of the sequence and structure space surrounding "near hits" that had pae_interaction < 22 and RMSD < 8 Å with respect to the designed structure; for all backbones giving one or more near-hit sequences we generated and screened 64 additional sequences with a sampling temperature of 0.1, and for some promising candidates we generated still more sequences and in several cases also diversified the binder backbone with partial diffusion. With these methods we compiled 1,323 binder sequences with pae_interaction < 22 and RMSD < 8 Å, which we ranked by pae_interaction (and re-ranked by RMSD within pae_interaction windows of 1.0). After discarding those with pLDDT < 80, those with long alanine stretches, and those within 20 amino acids of higher ranking candidates, 868 sequences remained. Our submission contains the highest ranking 500 of these remaining sequences.

<u>Reproducibility:</u>

- RFdiffusion [4] to create minimal target structure from PDB 6VJA [1]
- RFdiffusion [4] to generate binder backbones
- Backbone filtering with Biopython [5][89] and check for membrane clashes using structure from MemProtMD [6]
- ProteinMPNN [8] to generate sequences (via ColabDesign [7])

bioRxiv preprint doi: https://doi.org/10.64898/2026.03.03.709355; this version posted March 4, 2026. The copyright holder for this preprint (which was not certified by peer review) is the author/funder, who has granted bioRxiv a license to display the preprint in perpetuity. It is made available under aCC-BY 4.0 International license.

- AlphaFold2 [9] with initial guess [10] to screen designs (via ColabDesign [7])
- Additional sequence generation / partial diffusion for best designs
- Rank best sequences by pae_interaction and RMSD

References:

[1] Rougé et al. Science, 2020. https://www.science.org/doi/10.1126/science.aaz9356
[2] Varadi et al. Nucleic Acids Research, 2022. https://academic.oup.com/nar/article/50/D1/D439/6430488
[3] Tunyasuvunakool et al. Nature, 2021. https://www.nature.com/articles/s41586-021-03828-1
[4] Watson et al. Nature, 2023. https://www.nature.com/articles/s41586-023-06415-8
[5] Cock et al. Bioinformatics, 2009. https://academic.oup.com/bioinformatics/article/25/11/1422/330687
[6] Newport et al. Nucleic Acids Research, 2019. https://academic.oup.com/nar/article/47/D1/D390/5173663
[7] Ovchinnikov et al. ColabDesign Framework: https://github.com/sokrypton/ColabDesign
[8] Dauparas et al. Science, 2022. https://www.science.org/doi/full/10.1126/science.add2187
[9] Jumper et al. Nature, 2021. https://www.nature.com/articles/s41586-021-03819-2
[10] Bennett et al. Nature Communications, 2023. https://www.nature.com/articles/s41467-023-38328-5

26. Undergraduate Design

Team Members:

Max Witwer (max.h.witwer@vanderbilt.edu), Nathaniel Greenwood, Jie Chen, Hannah Stewart, Elliot Cole

Abstract:

This study aims to develop high-affinity mini-binders for the extracellular domain of CD20. CD20, a transmembrane protein overexpressed in many B cell malignancies, is a prime target for novel therapeutics. CAR T-cell therapy, an immunotherapy technique, involves engineering a patient's T-cells to express chimeric antigen receptors (CARs) that recognize antigens like CD20, enabling them to destroy malignant B cells. Computational protein design has enabled the creation of novel protein backbones that can interact with a range of structures, facilitating the development of mini-binders to enhance targeting in CAR T-cell therapy. However, current computational limitations and CD20's unique structure make it a challenging binding problem due to the hydrophilic nature of its extracellular domain. Current design methods rely on hydrophobic residues

bioRxiv preprint doi: https://doi.org/10.64898/2026.03.03.709355; this version posted March 4, 2026. The copyright holder for this preprint (which was not certified by peer review) is the author/funder, who has granted bioRxiv a license to display the preprint in perpetuity. It is made available under aCC-BY 4.0 International license.

for energetically favorable binders, leading to two challenges: first, unconstrained design generates proteins that bind mainly to the hydrophobic transmembrane domain; second, binders to the extracellular region have low predicted binding scores by tools like AlphaFold2. To address these issues, a design campaign used RFdiffusion, ProteinMPNN, and AlphaFold2. CD20’s structure (PDB entry 6VJA) was downloaded, and the rituximab Fab was deleted using PyMOL. Initially, 80-residue “seed structures” were created in reasonable locations, approximating previously designed Fabs like rituximab (6VJA) and obinutuzumab (6Y9A).

To alleviate these issues, the structure was mutated to drive the AI models to produce better results. First, phospholipid-facing residues in the transmembrane domain were mutated to aspartate, producing a pseudo-solubilized protein to discourage AI models from designing transmembrane region binders. Next, six key residues (GLU 174, SER 177, SER 179 on each CD20 dimer) were mutated to LEU 174, VAL 177, VAL 179. High-temperature partial diffusion (60-100 noise steps) guided the model to bind to the desired domain, producing high-affinity CD20 binders, AlphaFold2 interface predicted aligned error (PAE). To create mini-binders for the native target, an evolutionary algorithm was used to slowly mutate the 80 residue structure toward more favorable results. The structure of CD20 between generations was slowly mutated back to its native hydrophilic form. Designed backbones were mutated using low-temperature partial diffusion (15-18 noise steps) and sequences were mutated with ProteinMPNN; the produced designs were selected for interface root mean squared deviation (RMSD) and interface PAE. This was repeated across nine generations. A final refinement step was performed using predictions from an extended version of AlphaFold2 dubbed AlphaFold multimer, which is designed to specifically predict protein-protein interactions. The final tenth round of recycling was performed directly on multimer predictions and the resulting sequences were ranked based on AlphaFold multimer interface PAE; additionally, cutoffs were imposed for the interface RMSD, binder predicted local distance difference test (pLDDT) and interface predicted template modeling (TM) score. The final ranked pool of results contains 500 predicted sequences.

<u>Reproducibility:</u>

Steps:

1) Use crystal structure 6VJA, remove rituximab and portions of the transmembrane domain (TD) that are far from the extracellular domain. Mutate any remaining outward facing TD residues to aspartate. Mutate hydrophilic residues in the extracellular binding pocket to hydrophobic residues with similar geometries.

2) Use PyMol builder to build four helices in the extracellular binding pocket

3) Use partial RFdiffusion[1] to make these helices into biochemically reasonable backbones. Use high partial T to sample diverse backbones that are not simply a four helical bundle. Use ProteinMPNN[2] and AlphaFold2 initial guess[3].

bioRxiv preprint doi: https://doi.org/10.64898/2026.03.03.709355; this version posted March 4, 2026. The copyright holder for this preprint (which was not certified by peer review) is the author/funder, who has granted bioRxiv a license to display the preprint in perpetuity. It is made available under aCC-BY 4.0 International license.

4) Choose best designs[4][90] based on pae_interaction, mutate 1-2 residues back, recycle those designs, and repeat until the native structure is recovered.
5) Run AlphaFold multimer[5][91] on the predicted structures and perform a final round of partial diffusion, rank the final designs based on AlphaFold multimer interface PAE and RMSD with cutoffs for desired metrics.

Citations:
[1]Watson, J. L. et al. De novo design of protein structure and function with RFdiffusion. Nature 620, 1089–1100 (2023).
[2]Dauparas, J. et al. Robust deep learning–based protein sequence design using ProteinMPNN. Science (1979) 378, 49–56 (2022).
[3]Jumper, J. et al. Highly accurate protein structure prediction with AlphaFold. Nature 596, 583–589 (2021).
[4]Cao, L. et al. Design of protein-binding proteins from the target structure alone. Nature 605, 551–560 (2022).
[5]Bryant, P. & Noé, F. Improved protein complex prediction with AlphaFold-multimer by denoising the MSA profile. PLoS Comput Biol 20, e1012253 (2024).

27. Virtue Therapeutics

Team Members:
Rajat Punia (rajat@vtx.bio), Akshay Chenna

Abstract:
Used BindCraft model for binder designs with default filter parameters.

Reproducibility:
1. Receptor residues that make H-bond with the antibody in PDB 6Y92 are taken as hotspot residues.
2. PDB 6Y92 is chosen to decide as hotspot to design binders that can bind to either chain of the receptor and not the interface. This results in small reduction in entropic penalty of binding.
3. Binders are designed with default settings using BindCraft. None of the design was able to pass the default filters. Best designs from the trajectory are used for submission.

28. Zist Rayanesh

Team Members:
Seyed Alireza Hashemi (salireza111@gmail.com), Kaveh Nasrollahi, Fatemeh Nasiri

bioRxiv preprint doi: https://doi.org/10.64898/2026.03.03.709355; this version posted March 4, 2026. The copyright holder for this preprint (which was not certified by peer review) is the author/funder, who has granted bioRxiv a license to display the preprint in perpetuity. It is made available under aCC-BY 4.0 International license.

Abstract:
Design begins by analyzing the target molecule to identify binding hotspots. At this initial stage, hotspots are selected based on hydrophobic patches and known antibody epitope sites involved in binding. For each of the six identified hotspots, 100 binders, ranging from 12 to 18 residues, are designed using RFdiffusion. This is followed by generating 8 sequences per binder with MPNN+Fastrelax and then using AlphaFold-Multimer for structure prediction. Validation of binders is based on metrics like binding energy and iPAE, and the best hotspot is chosen for further optimization. This step was constrained by time and computational resources.
After selecting a pool of promising mini-binders, the next phase involves designing scaffolds for them using RFdiffusion. Subsequently, the binders are dimerized to target both accessible sides of the molecule, provided it has a symmetrical structure.
The final designs must conform to a standard format with 80 residues. Binders requiring fewer than six residues to bridge gaps are connected using a Gly-Gly-Ser linker. For larger gaps, the RFdiffusion pipeline is run again to optimize the design.
308 sequences were submitted due to the limitation of computational unit (i3 12100f + RTX 3060 + 16 GB of RAM)

Reproducibility:
- Finding Hotspots (https://www.acrobiosystems.com/L-965-CD20.html?gad_source=1)
- RFdiffusion for binder design (https://github.com/RosettaCommons/RFdiffusion)
- MPNN for designing sequences (https://github.com/dauparas/ProteinMPNN)
- ColabFold (Local + MMseq server) (https://github.com/sokrypton/ColabFold)
- Pyrosetta as a filtering step (https://docs.rosettacommons.org/docs/latest/application_documentation/analysis/interface-analyzer)
